# Supplementary material for: Multi-organ single-cell analysis reveals an on/off switch system with potential for personalized treatment of immunological diseases
Source: Cell Rep Med. 2023 Feb 28;4(3):100956. doi: 10.1016/j.xcrm.2023.100956 (PMC10040389; doi:10.1016/j.xcrm.2023.100956)
Supplement: Document S1. Figures S1–S7 and Table S1 [file mmc1.pdf]

Cell Reports Medicine, Volume *4*

Supplemental information

Multi-organ single-cell analysis reveals an on/off switch system with potential for personalized treatment of immunological diseases

Sandra Lilja, Xinxiu Li, Martin Smelik, Eun Jung Lee, Joseph Loscalzo, Pratheek Bellur Marthanda, Lang Hu, Mattias Magnusson, Oleg Sysoev, Huan Zhang, Yelin Zhao, Christopher Sjöwall, Danuta Gawel, Hui Wang, and Mikael Benson

Supplementary information

Supplementary Figures:

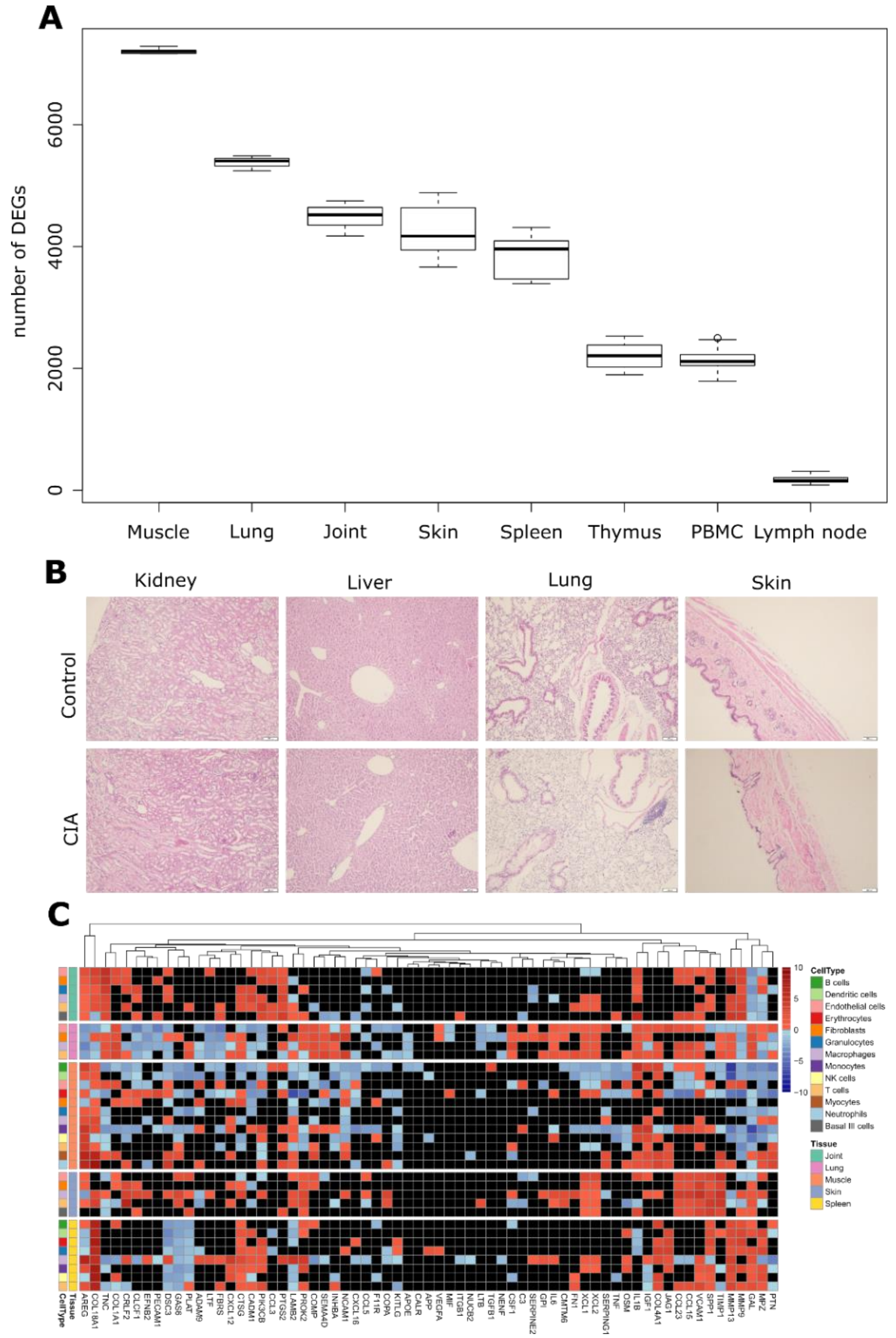

Figure S1. The number of differentially expressed genes, H&E analysis, the FCs of all URs from the different organs. Related to Figure 2.
(A) Boxplot showing the number of DEGs produced between a subset of 40 CIA and 40 healthy cells from each of the different organs. Cells were sampled 10 times, following differential expression analysis using Monocle, as described in[S1]. The boxes indicate the first and third quartile, with whiskers. The line represents the median value, and outliers are marked as circles. **(B)** Representative H&E images of kidney, liver, lung, and skin from healthy control mice and CIA mice, shown at a magnification of 100 × (scar bar 100 μm). **(C)** Heatmap showing the FCs of all URs identified by the NicheNet analyses, in all five organs.

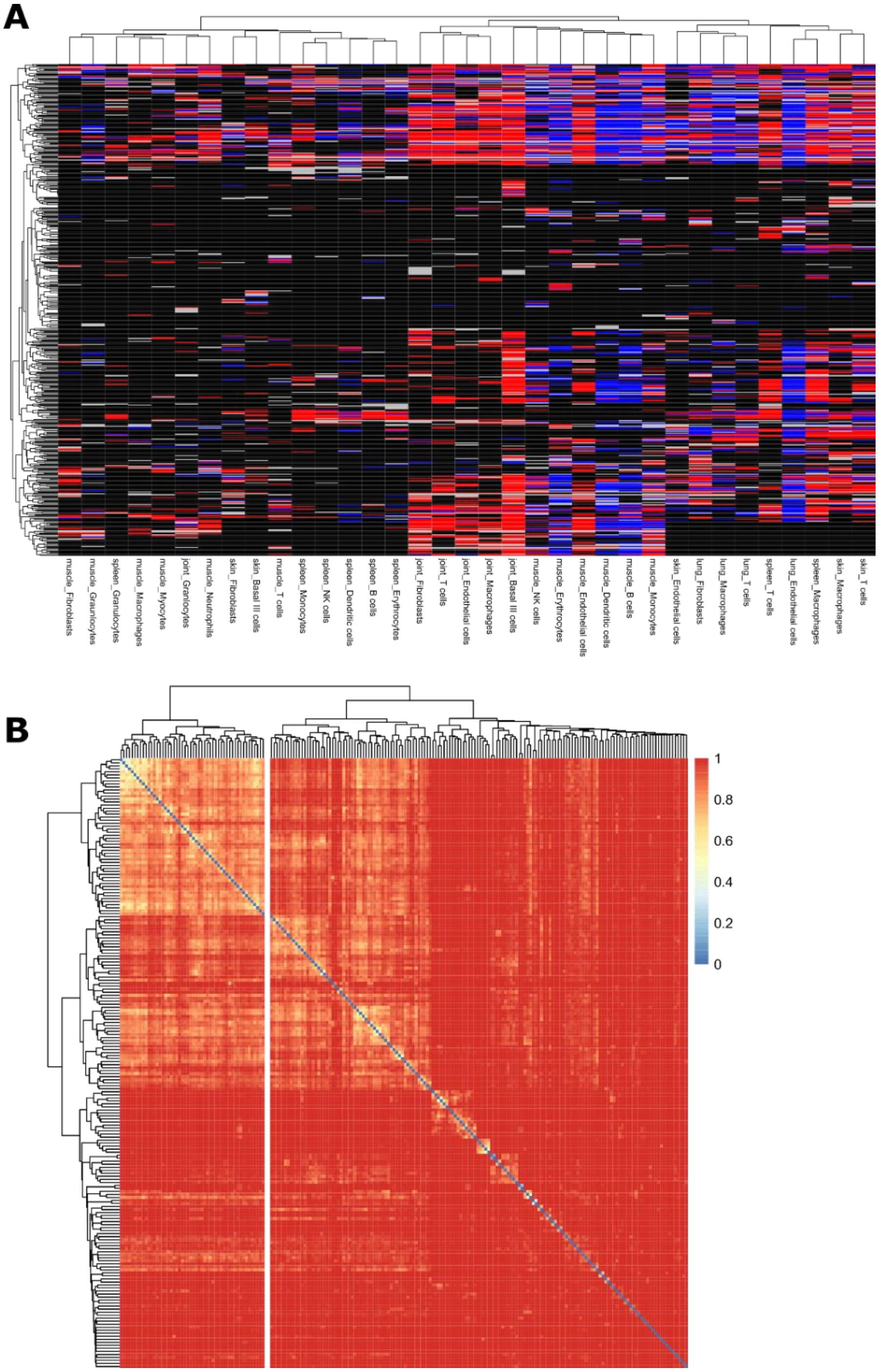

Figure S2. Pathways enrichment analysis and Connective Pathway Analysis. Related to STAR methods. (A) Pathways being significantly enriched in at least one cell type and organ. Heatmap showing the z-scores from pathway enrichment analysis. The color indicates the pathways inferred direction where red = activated, blue = inhibited, grey = unknown, black = not significant. (B) Connective Pathway Analysis on KEGG pathways. Two main programs were identified- program 1 (right) and program 2 (left). Color represents 1-Jaccard Index.

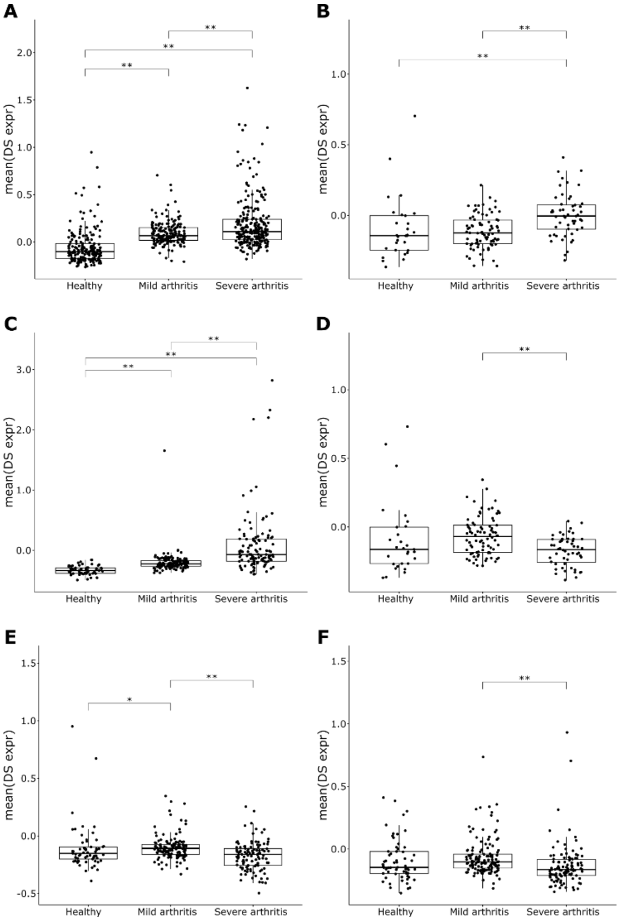

Figure S3. Mean standardized expression level per cell in healthy, mild arthritic, and severe arthritic joint of the downstream genes (DS) of upstream regulators. Related to STAR methods and Figure 2.
(A) TNF in Fibroblasts, (B) IL1B in T cells, (C) MPZ in Macrophages, (D) LTF in T cells, (E) LTF in Macrophages, and (F) DSC3 in Macrophages. Each dot represents an individual cell. The FDR-corrected *p* values were calculated using Wilcoxon rank sum test, * $p < 0.05$ and ** $p < 0.01$.

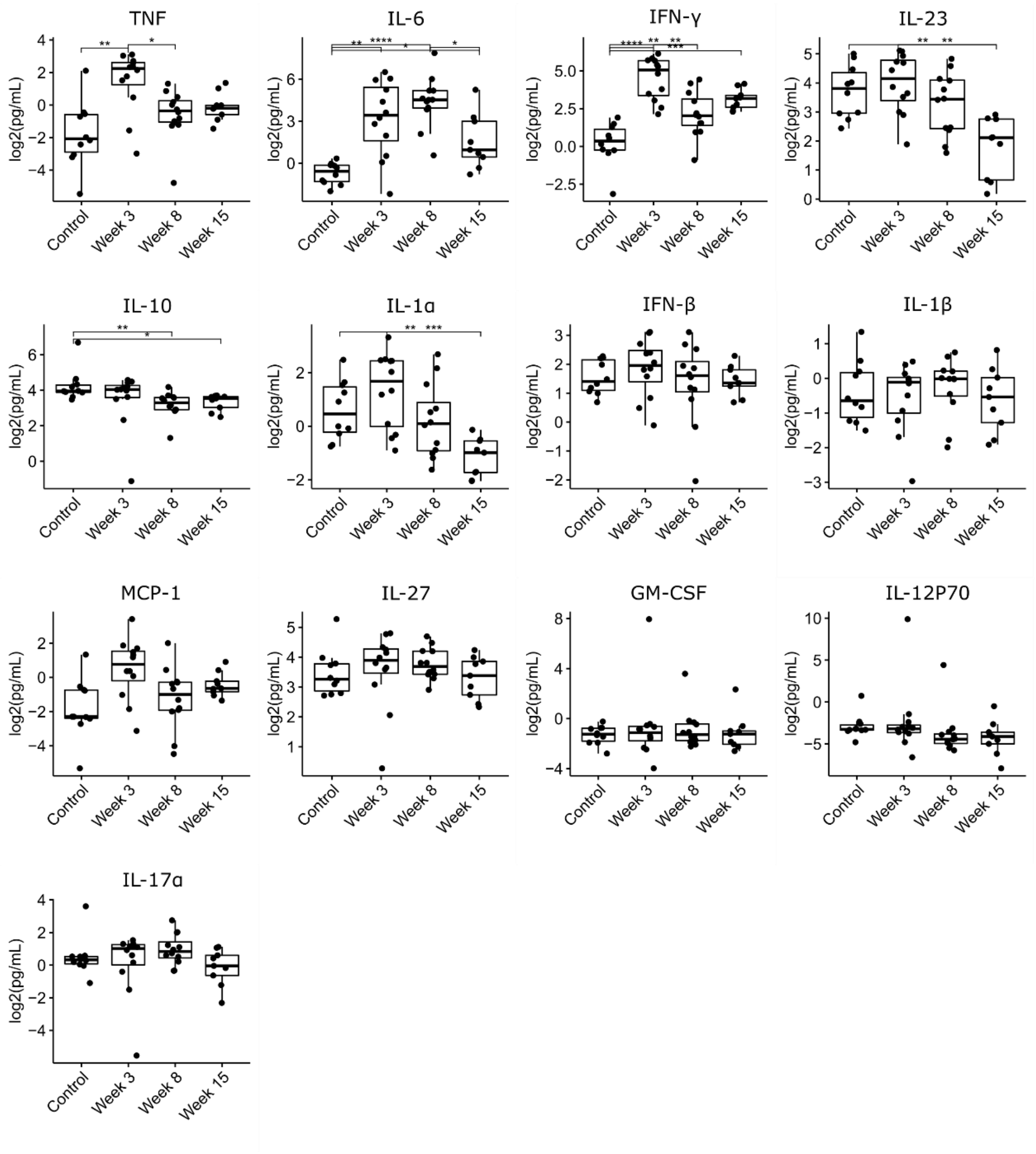

Figure S4. Time series analysis of cytokine protein levels in sera from CIA and control mice. Related to STAR methods.

The proteins were measured at different time points after collagen stimulation and in healthy control mice. *p < 0.05, **p < 0.01, ***p < 0.001, ****p < 0.0001.

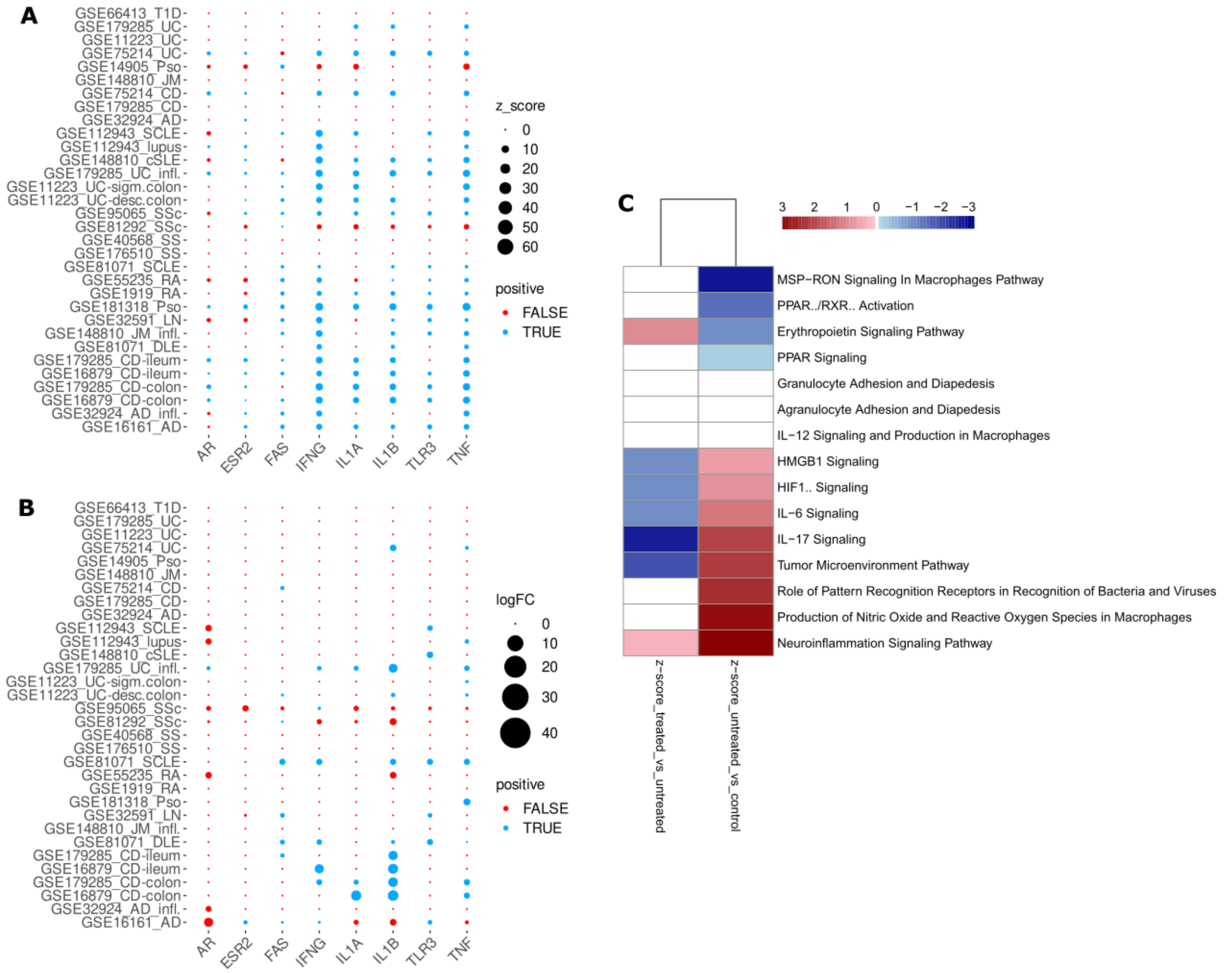

Figure S5. Predicted activities, fold changes of the shared URs and predicted direction of pathways of IMID sub-program 1.6. Related to the STAR methods and Figure 7.

Dot plot showing the (A) predicted activities and (B) fold changes, of the shared URs of IMID_SP1.6. The URs are presented in columns and the datasets are presented in rows. Blue represents increased activity or fold-change, and red represents the opposite. The size of a dot represents (A) z score or (B) log fold change(logFC). (C) Predicted direction of pathways in IMID sub-program 1.6 due to disease (right) and after treatment (left). A positive z-score (red) indicates that the pathway was upregulated due to disease or treatment, and a negative z-score (blue) indicates that the pathway was downregulated. The directions are inferred based on pathway analysis of GSE52746.

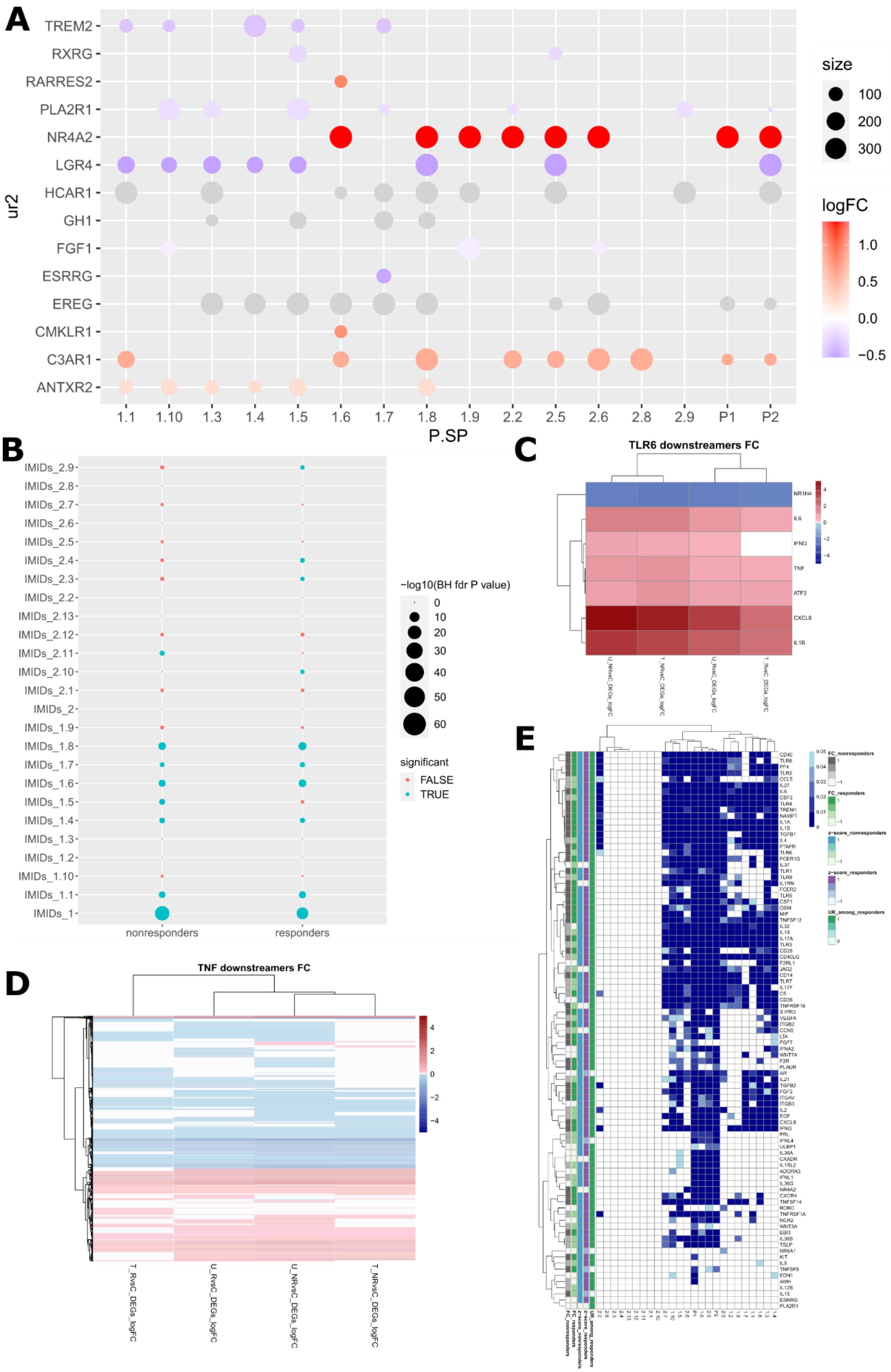
A
TREM2
RXRG
RARRES2
PLA2R1
NR4A2
LGR4
HCAR1
GH1
FGF1
ESRRG
EREG
CMKLR1
C3AR1
ANTXR2
ur2
1.1
1.10
1.3
1.4
1.5
1.6
1.7
1.8
1.9
2.2
2.5
2.6
2.8
2.9
P1
P2
P.SP
size
100
200
300
logFC
1.0
0.5
0.0
−0.5
B
IMIDs_2.9
IMIDs_2.8
IMIDs_2.7
IMIDs_2.6
IMIDs_2.5
IMIDs_2.4
IMIDs_2.3
IMIDs_2.2
IMIDs_2.13
IMIDs_2.12
IMIDs_2.11
IMIDs_2.10
IMIDs_2.1
IMIDs_2
IMIDs_1.9
IMIDs_1.8
IMIDs_1.7
IMIDs_1.6
IMIDs_1.5
IMIDs_1.4
IMIDs_1.3
IMIDs_1.2
IMIDs_1.10
IMIDs_1.1
IMIDs_1
nonresponders
responders
−log10(BH fdr P value)
0
10
20
30
40
50
60
significant
FALSE
TRUE
C
TLR6 downstreamers FC
NR1H4
IL6
IFNG
TNF
ATF3
CXCL8
IL1B
D
TNF downstreamers FC
4
2
0
−2
−4
T_RvsC_DEGs_logFC
U_RvsC_DEGs_logFC
U_NRvsC_DEGs_logFC
T_NRvsC_DEGs_logFC
E

Figure S6. Analysis of responder, non-responder, treated/untreated responder, treated/untreated non-responder and control. Related to STAR methods.

(A) Upstream regulators of the subprograms predicted to be regulated by *TNF* that were not changed after the anti-TNF medication (GSE92415). URs are presented in rows, sub-programs in columns. Dots represent significant overlap between UR DS and TNF DS (Fisher Exact test, left-tailed, corrected for multiple testing, Methods). Size represents Odds Ratio (OR), and color of the nodes shows the FC of UR in the UC responders to anti-TNF vs. healthy controls (red denotes upregulation in UC, purple downregulation, whereas grey denotes that the UR was not significantly differentially expressed or not measured on the microarray GSE92415). (B) Main- and subprogram association of pathways enriched among the DEGs for non-responders vs. control and responders vs. control, right-tailed Fisher's exact test. (C) and (D) Fold changes of the (C) TLR 6 and TNF (D) downstream genes between (from left to right): treated responders vs. control, untreated responders vs. control, untreated non-responders vs. control, and treated non-responders vs. control. The colored gradient indicates significant ($p < 0.05$) positive (red) and negative (blue) FCs. (E) Enrichment analysis between the downstream genes of each UR predicted among the non-responders and the downstream genes of TNF. The blue gradient indicates the FDR-corrected p value. White indicates nonsignificant $p > 0.05$. The enrichment analysis was performed by Fisher's exact test.

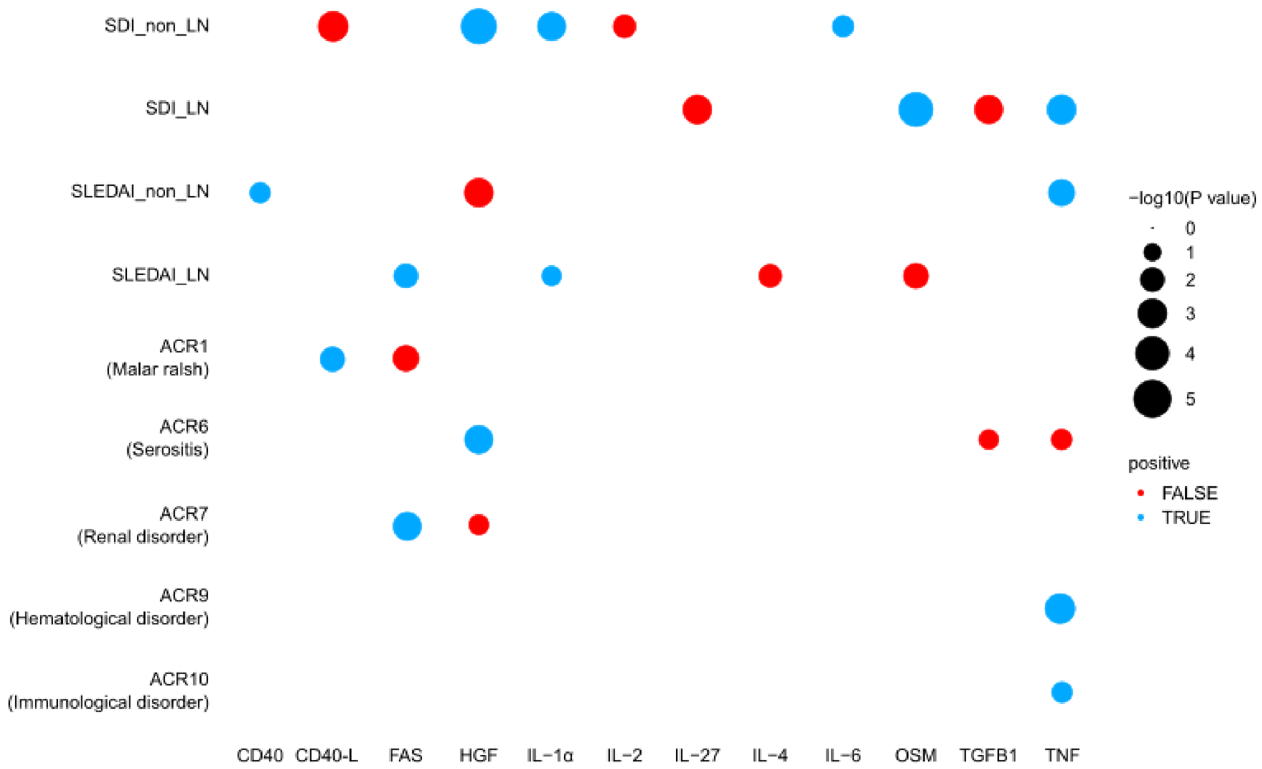

Figure S7. Summary of regression models. Related to the STAR methods.

The URs are presented in columns and the disease types are presented in rows. Blue represents a positive relationship between UR and patient phenotype. Red represents a negative relationship. The size of a dot represents the level of significance (related to Data S1_ACR1_6_7_9_10, S1_SDI and S1_SLEDAI).

Supplementary Tables:

Table S1. The marker genes used for cell type identification. Related to STAR methods and Figure 2.

**Cell type**	**Marker genes**	**References**
B cells	*Ms4a1, Cd22, Cd19*	[S2]
Dendritic cells	*Cebpa, Nfe2, Ifit1, Cd7, SiglecH*	[S3-6]
Endothelial cells	*Cdh5, Pecam1, Fabp4*	*[S7]*
Erythrocytes	*Alas2, Car2, Cpox, Hba-a1, Hbb-bt, Alad, Tfrc, Hba-a2*	[S8]
Fibroblasts	*Col1a2, Col3a1, Fbln2, Fstl1, Gsn, Mmp2, Sparc, Vim*	[S9]
Granulocytes	*Camp, Ngp, S100a8, S100a9*	[S10]
Macrophages	*C1qb, C1qc, Cd14, Cd68, Ctss, Cxcl2, F13a1, Gatm, Lyz2, Rgs1*	[S11]
Monocytes	*Ccl6, Cxcl3, Il1b, F13a1, Ccr2*	[S4,8]
NK cells	*Klrk1, Il18rap, Gzma*	[S12]
T cells	*Bcl11b, Il7r, Itk, Lef1*	[S13]
Myocytes	*Bgn, Rgs16, Palld, Rgs5, Cpe, Sgce, Fhl1, Cd151, Adi1, Adgrf5*	[S2,14]
Basal III cells	*Krt14, Krt5, Mt2, BC100530, Fosl1, Ptgs2*	[S15]
Neutrophils	*Cd14, S100a8, S100a9, Ly6g, Mmp9, Ltf*	[S2,16,17]

References:

S1. Li, X., Lee, E.J., Lilja, S., Loscalzo, J., Schäfer, S., Smelik, M., Strobl, M.R., Sysoev, O., Wang, H., Zhang, H., et al. (2022). A dynamic single cell-based framework for digital twins to prioritize disease genes and drug targets. Genome Med *14*, 48. 10.1186/s13073-022-01048-4.

S2. Giordani, L., He, G.J., Negroni, E., Sakai, H., Law, J.Y.C., Siu, M.M., Wan, R., Corneau, A., Tajbakhsh, S., Cheung, T.H., and Le Grand, F. (2019). High-Dimensional Single-Cell Cartography Reveals Novel Skeletal Muscle-Resident Cell Populations. Mol Cell *74*, 609-621 e606. 10.1016/j.molcel.2019.02.026.

S3. Paul, F., Arkin, Y., Giladi, A., Jaitin, D.A., Kenigsberg, E., Keren-Shaul, H., Winter, D., Lara-Astiaso, D., Gury, M., Weiner, A., et al. (2015). Transcriptional Heterogeneity and Lineage Commitment in Myeloid Progenitors. Cell *163*, 1663-1677. 10.1016/j.cell.2015.11.013.

S4. Jaitin, D.A., Weiner, A., Yofe, I., Lara-Astiaso, D., Keren-Shaul, H., David, E., Salame, T.M., Tanay, A., van Oudenaarden, A., and Amit, I. (2016). Dissecting Immune Circuits by Linking CRISPR-Pooled Screens with Single-Cell RNA-Seq. Cell *167*, 1883-1896 e1815. 10.1016/j.cell.2016.11.039.

S5. Qie, C., Jiang, J., Liu, W., Hu, X., Chen, W., Xie, X., and Liu, J. (2020). Single-cell RNA-Seq reveals the transcriptional landscape and heterogeneity of skin macrophages in Vsir(-/-) murine psoriasis. Theranostics *10*, 10483-10497. 10.7150/thno.45614.

S6. Giladi, A., Paul, F., Herzog, Y., Lubling, Y., Weiner, A., Yofe, I., Jaitin, D., Cabezas-Wallscheid, N., Dress, R., Ginhoux, F., et al. (2018). Single-cell characterization of haematopoietic progenitors and their trajectories in homeostasis and perturbed haematopoiesis. Nat Cell Biol *20*, 836-846. 10.1038/s41556-018-0121-4.

S7. Kalucka, J., de Rooij, L., Goveia, J., Rohlenova, K., Dumas, S.J., Meta, E., Conchinha, N.V., Taverna, F., Teuwen, L.A., Veys, K., et al. (2020). Single-Cell Transcriptome Atlas of Murine Endothelial Cells. Cell *180*, 764-779 e720. 10.1016/j.cell.2020.01.015.

S8. Tusi, B.K., Wolock, S.L., Weinreb, C., Hwang, Y., Hidalgo, D., Zilionis, R., Waisman, A., Huh, J.R., Klein, A.M., and Socolovsky, M. (2018). Population snapshots predict early haematopoietic and erythroid hierarchies. Nature *555*, 54-60. 10.1038/nature25741.

S9. Gladka, M.M., Molenaar, B., de Ruiter, H., van der Elst, S., Tsui, H., Versteeg, D., Lacraz, G.P.A., Huibers, M.M.H., van Oudenaarden, A., and van Rooij, E. (2018). Single-Cell Sequencing of the Healthy and Diseased Heart Reveals Cytoskeleton-Associated Protein 4 as a New Modulator of Fibroblasts Activation. Circulation *138*, 166-180. 10.1161/circulationaha.117.030742.

S10. Cochain, C., Vafadarnejad, E., Arampatzi, P., Pelisek, J., Winkels, H., Ley, K., Wolf, D., Saliba, A.E., and Zernecke, A. (2018). Single-Cell RNA-Seq Reveals the Transcriptional Landscape and Heterogeneity of Aortic Macrophages in Murine Atherosclerosis. Circ Res *122*, 1661-1674. 10.1161/CIRCRESAHA.117.312509.

S11. Farmer, D.T., Nathan, S., Finley, J.K., Shengyang Yu, K., Emmerson, E., Byrnes, L.E., Sneddon, J.B., McManus, M.T., Tward, A.D., and Knox, S.M. (2017). Defining epithelial cell dynamics and lineage relationships in the developing lacrimal gland. Development *144*, 2517-2528. 10.1242/dev.150789.

S12. Bezman, N.A., Kim, C.C., Sun, J.C., Min-Oo, G., Hendricks, D.W., Kamimura, Y., Best, J.A., Goldrath, A.W., Lanier, L.L., and Immunological Genome Project, C. (2012). Molecular definition of the identity and activation of natural killer cells. Nat Immunol *13*, 1000-1009. 10.1038/ni.2395.

S13. Park, J., Shrestha, R., Qiu, C., Kondo, A., Huang, S., Werth, M., Li, M., Barasch, J., and Susztak, K. (2018). Single-cell transcriptomics of the mouse kidney reveals potential cellular targets of kidney disease. Science *360*, 758-763. 10.1126/science.aar2131.

S14. Rubenstein, A.B., Smith, G.R., Raue, U., Begue, G., Minchev, K., Ruf-Zamojski, F., Nair, V.D., Wang, X., Zhou, L., Zaslavsky, E., et al. (2020). Single-cell transcriptional profiles in human skeletal muscle. Sci Rep *10*, 229. 10.1038/s41598-019-57110-6.

S15. Haensel, D., Jin, S., Sun, P., Cinco, R., Dragan, M., Nguyen, Q., Cang, Z., Gong, Y., Vu, R., MacLean, A.L., et al. (2020). Defining Epidermal Basal Cell States during Skin Homeostasis and Wound Healing Using Single-Cell Transcriptomics. Cell Rep *30*, 3932-3947 e3936. 10.1016/j.celrep.2020.02.091.

S16. Volberding, P.J., Xin, G., Kasmani, M.Y., Khatun, A., Brown, A.K., Nguyen, C., Stancill, J.S., Martinez, E., Corbett, J.A., and Cui, W. (2021). Suppressive neutrophils require PIM1 for metabolic fitness and survival during chronic viral infection. Cell Rep *35*, 109160. 10.1016/j.celrep.2021.109160.

S17. Grieshaber-Bouyer, R., Radtke, F.A., Cunin, P., Stifano, G., Levescot, A., Vijaykumar, B., Nelson-Maney, N., Blaustein, R.B., Monach, P.A., Nigrovic, P.A., and ImmGen, C. (2021). The neutrotime transcriptional signature defines a single continuum of neutrophils across biological compartments. Nat Commun *12*, 2856. 10.1038/s41467-021-22973-9.
