# Supplementary material for: Multi-organ single-cell analysis reveals an on/off switch system with potential for personalized treatment of immunological diseases
Source: Cell Rep Med. 2023 Feb 28;4(3):100956. doi: 10.1016/j.xcrm.2023.100956 (PMC10040389; doi:10.1016/j.xcrm.2023.100956)
Supplement: Document S2. Article plus supplemental information [file mmc8.pdf]

Cell Reports Medicine

Article

Multi-organ single-cell analysis reveals an on/off switch system with potential for personalized treatment of immunological diseases

Graphical abstract

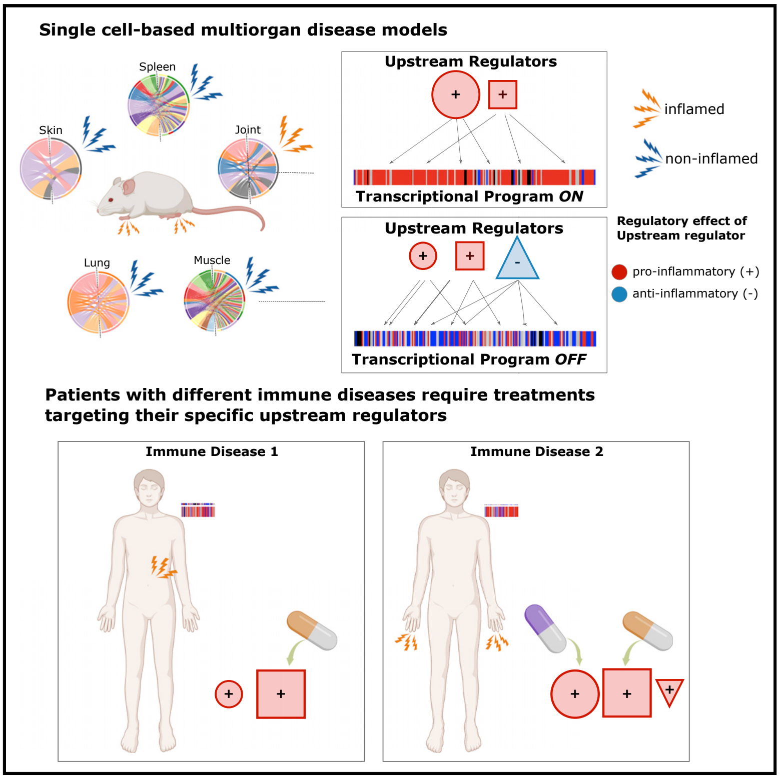

Highlights

- scRNA-seq of mouse arthritis shows organome-, cellulome-, and genome-wide changes
- Those changes are switched on or off by pro- or anti-inflammatory regulators
- A similar, but graded, on/off switch system is found in human immune diseases
- Targeting regulators of this system may be exploited for personalized treatment

Authors

Sandra Lilja, Xinxiu Li, Martin Smelik, ..., Danuta Gawel, Hui Wang, Mikael Benson

Correspondence

mikael.benson@ki.se

In brief

Lilja et al. report that treating immune diseases is complicated by involvement of thousands of genes. Combined analyses of mouse arthritis and human immune diseases show organome-, cellulome-, and genome-wide changes, which are switched on or off by pro- or anti-inflammatory upstream regulators (URs). Targeting URs may contribute to personalized medicine.

Lilja et al., 2023, Cell Reports Medicine *4*, 100956
March 21, 2023

© 2023 The Author(s).

https://doi.org/10.1016/j.xcrm.2023.100956

Cell Reports Medicine

CellPress
OPEN ACCESS

Article

Multi-organ single-cell analysis reveals an on/off switch system with potential for personalized treatment of immunological diseases

Sandra Lilja,[1,11,12] Xinxiu Li,[1,2,12] Martin Smelik,[1,2,12] Eun Jung Lee,[3] Joseph Loscalzo,[4,5] Pratheek Bellur Marthanda,[6] Lang Hu,[7] Mattias Magnusson,[8] Oleg Sysoev,[9] Huan Zhang,[1] Yelin Zhao,[1,2] Christopher Sjöwall,[10] Danuta Gawel,[11,12] Hui Wang,[7,12] and Mikael Benson[1,2,12,13,*]

[1]Department of Pediatrics, Biomedical and Clinical Sciences, Linköping University, 58183 Linköping, Sweden
[2]Medical Digital Twin Research Group, Division of Ear, Nose and Throat Diseases, Department of Clinical Science, Intervention and Technology, Karolinska Institutet, 17165 Stockholm, Sweden
[3]Department of Otorhinolaryngology, Yonsei University Wonju College of Medicine, Wonju, Ganwong 26460, Korea
[4]Department of Medicine, Brigham and Women's Hospital and Harvard Medical School, Boston, MA 02115, USA
[5]Channing Division of Network Medicine, Brigham and Women's Hospital, Harvard Medical School, Boston, MA 02115, USA
[6]Department of Genetics and Genomic Sciences, Icahn School of Medicine at Mount Sinai, 1425 Madison Avenue, New York, NY 10029, USA
[7]Jiangsu Key Laboratory of Immunity and Metabolism, Department of Pathogenic Biology and Immunology, Xuzhou Medical University, Xuzhou, Jiangsu 221000, China
[8]The National Board of Health and Welfare, Socialstyrelsen, 11259 Stockholm, Sweden
[9]Department of Computer and Information Science, Linköping University, 58183 Linköping, Sweden
[10]Biomedical and Clinical Sciences, Division of Inflammation and Infection/Rheumatology, Linköping University, 58183 Linköping, Sweden
[11]Mavatar, Inc, Vasagatan, 11120 Stockholm, Sweden
[12]These authors contributed equally
[13]Lead contact
*Correspondence: mikael.benson@ki.se
https://doi.org/10.1016/j.xcrm.2023.100956

SUMMARY

Prioritization of disease mechanisms, biomarkers, and drug targets in immune-mediated inflammatory diseases (IMIDs) is complicated by altered interactions between thousands of genes. Our multi-organ single-cell RNA sequencing of a mouse IMID model, namely collagen-induced arthritis, shows highly complex and heterogeneous expression changes in all analyzed organs, even though only joints showed signs of inflammation. We organized those into a multi-organ multicellular disease model, which shows predicted molecular interactions within and between organs. That model supports that inflammation is switched on or off by altered balance between pro- and anti-inflammatory upstream regulators (URs) and downstream pathways. Meta-analyses of human IMIDs show a similar, but graded, on/off switch system. This system has the potential to prioritize, diagnose, and treat optimal combinations of URs on the levels of IMIDs, subgroups, and individual patients. That potential is supported by UR analyses in more than 600 sera from patients with systemic lupus erythematosus.

INTRODUCTION

"I never feel completely well." This is a common complaint from patients with immune-mediated inflammatory diseases (IMIDs), despite state-of-the art treatment. This sentiment reflects a general health care problem: according to the US Food and Drug Administration, medication is deemed ineffective in 40%–70% of patients with common diseases.[1] Genome-wide analyses down to the single-cell level indicate that this limited responsiveness depends on both complexity and heterogeneity. Clinical studies have shown that predicting treatment response based on omics data from IMID patients is challenging.[2] Each disease can involve thousands of genes across multiple cell types, which vary between patients with the same diagnosis, and even between the same patient at different time points.[3,4] The clinical manifestations of IMIDs suggest an added layer of heterogeneity, namely, variable organ involvement in the same disease. IMIDs encompass more than 80 diseases, which include rheumatoid arthritis (RA), ulcerative colitis (UC), Crohn disease (CD), psoriasis (PSO), systemic lupus erythematosus (SLE), and many others.[5]

As an example of variable organ involvement, RA can affect not only joints but also the skin and many internal organs, including the kidney, heart, and spleen. Successful pharmacological treatment of such variable organome-wide disease manifestations would ideally require answering questions such as: How many organs are affected? How complex and heterogeneous are the underlying molecular changes? Can those changes be organized into an overriding structure, which permits systematic, and increasingly detailed, analysis? Is there a

© 2023 The Author(s).
This is an open access article under the CC BY license (http://creativecommons.org/licenses/by/4.0/).

hierarchy in the structure? Can that hierarchy be exploited to prioritize diagnostic and therapeutic targets?

These questions have not been investigated on combined organome-, cellulome-, and genome-wide scales. This type of analysis would involve challenges that are close to, or beyond, the limits of current understanding of the architectural principles of disease-associated changes in the multidimensional genome, for example:

1. Characterization of genome-, cellulome-, and organome-wide changes: This can be achieved by single-cell RNA sequencing (scRNA-seq), which allows creation of atlases of all cell types in all organs in healthy mice and humans.[6,7] One reason as to why no similar effort has been made in disease states is that many organs are difficult or impossible to investigate in living human patients. Another reason is that internal organs may not give rise to specific symptoms. As an example, pathogenic mechanisms in lung have been proposed to have a primary role in RA,[8] but clinical and research foci are on joints. It is thus possible that important disease mechanisms, biomarkers, and drug targets are missed. Another problem with focusing on only one organ is that all organs may interact through the hematological, lymphatic, or nervous systems. Thus, they should ideally be studied together, rather than as individual parts.
2. Organization of organome-wide scRNA-seq data: We and others have previously described methods to organize scRNA-seq data from individual organs into multicellular disease models (MCDMs).[9,10] MCDMs are network models that show directed molecular interactions between cell types based on differentially expressed genes (DEGs) in each cell type and their predicted upstream regulators (URs) in other cell types. However, organization of MCDMs on an organome-wide scale is an unresolved challenge.
3. Prioritization of regulatory mechanisms in organome-wide scRNA-seq data: Because MCDMs have not been characterized on an organome-wide scale, their potential interactions have not been systematically investigated, nor is there any form of molecular or cellular hierarchy between organs. However, in a previous study, we found that interactions in an MCDM from one inflamed organ were multidirectional, without any evident hierarchy.[10] This complicated prioritization of diagnostic and therapeutic targets, which emphasizes the need to search for overriding structure to systematically find and prioritize regulatory mechanisms.

Here, we performed multi-organ scRNA-seq of a mouse model of collagen-induced arthritis (CIA) to develop a systems-level strategy to define such structures, which could be validated in human IMIDs (Figure 1). Although disease mechanisms may differ between mouse models and human diseases, we reasoned that overriding structures would be comparable. In summary, we found complex and heterogeneous organome-wide changes in CIA.[11] Those changes could be organized into a multi-organ MCDM (MO-MCDM) in which all organs interacted without evident hierarchy. However, despite the widespread molecular changes across all organs, only joints showed signs of inflammation. This contrast led to the question whether there could be an overriding structure in which complex mechanisms are required not only to activate but also to inhibit inflammation. If so, could that structure be systematically analyzed to prioritize such mechanisms and their URs? Combined analyses of multi-organ data from the mouse model and 10 human IMIDs supported that shared transcriptional programs were switched on or off by variable combinations of URs. Subsequent analyses of IMID patients who did or did not respond to treatment with anti-TNF (tumor necrosis factor), as well as more than 600 blood samples from SLE patients, supported that variable combinations of URs have the potential for personalized diagnostics and therapeutics in IMIDs. We propose prospective clinical studies to examine this potential and have made the data and methods freely available for such studies.

RESULTS

scRNA-seq shows highly diverse cellulome- and genome-wide expression changes in joints and multiple other organs in a mouse model of arthritis

In order to search for systems-level principles to organize and prioritize disease mechanisms on organome-, cellulome-, and genome-wide scales, we performed Seq-Well-based massively parallel scRNA-seq of six DBA1/J mice with CIA and four healthy control mice. Three of the CIA mice developed mild arthritis (per limb arthritis score: 1–3) and three severe arthritis (per limb arthritis score: 4). We first analyzed 10 different organs, namely, joint, blood, draining lymph nodes, lung, thymus, skin, limb muscle, spleen, liver, and kidney, from at least one mouse with severe arthritis and one healthy mouse (Data S1). Despite only joints showing macroscopic signs of disease, we found DEGs between sick and healthy mice in multiple organs. The highest numbers of DEGs were found in muscle, joint, lung, skin, and spleen (Figure S1A). We proceeded to analyze these five organs from all sick and control mice. After filtering and quality control, we recovered 2,230, 814, 4,565, 1,167, and 3,320 cells from joint, lung, muscle, skin, and spleen, respectively (see "method details" in STAR Methods; Data S1). Clustering and cell-type annotation revealed 13 cell types, namely, B cells, dendritic cells, endothelial cells, erythrocytes, fibroblasts, granulocytes, macrophages, monocytes, natural killer (NK) cells, T cells, myocytes, basal III cells, and neutrophils (see "method details" in STAR Methods; Figure 2A; Data S1). Cell-type proportions and DEGs varied greatly between organs (Figures 2B and 2C; Data S1). Of the total number of DEGs identified in macrophages and T cells, which were the only cell types identified in all five organs, 5% and 4%, respectively, intersected over all organs (Data S2). Pathway analysis of the cell types in the different organs resulted in a total of 501 pathways being significantly enriched in at least one cell type, although they were variably upregulated/downregulated in the different organs and cell types in which the direction could be inferred (Figure S2A; Data S3).

The daunting complexity and heterogeneity of the molecular changes across multiple organs and cell types highlighted the overarching questions behind this study: how can disease-associated changes on organome-, cellulome-, and genome-wide scales be organized and prioritized? We reasoned that one

CellPress
OPEN ACCESS

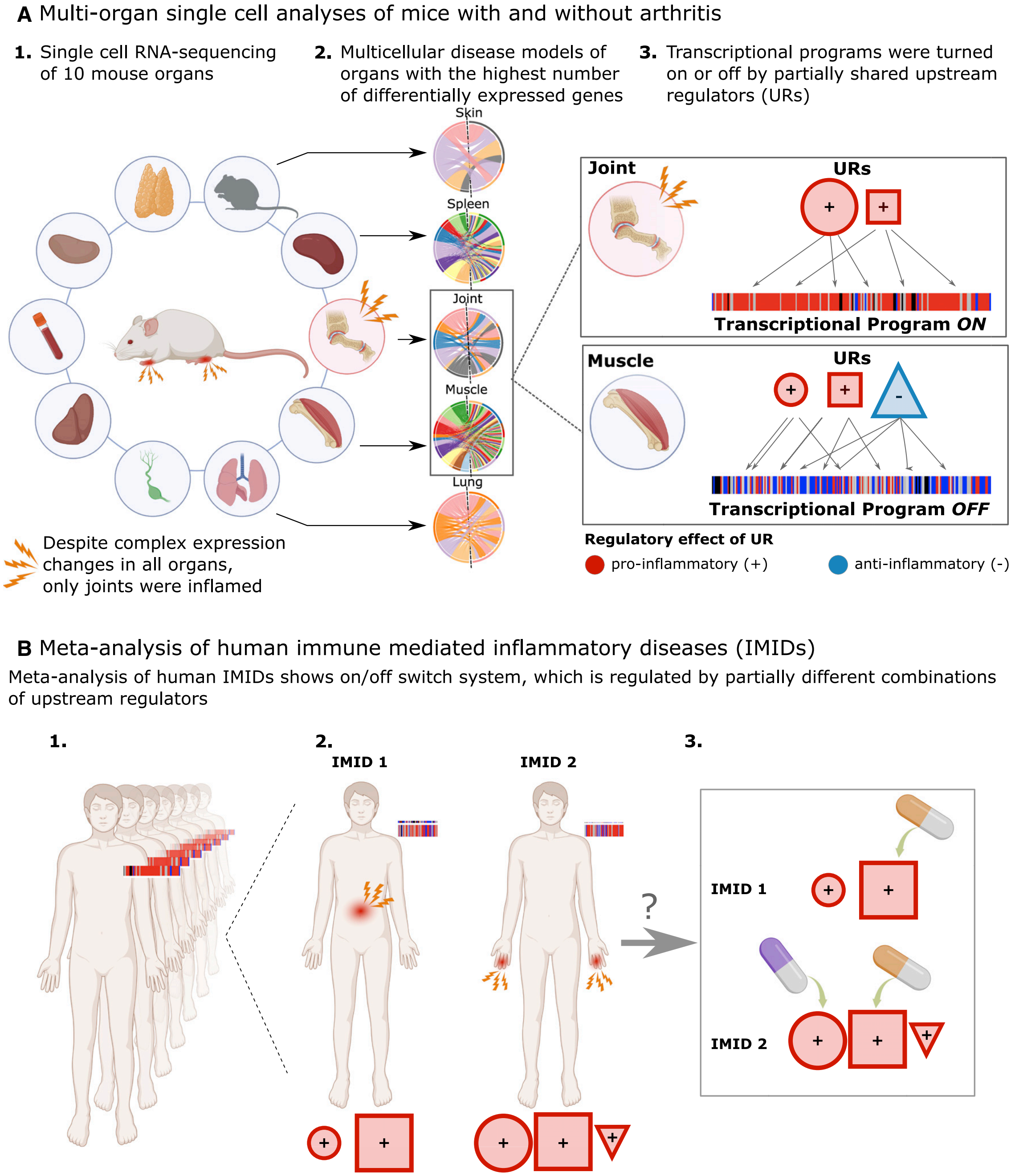

Figure 1. Overview of the study

(A1) Single-cell RNA sequencing (scRNA-seq) of a mouse model of collagen-induced arthritis (CIA) showed thousands of differentially expressed genes (DEGs) across all organs despite only joints showing signs of inflammation. (A2) Multicellular disease models (MCDMs) were constructed based on scRNA-seq data from the organs with the most DEGs. (A3) Transcriptional programs were identified in joints and muscle. These were turned on or off by partially shared combinations of upstream regulators (URs).

(B1) Meta-analysis of multiple immune-mediated inflammatory diseases (IMIDs) showed a similar on/off switch that was (B2) regulated by different UR combinations in different IMIDs. (B3) Those URs have potential for personalized diagnostics and therapeutics, either using single-drug or combinatorial drug treatments.

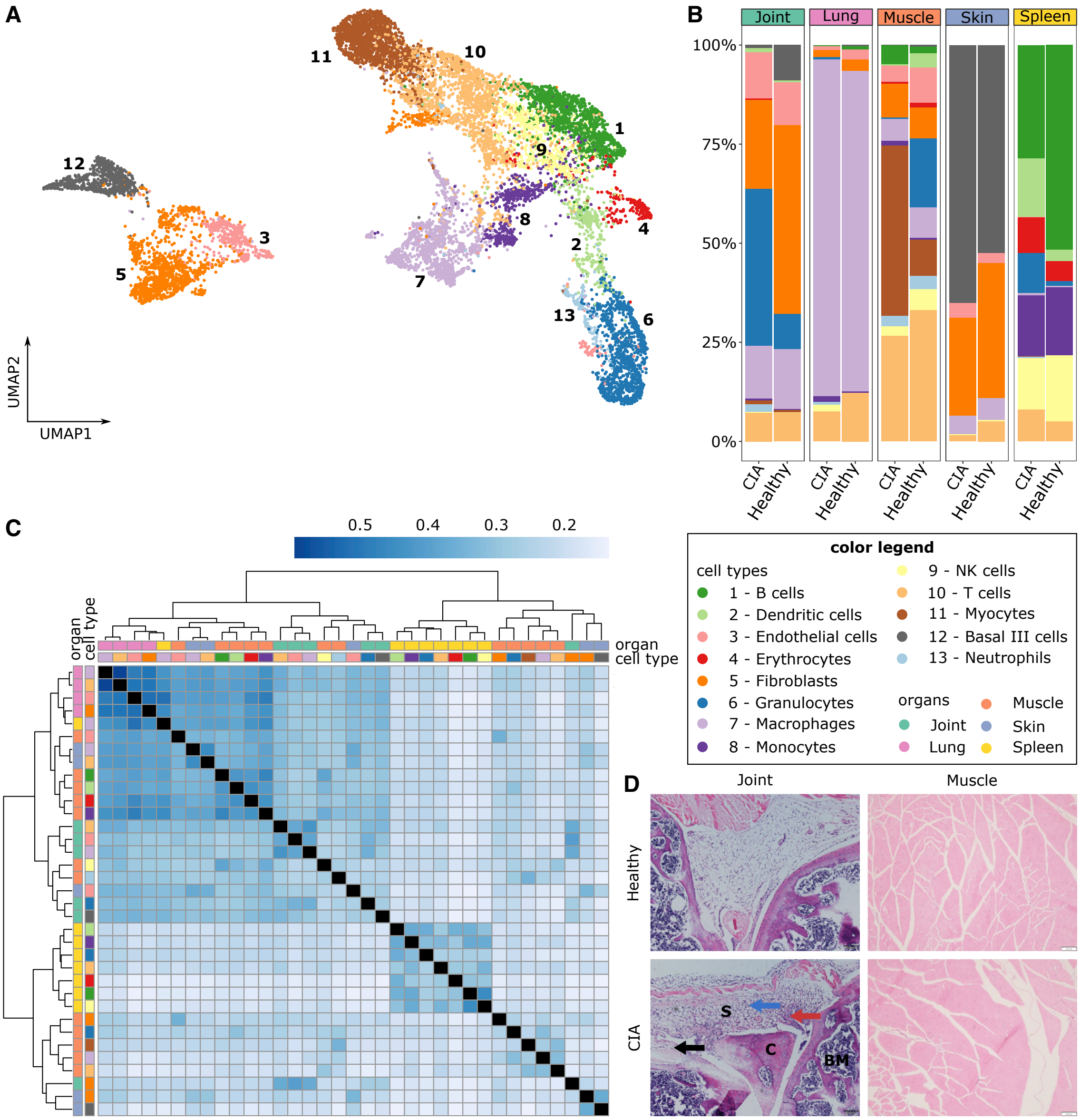

Figure 2. Cellular composition, differential gene expression, and H&E analysis of healthy and CIA mice

(A) UMAP of 12,096 cells from all samples, colored by cell type.

(B) Proportional abundance of cell types per organ and disease state. Healthy mice = 4; CIA mice = 6.

(C) Heatmap presenting the similarity of DEGs. Rows and columns represent different cell types in respective organs, and the color scale corresponds to the Jaccard index.

(D) Representative H&E images of the joint and muscle from control and CIA mice shown at a magnification of 100× (scale bars, 100 μm). Red, black, and blue arrows indicate synovial hyperplasia, bone destruction, and synovial infiltration of inflammatory cells, respectively.

BM, bone marrow; C, cartilage; S, synovial cavity.

straightforward way would be to focus on DEGs in organs with microscopic signs of inflammation, a key endophenotype in CIA.

Histological analysis shows signs of inflammation in joints, but not in other organs

To investigate the inflammation status in multiple organs and cell types, we conducted microscopic analyses of different organs from independent mice with severe CIA (clinical scores >8) and control mice. The results showed inflammation only in joints: significant infiltration of leukocytes in cartilage and synovium, together with bone destruction and synovial hyperplasia (Figures 2D and S1B).

MCDMs show multi-directional networks in each organ without evident hierarchies

The presence of macroscopic and microscopic signs of inflammation only in joints, despite cellulome- and genome-wide

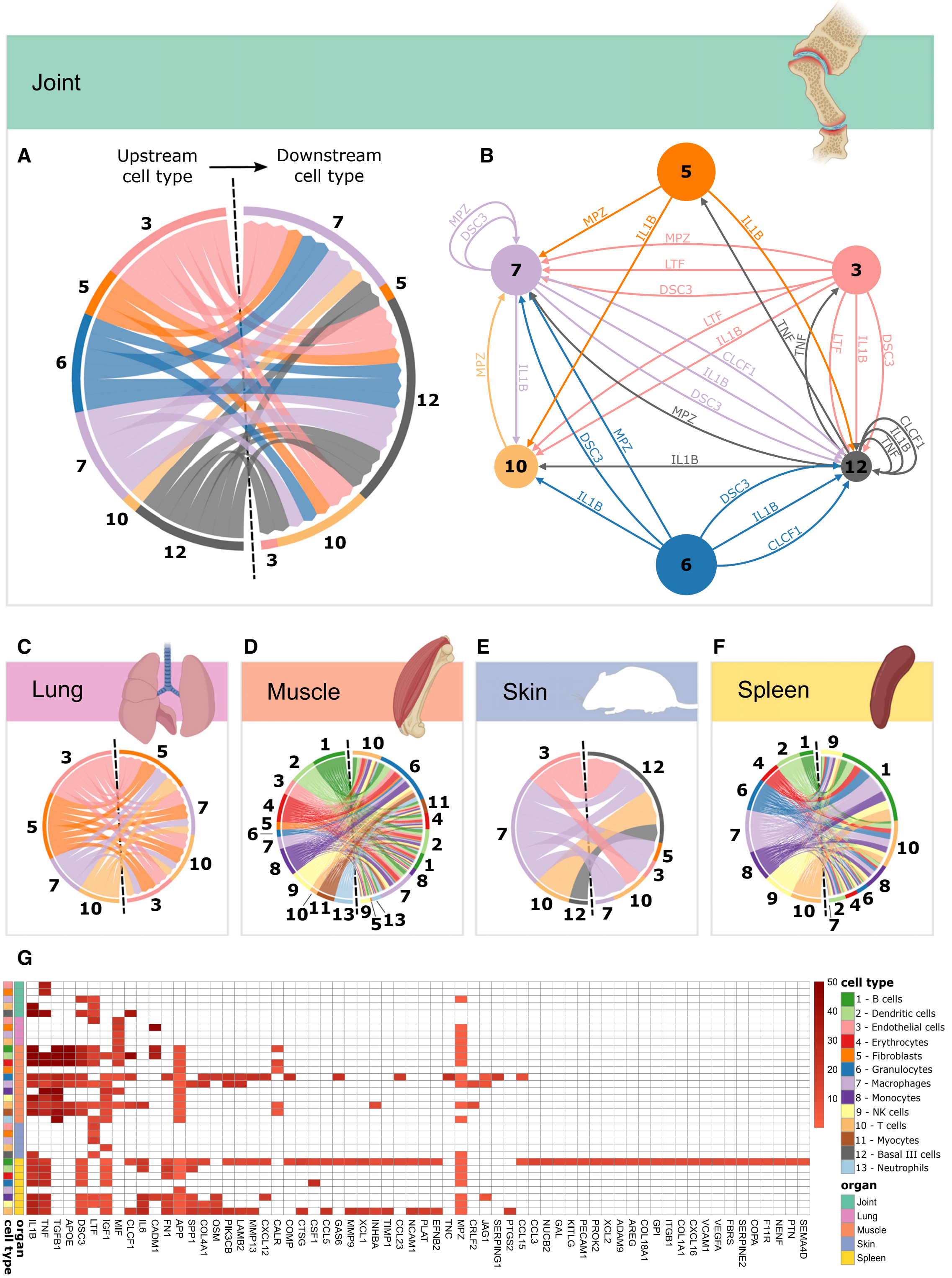

Figure 3. MCDMs of five organs from CIA mice

(A and C–F) Chord charts of predicted molecular interactions between different cell types in (A) joint, (C) lung, (D) muscle, (E) skin, and (F) spleen. Outgoing interactions are shown to the left and ingoing interactions to the right of each chart. Each line represents one interaction between an UR in one cell type and its downstream target genes in another cell type.

(legend continued on next page)

changes in all analyzed organs, suggested an overriding structure underlying the organization and prioritization of DEGs in this system: DEGs in joints activated inflammation, whereas DEGs in other organs suppressed inflammation. If so, this structure would hypothetically act as an on/off switch for inflammation, which could help to prioritize URs and downstream target genes for that switch. To test this hypothesis, we constructed MCDMs of each organ. The MCDMs described predicted molecular interactions between cell types in each organ. The interactions were bioinformatically inferred by linking the DEGs in each cell type with their predicted UR[12] (Data S4). DEGs linked with URs were referred to as downstream targets.[13] Because the interactions were directed, they could potentially be traced to prioritize an UR and cell type with a hierarchically superior role, as well as its downstream target genes in other cell types. We began by analyzing whether the joint MCDM had such a pro-inflammatory UR with an "on" role for the switch. The joint MCDM included URs of known pathogenic importance for both mouse CIA and human RA, such as *Il1b* and *Tnf*.[13] However, the MCDM showed multi-directional interactions mediated by many other URs without evident hierarchy (Figures 3A and 3B). A similar, multi-directional organization was found in MCDMs from lung, spleen, muscle, and skin (Figures 3C–3F).

The complex, heterogeneous, and apparently non-hierarchical changes across multiple organs and cell types led us to attempt to prioritize URs on a multi-organ scale.

Ranking indicates that altered balance between pro-inflammatory and anti-inflammatory URs has an on/off function for inflammation

To prioritize URs, we ranked the URs based on the size of their predicted molecular and cellular effects across all analyzed organs (see "method details" in STAR Methods; Figure 3G). The disease relevance of the ranking system was supported by four of the top-ranking URs in joints, *Il1b*, *Tnf*, *Dsc3*, and *Ltf*, being either therapeutically, functionally, or genetically associated with RA and other IMIDs.[13–16] In further support of the clinical relevance of those URs, the expression of their downstream targets differed significantly between mild and severe arthritis in several cell types in joints: *Tnf* in fibroblasts ($p = 2.00 \times 10^{-3}$), *Il1b* in T cells ($p = 4.14 \times 10^{-5}$), *Ltf* in T cells ($p = 9.51 \times 10^{-5}$) and macrophages ($p = 1.57 \times 10^{-6}$), *Dsc3* in macrophages ($p = 9.9 \times 10^{-7}$), and *Mpz* in macrophages ($p = 1.84 \times 10^{-14}$) (Figure S3; Data S4). Although the increased activity of pro-inflammatory URs in joints was expected, another finding was not: both *Il1b* and/or *Tnf* were also differentially expressed and predicted URs in organs that did not show macroscopic and microscopic signs of inflammation. However, in contrast with joints, the expressions of those URs varied and were counterbalanced by anti-inflammatory URs. For example, in muscle, *Il1b* increased, whereas *Tnf* decreased, and the anti-inflammatory UR *Tgfb* increased (Figure S1C). By contrast, both *Il1b* and *Tnf*, but not *Tgfb*, increased in joints. Thus, the altered balance between pro- and anti-inflammatory URs could act as an on/off switch for inflammation. Such a switch would explain why only joints showed signs of inflammation, despite organome-wide expression changes. Another unexpected finding was that although *Tnf* was downregulated in muscle, its predicted downstream targets in the same organ were activated in monocytes ($p = 6.9 \times 10^{-4}$; Z score = 0.69), and in T cells ($p = 1.02 \times 10^{-3}$; Z score = 1.182). A potential explanation was that the downstream targets were regulated by TNF derived from other organs and transported via the blood. If so, inflammatory mechanisms in different organs are interconnected. We hypothesized that this concept could be developed by searching for molecular interactions between MCDMs, such that a MO-MCDM would be formed.

MO-MCDMs connect inflammatory mechanisms in different organs into a multi-directional network

To investigate systematically if molecular interactions between MCDMs in different organs could be organized into an MO-MCDM, we used the same methods as for individual MCDMs. However, we included only URs predicted to be released into the blood (based on the Human Protein Atlas). We identified 1,966 of such inter-organ interactions, which were mediated by 48 URs (Figure 4; see "method details" in STAR Methods). The resulting MO-MCDM formed a multi-directional network in which all MCDMs were interconnected.

To validate that URs could mediate interconnectivity in the MO-MCDM, we performed protein analyses of high-ranking URs and interacting cytokines in sera from independent CIA mice (Figure S4). Because CIA may variably involve different organs, these analyses were performed at different time points during disease progression.[17]

In support of interconnectivity, all analyzed URs and interacting cytokines were found in sera (Figure S4). TNF increased at early time points, whereas it decreased to normal expression at later stages of the disease. For interleukin (IL)-1β, no systemic changes in protein expression level could be seen at different stages of disease progression. However, for IL-1α, a significant drop in protein expression level was seen at later stages of the disease. IL-6 and interferon γ (IFN-γ), which are known to interact with IL-1β and TNF,[18–21] also showed variable changes in expression level at different time points of disease. Such variations could be consistent with dynamic changes in organ inflammation in CIA.[22] We next analyzed whether the altered balance between pro- and anti-inflammatory URs would be associated with an altered balance between downstream pro- and anti-inflammatory pathways.

Connective pathway analysis supported a graded switch system in CIA

To systematically test whether the altered balance between downstream pro- and anti-inflammatory mechanisms explained why joints, and not muscle, showed signs of inflammation, we

(B) Joint MCDM, showing URs and their predicted, directed interactions. Node size denotes the number of cells of each cell type, and the color of the edges denotes the cellular origin of each interaction.

(G) URs ranked based on their predicted downstream effects. Red spectra indicate the total number of predicted downstream targets for each UR, within each cell type and organ; white indicates that no downstream targets were predicted.

CellPress
OPEN ACCESS

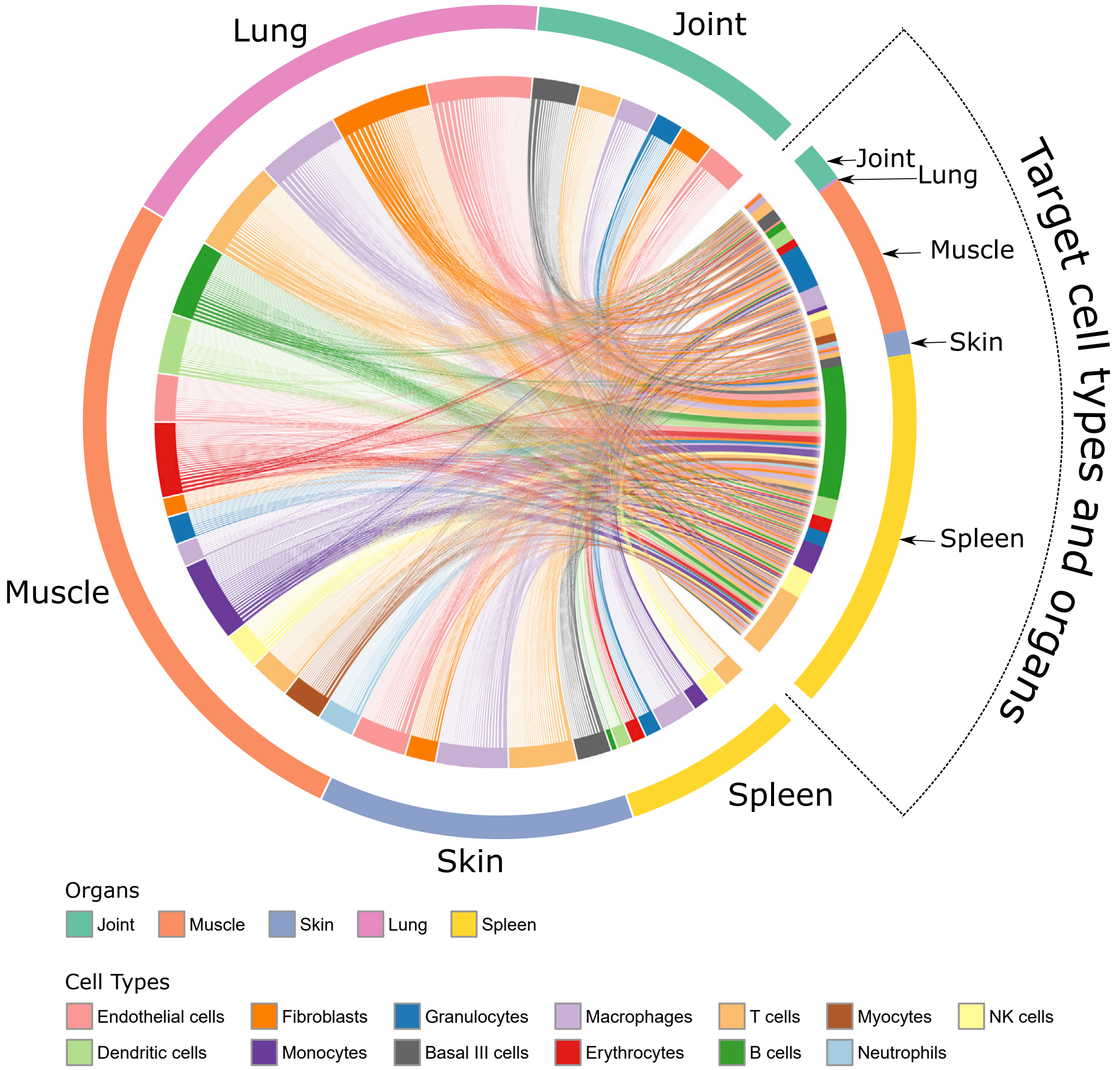

Figure 4. Multi-organ MCDM (MO-MCDM)

The MO-MCDM is presented as a chord chart showing predicted molecular interactions between MCDMs in joints, spleen, lungs, skin, and muscle. Each section in the outer circle represents an organ. Sections in the inner circle represent cell types. Each line represents a ligand secreted by the source cell types (left side of the chart) that were predicted to regulate genes within target cell types (right side of the chart).

performed pathway analysis of all DEGs in different cell types from these two organs. In total, we identified 428 significantly enriched pathways in at least one cell type (Data S3). The large number of pathways complicated systematic testing of our hypothesis. A potential solution was suggested by the fact that 64% of all genes in the 428 pathways were shared by more than one pathway. This led us to hypothesize that a higher-order structure than pathways could be identified, namely, groups of pathways with partially shared genes (henceforth referred to as programs). If such programs were relevant for pathogenesis, they should (1) be enriched in genome-wide association study (GWAS) genes from human RA and (2) differ in activation profiles between joints and muscle. To find such programs, we developed a method called connective pathway analysis. This approach used the 1-Jaccard index as a distance metric for clustering of pathways (pathways that share many genes would then be more proximate to each other than those that do not and, therefore, be closer in the dendrogram; Figure 5A; see "method details" in STAR Methods).

We reasoned that cutting the dendrogram at different levels would provide a systematic approach to prioritizing the programs that differed most in activation profiles between joints and muscle and therefore would be most relevant for an on/off switch. At the highest level of the dendrogram, we found two main CIA-associated programs (CIA_P), CIA_P1 and CIA_P2. Both CIA_P1 and CIA_P2 were enriched for GWAS genes of human RA ($p < 0.006$; Data S5). We also tested whether similar programs would be found using Kyoto Encyclopedia of Genes

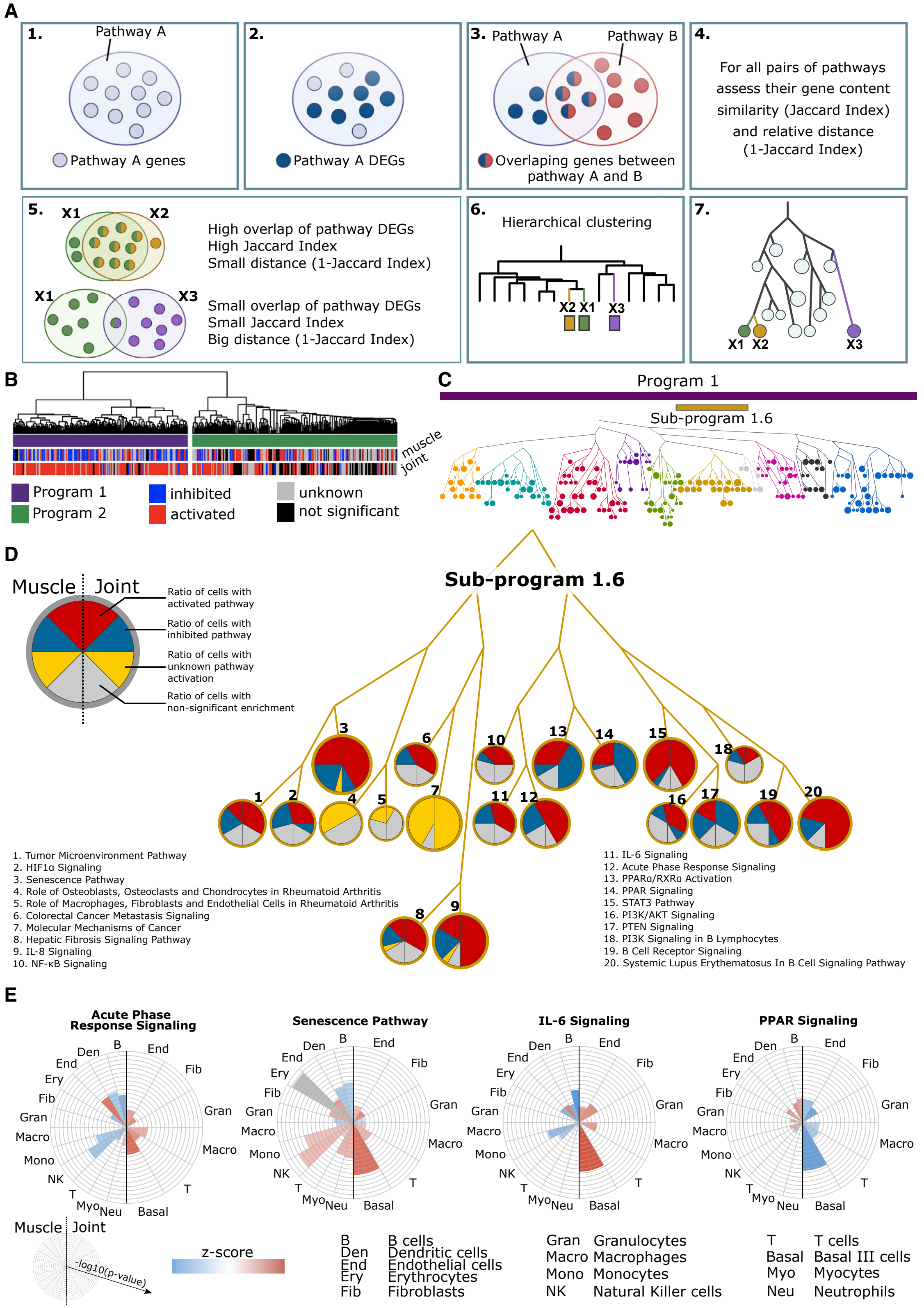

(legend on next page)

CellPress
OPEN ACCESS

and Genomes (KEGG) instead of Ingenuity Pathway Analysis (IPA), and we found significant overlap ($p < 0.0001$; Figure S2B). Analyses of these programs did not support the existence of a discrete on/off switch: both programs included activated pathways in the non-inflamed muscle, compared with healthy control mice. This finding suggested a graded switch system in which the non-inflamed state was an intermediate in a continuous spectrum, in which healthy and inflamed organs were extremes. We next focused on CIA_P1 because pathways were mainly activated in joints and inhibited in muscle (Figure 5B). To facilitate the identification of pathways that had the most opposing activation directions in CIA_P1 (i.e., being activated in one organ and inhibited in the other or being significantly enriched in one organ and not significant in the other; see "method details" in STAR Methods), we cut the dendrogram into 10 subprograms (CIA_SPs) (Figure 5C). Of these, CIA_SP1.3, CIA_SP_1.1, and CIA_SP1.6 showed the highest percentages of pathways with opposing activation directions (79%, 61%, and 60%, respectively; Data S3). The highest GWAS enrichment was found in CIA_SP1.6 (Data S5). Further analysis of CIA_SP1.6 showed that 75% of its pathways were related to human RA (Data S3). CIA_SP1.6 contained pro-inflammatory pathways such as "Acute Phase Response Signaling" and "IL-6 signaling," as well as anti-inflammatory pathways such as "PPAR signaling" (Figure 5D). The pro-inflammatory pathways were mainly activated in joint and inhibited in muscle, whereas the anti-inflammatory pathways showed the opposite pattern (Figure 5E). *Il1b* and *Tnf* were predicted URs of CIA_SP1.6 in both joint and muscle (Data S6). However, in muscle, the downregulation of the pro-inflammatory URs *Tnf* and *Apoe*[23] and upregulation of the anti-inflammatory *Tgfb1* (Figure S1C) could explain why no signs of inflammation were found. By contrast, increased expression of *Il1b* in muscle was consistent with partial activation of some pro-inflammatory pathways. For example, the "Senescence Pathway" showed mixed activation or inhibition in different cell types in both joint and muscle (Figure 5D). This pathway has previously been implicated in IL-1- and TNF-induced tissue damage in RA.[24,25] The mixed activation pattern of the "Senescence Pathway" in joints and muscle is thus consistent with mixed activation of IL-1 and TNF in these two organs. Taken together, these findings support a graded, rather than discrete, on/off system. We next examined whether such a system could be translated to human IMIDs.

Meta-analysis of human IMIDs supported a graded switch system

To test the disease relevance of the graded switch system, we performed meta-analysis of 10 different IMIDs (Data S1): RA, UC, CD, PSO, Sjögren's syndrome (SS), systemic sclerosis (SSc), atopic dermatitis (AD), juvenile myositis (JM), "at risk for" type 1 diabetes (T1D), as well as SLE. The SLE datasets included discoid lupus erythematosus (DLE), subacute cutaneous lupus erythematosus (SCLE), and lupus nephritis (LN). The meta-analysis was based on 32 bulk profiling datasets from human organ biopsies. The IMID biopsies were taken from inflamed and/or non-inflamed sites and compared with biopsies from healthy controls. Meta-analysis of DEGs from each IMID showed highly complex changes in inflamed and non-inflamed sites with 647 pathways that differed significantly compared with controls (Data S3). Similar to CIA, connective pathway analysis revealed two IMID-associated programs (IMID_P): IMID_P1 and IMID_P2 (Figure 6A).

The disease relevance of both IMID_P1 and IMID_P2 was supported by significant enrichment for GWAS genes (Data S5). However, when we compared the pathway overlap between IMID_P1 and IMID_P2 with the corresponding programs from each individual IMID, we mainly found significant overlaps within IMID_P1 (Figure 7A; see "method details" in STAR Methods). This observation indicated that IMID_P1 contained pathways that were shared across IMIDs, whereas IMID_P2 had more disease-specific pathways. In IMID_P1, 32% of pathways were activated in inflamed organ sites and inhibited, or not significant, in non-inflamed sites. The corresponding figure for IMID_P2 was 21%. Both IMID_P1 and IMID_P2 also included many activated pathways in non-inflamed sites. In agreement with a graded switch system across a spectrum between health and an inflamed phenotype, this more graduated response could predict an increased risk of a gradual shift in the balance between pro- and anti-inflammatory pathways toward an inflamed phenotype.

We next cut the dendrograms of both programs into subprograms (IMID_SPs) in order to prioritize the subprogram that had the most pronounced on/off pattern and GWAS enrichment. This analysis led to prioritization of IMID_SP1.6, which had 49% of pathways with opposing patterns between inflamed and non-inflamed organ sites, and GWAS enrichment in 72% of the IMID datasets, with median (range) odds ratio (OR) among the significant = 5.34 (~3.54–8.94) (Figures 6, 7A, and 7B; Data S3 and

Figure 5. Connective pathway analysis to systematically define and prioritize transcriptional programs in joint and muscle from the CIA mouse model

(A) Outline of connective pathway analysis: (1) identification of genes that belong to a pathway; (2) mapping DEGs on that pathway; (3) pairwise comparison of pathway-associated DEGs; (4) assessment of pathway-associated DEGs overlap (Jaccard Index); (5) examples of two extreme situations, pathways X1 (green) and X2 (orange) have a high overlap of DEGs but not X1 (green) and X3 (purple); (6) hierarchical clustering using the 1-Jaccard index as distance matrix; and (7) dendrogram transformation into a tree-like structure.

(B) Connective pathway analysis identified two main programs, CIA_P1 (purple) and CIA_P2 (green). Each pathway was labeled as "activated" (red), "inhibited" (blue), "unknown activation" (gray), or "not significant" (black).

(C) Tree-like representation of CIA_P1 with subprograms indicated with different node colors. Color bars indicate main program CIA_P1 (purple) and subprogram CIA_SP1.6 (ochre). Node size represents the total number of cell types in which the pathway is significantly enriched.

(D) Detail of CIA_SP1.6. Each node represents a pathway, and pie charts within nodes represent ratios of cell types in muscle and in joint (left and right part of the pie chart, respectively) for which the pathway was significantly enriched. Colors represent pathway activation profile.

(E) Detail of selected pathways from CIA_SP1.6. Left (right) part of polar charts presents all cells in muscle (joint). Each pie sector represents one cell type. The degree to which the sector is filled with color represents enrichment $-\log10$(p value), whereas the color shows pathway activation (blue for inhibition, red for activation, and gray for unknown activation).

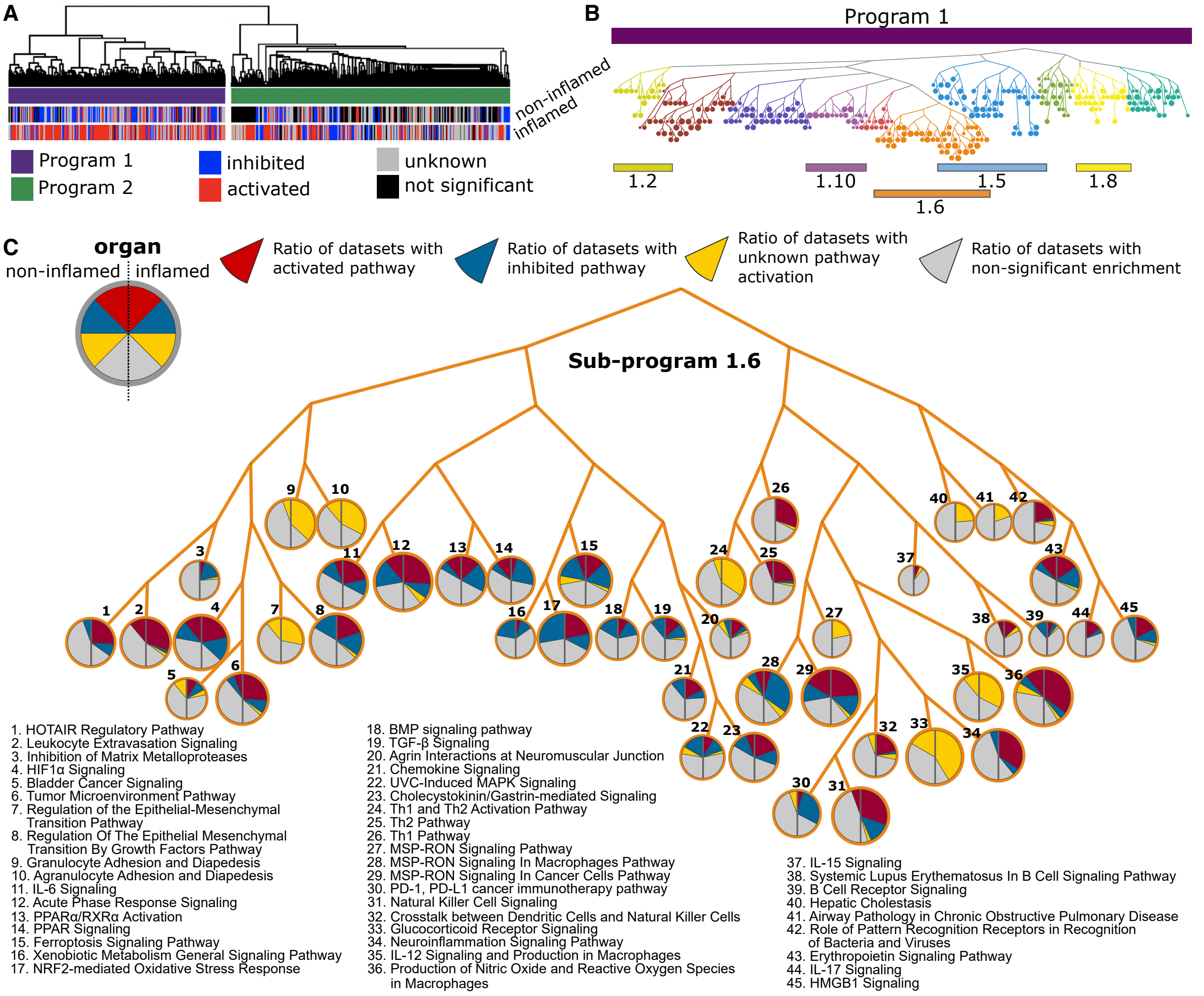

Figure 6. Transcriptional programs in inflamed and non-inflamed organs from IMIDs

(A) Hierarchical clustering of all significant pathways in inflamed and non-inflamed organs from IMIDs identified two programs, IMID_P1 (purple) and IMID_P2 (green). Each pathway was labeled as "activated" (red), "inhibited" (blue), "unknown" (gray), or not significant (black).

(B) Tree-like representation of subprograms in IMID_P1. The subprograms are indicated by different colors and numbers, with each node representing a pathway.

(C) Detail of IMID_SP1.6. Each node represents one pathway. Pie charts within nodes represent ratios of datasets in non-inflamed and inflamed organ groups (left and right part of the pie chart, respectively) for which a particular pathway was predicted to be "activated," "inhibited," "unknown" direction, and "not significant."

S5). We also found that IMID_SP1.6 was shared across inflamed organ sites in all IMIDs but PSO (Figure 7A).

The top-ranking pathways in IMID_SP1.6, in inflamed sites, were "Acute phase response signaling," "B cell receptor signaling," "Chemokine signaling," and "IL-6 signaling." By contrast, anti-inflammatory pathways, such as "PPAR signaling" and "PPARa/RXRa Activation,"[26–28] were inhibited (Figure 6C). However, IMID_SP1.6 also included activated pro-inflammatory pathways in non-inflamed organ sites: "Leukocyte extravasation," "Natural Killer Signaling," and "MS-RON Signaling," all of which can contribute to chronic inflammation, and thereby a switch from off to on.[29]

Analysis of the URs of IMID_SP1.6 agreed with the graded switch system being regulated by variable combinations of pro- and anti-inflammatory URs. This could have important basic and clinical implications, namely, that the stepwise characterization of programs and subprograms, as described above, could help to prioritize, diagnose, and treat optimal combinations of URs on the levels of IMIDs, subgroups, and individual patients.

Combinatorial regulation of the graded switch system has diagnostic and therapeutic implications

For all IMIDs combined, we found a total of 389 predicted URs (Data S6). Specifically, for each disease we found a median

CellPress
OPEN ACCESS

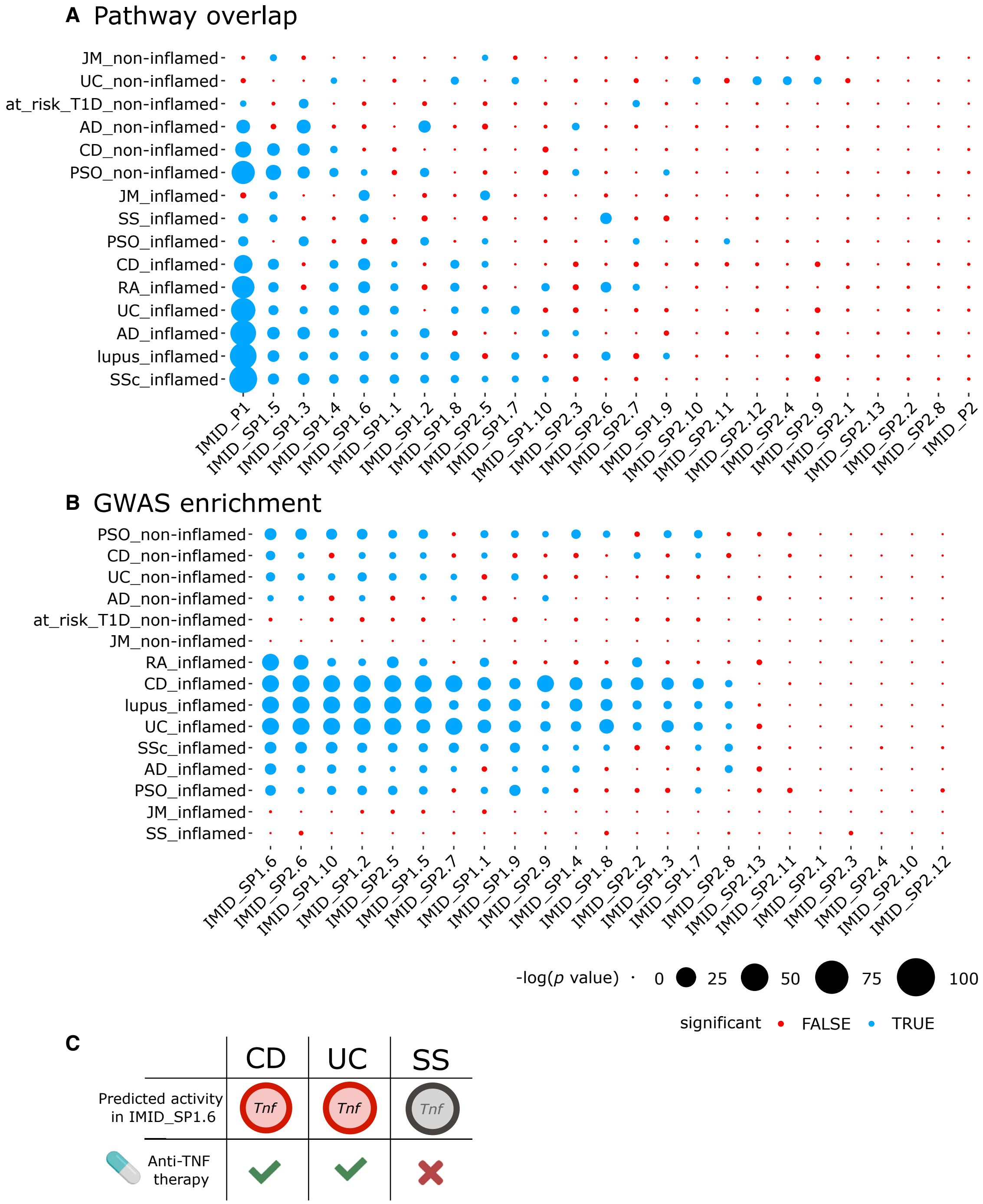

Figure 7. Relevance of programs/subprograms for different human IMIDs

(A) Pathway overlaps between programs/subprograms from all analyzed IMIDs and each individual IMID. Significant overlap is denoted with blue color, and non-significant with red (see "method details" in STAR Methods). Node size corresponds to the −log10(p value). The programs and subprograms (rows) were ordered by increasing number of associated IMIDs.

(B) GWAS enrichment of subprograms in each IMID.

(C) The predicted activity of TNF corresponds to the known clinical effects of anti-TNF treatment. Red color corresponds to significant predicted activity, whereas gray denotes non-significant activity. Green checkmarks and red X denote whether the anti-TNF treatment is clinically effective or not, respectively.

(range) of 79 (0–218) URs, of which only 8 were shared by all IMIDs (except SS), namely, AR, ER-β, Fas, IFN-γ, IL-1α, IL-1β, TLR3, and TNF. In agreement with the graded switch system depending on altered balance between pro- and anti-inflammatory URs, Fas, IFN-γ, IL-1α, IL-1β, TLR-3, and TNF are mainly pro-inflammatory, whereas AR and ES-β are anti-inflammatory.[30–35] Unexpectedly, however, the predicted effects of these URs, based on *Z* score, contrasted with their measured fold changes (FCs; Figures S5A and 5B). For example, TNF was predicted to be activated in 25 datasets, whereas it was differentially expressed in only 13 datasets. This difference could be explained by URs, other than TNF, having redundant effects on the same downstream target genes. Thus, one or more URs could have "backup" functions if another UR, like TNF, was therapeutically inhibited. We tested this hypothesis in IMID patients treated with anti-TNF.

Different combinations of URs with redundant functions may explain variable response to anti-TNF treatment

TNF was predicted to be a top-ranking UR of IMID_SP1.6 in inflamed states of both UC and CD, but not in SS (Figures S5A and 5B; Data S6). These predictions agree with the clinical experience that anti-TNF treatment is effective in the two former diseases, but not in the latter (Figure 7C). This led us to examine the effects of anti-TNF treatment on subprograms in UC and CD. The pathways analyses of DEGs after treatment between CD patients who responded to anti-TNF (GEO: GSE52746, 10 treated anti-TNF responders versus 7 untreated patients) showed significant enrichment of anti-TNF-targeted pathways among IMID_P1 and subprograms IMID_SP1.6, IMID_SP1.9, and IMID_SP2.5 (false discovery rate [FDR], $<4.58 \times 10^{-2}$) (Data S6). The corresponding analyses of DEGs from patients with UC (GEO: GSE92415; 29 treated anti-TNF responders versus 32 untreated patients) showed significant enrichment of anti-TNF-targeted pathways in the subprograms IMID_SP1.2 and IMID_SP2.7 (FDR, $<1.99 \times 10^{-2}$). The enriched subprograms are henceforth referred to as affected subprograms and the others as non-affected. The relevance of the affected subprograms was supported by TNF being predicted to regulate all of them in inflamed organs (Data S6). For subprogram IMID_SP1.6, which we in this study identified as highly relevant for IMIDs, six of seven pathways (where a $|Z \text{ score}| > 0$ could be inferred) showed the opposite direction of activation as a result of treatment response compared with how they were affected by the disease (Figure S5C), indicating effective treatment response.

We next tested the hypothesis that the non-affected subprograms could be explained by URs whose downstream targets overlapped with TNF, having redundant, "backup," functions. In UC, we found 14 URs predicted to co-regulate non-affected subprograms. For example, NR4A2 was predicted to co-regulate 8 out of 16 non-affected subprograms (Figure S6A). In support of this prediction, *NR4A2* was significantly upregulated in inflamed samples of UC (FDR, 2.07×10^{-4}; logFC = 1.31). By contrast, affected subprograms were not predicted to be regulated by NR4A2.[31]

We further tested the potential of other URs to take over the effect of TNF in UC patients who did not respond to anti-TNF treatment. The pathways enriched among the DEGs of responders, respectively non-responders versus controls, showed similar main and subprogram associations (Figure S6B). We identified 92 predicted URs for the DEGs of non-responders versus controls. By predicting the potential for each alternative UR predicted for non-responders to take over the downstream effect of TNF (see "method details" in STAR Methods), we identified TLR6 as a potential UR to take over the effect of TNF among the non-responders after treatment.

To verify that the downstream genes of TLR6 and TNF followed the expected FCs before and after treatment, we additionally analyzed the DEGs for responders and non-responders after treatment versus controls. As expected, we found a tendency for a higher |FC| of the TLR6 downstream genes among the non-responders compared with the responders, before and after treatment (Figure S6C). However, only CXCL8 showed a significant difference between responders and non-responders after treatment ($p = 3.62 \times 10^{-2}$) (Figure S6C). Furthermore, the TNF downstream genes showed smaller differences compared with control for the treated responders, compared with any of the untreated groups or treated non-responders (Figure S6D).

Because UC and CD primarily affect the intestine, we next analyzed whether different UR combinations were associated with variable organ involvement in another IMID that often shows multi-organ involvement, namely, SLE. The clinical relevance lies in that this could indicate the need for diagnostic and therapeutic targeting of different URs in patients with different forms of organ involvement.

Different combinations of UR proteins in sera were associated with different subtypes of SLE, as well as with disease severity

We analyzed 18 predicted UR proteins of IMID_SP1.6 in sera from two clinical visits of 304 patients who had been prospectively seen by the same rheumatologist (C.S.) at the tertiary referral unit, Linköping University Hospital, according to standardized criteria.[36] The American College of Rheumatology (ACR) criteria were used to define disease phenotype.[37] We constructed regression models to estimate relationships of UR proteins with ACR criteria, as well as measures of disease activity and organ damage (SLE disease activity index-2000 [SLEDAI] and Systemic Lupus International Collaborating Clinics/ACR index [SDI], respectively) (Figure S7). Clinical variables, including results from physical examinations, laboratory values, age, gender, and duration of the disease, were collected and used in all our models. We also included the treatment information in regression models that predicted SLEDAI and SDI. We did not use treatment information in models predicting ACR criteria, because the treatment is dependent on the patient phenotype, e.g., patients with LN are widely treated by mycophenolate mofetil (MMF). We showed that different UR proteins were associated with different ACR phenotypes (Data S1). For example, cases classified with ACR-1 (malar rash) were positively associated with CD40-L and negatively associated with Fas. Cases classified with ACR-7 (renal disorder/LN) were positively associated with FAS but negatively associated with hepatocyte growth factor (HGF). TNF was positively associated with ACR-9 (hematological disorder) and ACR-10 (immunological disorder). By contrast with ACR-7 and ACR-9 (hematological disorder), TNF

and HGF were significantly associated with ACR-6 (serositis), TNF negatively and HGF positively.

Next, we examined whether different combinations of potential URs were associated with disease activity in patients with (n = 80) and without (n = 224) LN. The regression models showed that different proteins were associated with the disease activity in the two groups (Data S1). IL-1α, IL-4, Fas, and oncostatin M (OSM) were associated with SLEDAI in patients with LN, whereas TNF, HGF, and CD40 were associated with SLEDAI in patients without LN. In agreement with our hypothesis, the URs that were negatively correlated with SLEDAI may have anti-inflammatory roles. However, testing whether altered expression of these URs inhibit or activate inflammation is complicated by their context-dependent pleiotropic roles. For example, OSM has an anti-inflammatory role on synovial cells: it reduces IL-1 and TNF expression, but it has a pro-inflammatory role on endothelial cells by inducing leukocyte recruitment and IL-6 production from endothelial cells.[38,39] A similar pleiotropy has been described for HGF and IL-4. Focusing on organ damage (Data S1), we found that TNF, IL-27, OSM, and TGF-β1 were associated with SDI in patients with LN. In contrast, IL-6, IL-1α, IL-2, HGF, and CD40-L were associated with SDI in patients without LN.

DISCUSSION

The main problem behind this study is that many patients with IMIDs do not respond adequately to treatment.[1] An important reason for this inadequate response is the daunting complexity and heterogeneity of the molecular changes in these diseases. scRNA-seq studies of IMIDs have shown altered expression of thousands of genes across multiple cell types in individual organs with phenotypic signs of disease.[40–42] Despite detailed information about those changes, translation of the data to personalized medicine has proven difficult. Thus, there is a wide gap between the complexity of disease-associated changes and health care today. Bridging that gap will likely involve great challenges for researchers and clinicians.

Variable multi-organ involvement in each IMID adds to the complexity and heterogeneity. This indicates the need for characterization, organization, and prioritization of molecular changes on organome-, cellulome-, and genome-wide scales. Our multi-organ scRNA-seq analyses of a mouse model of an IMID, namely, CIA, showed extensive changes on all those scales. Although those changes could be organized into an MO-MCDM, this analytical approach showed no evident molecular or cellular hierarchy that would allow prioritization of molecular changes. An unexpected finding led us to a potential solution: despite the organome-wide changes, only joints showed signs of disease. That contrast led us to hypothesize that the expression changes were organized into an overarching structure designed to switch inflammation on or off. We developed an analytical strategy that supported such an on/off switch and showed that it depended on altered balance between pro- and anti-inflammatory URs. Such a switch has been previously suggested in inflammatory responses and validated by functional studies of individual genes and cell types.[40–42] Given the complexity and heterogeneity of the organome-, cellulome-, and genome-wide changes, ranking of URs is crucial for understanding and prioritization of disease mechanisms. Our strategy provided a solution for systematic characterization and prioritization of URs, as well as their downstream target genes. We found that URs could be ranked based on the size of their effects on the downstream genes. In support of clinical relevance, the top-ranking URs included known therapeutic targets in IMIDs, including IL-1 and TNF. The downstream target genes could be organized into two main programs, and their subprograms, which permitted increasingly detailed analyses of pathways. In support of disease relevance, both programs were significantly enriched for genes identified by GWAS of human RA. However, large molecular changes in non-inflamed organs, including partially activated pathways, did not support a discrete on/off switch, in which non-inflamed organs corresponded to an "off" state. Instead, the activated pathways could increase the risk of a switch to an "on" state. This observation is consistent with a graded on/off switch, in which non-inflamed organs are intermediates on a spectrum, where healthy and inflamed organs represent extremes. Such an intermediate "risk" state could explain an important characteristic of both CIA and human IMIDs, namely, variable organ involvement during disease progression. A graded on/off switch has been previously described in model organisms and proposed to be generally applicable to biological systems. The relevance of molecular gradients in disease is supported by previous findings that variable expression or dysregulation of interconnected genes will define whether an organ is affected by disease.[43]

The translational relevance of the strategy was supported by meta-analyses of human IMIDs. These showed a similar organization as in CIA, with URs, programs, and subprograms that agreed with a graded on/off switch. High-ranking URs included known drug targets such as IL-1 and TNF. However, except for a core group of URs, these varied across diseases and organs. For example, TNF was a predicted UR in IBD, but not in SS, which agrees with the clinical experience that anti-TNF treatment is effective in the former, but not in the latter. Clinical implications may be that characterization and ranking of URs will be needed for successful treatment, on the levels of IMIDs, subgroups, or even individuals. Those implications were supported by our analyses of the effects of anti-TNF treatment in patients with IBD who did or did not respond to that treatment. We found that lack of response could be explained by overlapping downstream effects of the URs, such that the effect of inhibiting one UR could be diminished by one or more other, functionally redundant, "backup" URs. Prospective clinical studies are warranted to test whether predictive classifiers for treatment response can be developed based on high-ranking URs. To examine whether UR combinations would vary in patients with different forms of organ involvement, we focused on an IMID with highly variable multi-organ manifestations, SLE. Analyses of predicted UR proteins in more than 600 sera from SLE patients did show that different combinations of UR proteins correlated with different forms of organ involvement. Furthermore, different combinations of URs were associated with disease severity in SLE patients with and without renal involvement. A clinical implication may be that different URs should be targeted in these two subgroups. Moreover, some shared URs showed opposing associations.

Thus, targeting of such URs could have curative or aggravating effects in different subgroups of patients with the same disease. Interestingly, we found that HGF was positively associated with the damage index but negatively associated with organ involvement in SLE patients without renal involvement. This supports our message that a systems-level strategy for prioritization of URs on an organome-wide scale is important. Similar to CIA, several subprograms in non-inflamed organs included activated pro-inflammatory pathways. The pathogenic relevance of those subprograms was supported by enrichment of genes identified by GWAS. In agreement with a graded switch system, these subprograms could enhance risk of altered balance between pro- and anti-inflammatory pathways, resulting in an inflammatory phenotype or amplification of that phenotype. A potential clinical implication is the development of combinatorial diagnostic and therapeutic targeting of such URs during disease remission to prevent a gradual switch to active disease.[3,43] We propose that our strategy to organize and prioritize disease-associated changes on organome-, cellulome-, and genome-wide scales has significant potential for future studies aimed at personalized combinatorial diagnostics and therapeutics. We have made the methods and data freely available for such studies.

Limitations of the study

The analytical strategy was derived from multi-organ analyses of a mouse model of CIA, which may not be representative of human disease. Moreover, the CIA model includes use of an adjuvant to enhance the inflammatory response, which could induce pro-inflammatory pathways in tissues other than joints. The construction of MCDMs and the predicted effects of combinations of URs on downstream genes were based on previously described or predicted protein interactions, which may be confounded by knowledge bias. Although the analyses of the CIA mouse model were based on scRNA-seq, the analyses of human IMIDs were performed on bulk RNA-seq. From a translational perspective, future experimental and clinical studies are warranted to examine the diagnostic and therapeutic implications of the study, in particular the effects of URs on downstream genes.

STAR★METHODS

Detailed methods are provided in the online version of this paper and include the following:

- KEY RESOURCES TABLE
- RESOURCE AVAILABILITY
  - Lead contact
  - Materials availability
  - Data and code availability
- EXPERIMENTAL MODEL AND SUBJECT DETAILS
  - Mouse model of arthritis
  - Human data
- METHOD DETAILS
  - Study design
  - CIA mouse model generation
  - Histological analysis
  - Sample cryopreservation
  - Sample thawing
  - Single cell suspension
  - scRNA-seq wet-lab protocol
  - Cytokine analyses in peripheral blood
  - The analysis of the UR protein expressions in SLE sera
  - Measurement of the UR proteins in SLE sera
- QUANTIFICATION AND STATISTICAL ANALYSIS
  - Organ prioritization
  - scRNA-seq data processing
  - Clustering and cell type identification
  - Data normalization and DEG analysis
  - Cell type identification using marker genes
  - Cell-cell interaction analysis
  - MCDM construction and UR prioritization
  - Pathway enrichment analysis
  - The association between UR-target expression and arthritis score
  - Connective pathway analysis
  - Prediction of URs regulating specific programs/subprograms in connective pathway analysis
  - Differential expression analysis of expression profiling data from immune-mediated inflammatory diseases
  - Genome-wide association study (GWAS) enrichment analyses within programs and subprograms
  - Prioritization of URs in IMIDs
  - Overlap between programs from all analyzed IMIDs and individual IMIDs
  - Treatment effect on IMIDs

SUPPLEMENTAL INFORMATION

Supplemental information can be found online at https://doi.org/10.1016/j.xcrm.2023.100956.

ACKNOWLEDGMENTS

We acknowledge the Affinity Proteomics-Stockholm Unit at SciLifeLab Sweden for support with the Luminex data analysis. This work was supported by the National Institutes of Health (grants HG007690 and HL155107 to J.L.), American Heart Association (grants 957729 and D007382 to J.L.), EU Horizon Health 2021 (grant 101057619 to J.L.), Ruggeri Network Medicine Research Fund (J.L.), Swedish Cancer Society (grant CAN 2017/411 to M.B.), Cocozza Foundation (M.B.), The Research Council of Norway, under the frame of Nordforsk (90825 to M.B.), The Basic Science Research Program through the National Research Foundation of Korea (NRF) grant funded by the Korea government (Ministry of Science and information communication technology (ICT); NRF-2021R1A2C1010082 to E.J.L.), a faculty research grant of Yonsei University Wonju College of Medicine (2022-52-0054 to E.J.L.), Swedish Research Council VR grant 2019-01165 (to H.Z.), National Natural Science Foundation of China (82171791 to H.W.), Swedish Rheumatism Association (R-939149 to C.S.), Region Östergötland (ALF grant RÖ-960604 to C.S.), Ulla and Roland Gustafsson Foundation (2021) (C.S.), and King Gustaf V's 80-Year Anniversary Foundation (FAI-2020-0663 to C.S.).

AUTHOR CONTRIBUTIONS

Conceptualization, S.L., X.L., M.S., and M.B.; methodology, S.L., X.L., M.S., E.J.L., P.B.M., L.H., M.M., H.Z., Y.Z., C.S., D.G., and H.W.; writing – original draft, S.L., X.L., M.S., Y.Z., D.G., and M.B.; writing – review & editing, S.L., X.L., M.S., Y.Z., J.L., D.G., and M.B.; visualization, S.L., X.L., M.S., and D.G.; supervision, D.G., H.W., and M.B.; project administration, S.L., X.L., and M.S.

CellPress
OPEN ACCESS

DECLARATION OF INTERESTS

M.B. is scientific founder of Mavatar, Inc. J.L. is co-scientific founder of Scipher Medicine, Inc.

INCLUSION AND DIVERSITY

We support inclusive, diverse, and equitable conduct of research.

Received: August 17, 2022
Revised: September 30, 2022
Accepted: February 3, 2023
Published: February 28, 2023

REFERENCES

1. FDA (2013, October). Paving the Way for Personalized Medicine: FDA's Role in a New Era of Medical Product Development.
2. Humby, F., Durez, P., Buch, M.H., Lewis, M.J., Rizvi, H., Rivellese, F., Nerviani, A., Giorli, G., Mahto, A., Montecucco, C., et al. (2021). Rituximab versus tocilizumab in anti-TNF inadequate responder patients with rheumatoid arthritis (R4RA): 16-week outcomes of a stratified, biopsy-driven, multicentre, open-label, phase 4 randomised controlled trial. Lancet *397*, 305–317. https://doi.org/10.1016/S0140-6736(20)32341-2.
3. Li, X., Lee, E.J., Lilja, S., Loscalzo, J., Schäfer, S., Smelik, M., Strobl, M.R., Sysoev, O., Wang, H., Zhang, H., et al. (2022). A dynamic single cell-based framework for digital twins to prioritize disease genes and drug targets. Genome Med. *14*, 48. https://doi.org/10.1186/s13073-022-01048-4.
4. Zhang, F., Jonsson, A.H., Nathan, A., Wei, K., Millard, N., Xiao, Q., Gutierrez-Arcelus, M., Apruzzese, W., Watts, G.F.M., Weisenfeld, D., et al. (2022). Cellular deconstruction of inflamed synovium defines diverse inflammatory phenotypes in rheumatoid arthritis. Preprint at bioRxiv. https://doi.org/10.1101/2022.02.25.481990.
5. Tsokos, G.C. (2020). Autoimmunity and organ damage in systemic lupus erythematosus. Nat. Immunol. *21*, 605–614. https://doi.org/10.1038/s41590-020-0677-6.
6. Han, X., Wang, R., Zhou, Y., Fei, L., Sun, H., Lai, S., Saadatpour, A., Zhou, Z., Chen, H., Ye, F., et al. (2018). Mapping the mouse cell atlas by microwell-seq. Cell *172*, 1091–1107.e17. https://doi.org/10.1016/j.cell.2018.02.001.
7. Han, X., Zhou, Z., Fei, L., Sun, H., Wang, R., Chen, Y., Chen, H., Wang, J., Tang, H., Ge, W., et al. (2020). Construction of a human cell landscape at single-cell level. Nature *581*, 303–309.
8. Catrina, A.I., Svensson, C.I., Malmström, V., Schett, G., and Klareskog, L. (2017). Mechanisms leading from systemic autoimmunity to joint-specific disease in rheumatoid arthritis. Nat. Rev. Rheumatol. *13*, 79–86. https://doi.org/10.1038/nrrheum.2016.200.
9. Kazer, S.W., Aicher, T.P., Muema, D.M., Carroll, S.L., Ordovas-Montanes, J., Miao, V.N., Tu, A.A., Ziegler, C.G.K., Nyquist, S.K., Wong, E.B., et al. (2020). Integrated single-cell analysis of multicellular immune dynamics during hyperacute HIV-1 infection. Nat. Med. *26*, 511–518. https://doi.org/10.1038/s41591-020-0799-2.
10. Gawel, D.R., Serra-Musach, J., Lilja, S., Aagesen, J., Arenas, A., Asking, B., Bengnér, M., Björkander, J., Biggs, S., Ernerudh, J., et al. (2019). A validated single-cell-based strategy to identify diagnostic and therapeutic targets in complex diseases. Genome Med. *11*, 47. https://doi.org/10.1186/s13073-019-0657-3.
11. Brand, D.D., Latham, K.A., and Rosloniec, E.F. (2007). Collagen-induced arthritis. Nat. Protoc. *2*, 1269–1275. https://doi.org/10.1038/nprot.2007.173.
12. Browaeys, R., Saelens, W., and Saeys, Y. (2020). NicheNet: modeling intercellular communication by linking ligands to target genes. Nat. Methods *17*, 159–162. https://doi.org/10.1038/s41592-019-0667-5.
13. Ruscitti, P., Berardicurti, O., Cipriani, P., and Giacomelli, R.; TRACK study group (2021). Benefits of anakinra versus TNF inhibitors in rheumatoid arthritis and type 2 diabetes: long-term findings from participants furtherly followed-up in the TRACK study, a multicentre, open-label, randomised, controlled trial. Clin. Exp. Rheumatol. *39*, 403–406.
14. Okada, Y., Terao, C., Ikari, K., Kochi, Y., Ohmura, K., Suzuki, A., Kawaguchi, T., Stahl, E.A., Kurreeman, F.A.S., Nishida, N., et al. (2012). Meta-analysis identifies nine new loci associated with rheumatoid arthritis in the Japanese population. Nat. Genet. *44*, 511–516.
15. Brink, M., Lundquist, A., Alexeyenko, A., Lejon, K., and Rantapää-Dahlqvist, S. (2019). Protein profiling and network enrichment analysis in individuals before and after the onset of rheumatoid arthritis. Arthritis Res. Ther. *21*, 288.
16. Umekita, K., Miyauchi, S., Nomura, H., Umeki, K., and Okayama, A. (2019). Neutrophil-derived lactoferrin induces the inflammatory responses of rheumatoid arthritis synovial fibroblasts via Toll-like receptor 4. Clin. Exp. Rheumatol. *37*, 834–841.
17. Schurgers, E., Mertens, F., Vanoirbeek, J.A.J., Put, S., Mitera, T., De Langhe, E., Billiau, A., Hoet, P.H.M., Nemery, B., Verbeken, E., and Matthys, P. (2012). Pulmonary inflammation in mice with collagen-induced arthritis is conditioned by complete Freund's adjuvant and regulated by endogenous IFN-gamma. Eur. J. Immunol. *42*, 3223–3234. https://doi.org/10.1002/eji.201242573.
18. Stahl, J.L., Cook, E.B., Graziano, F.M., and Barney, N.P. (2003). Differential and cooperative effects of TNFalpha, IL-1beta, and IFNgamma on human conjunctival epithelial cell receptor expression and chemokine release. Invest. Ophthalmol. Vis. Sci. *44*, 2010–2015. https://doi.org/10.1167/iovs.02-0721.
19. Al-Roub, A., Al Madhoun, A., Akhter, N., Thomas, R., Miranda, L., Jacob, T., Al-Ozairi, E., Al-Mulla, F., Sindhu, S., and Ahmad, R. (2021). IL-1β and TNFα cooperativity in regulating IL-6 expression in adipocytes depends on CREB binding and H3K14 acetylation. Cells *10*, 3228.
20. Panzer, S., Madden, M., and Matsuki, K. (1993). Interaction of IL-1β, IL-6 and tumour necrosis factor-alpha (TNF-α) in human T cells activated by murine antigens. Clin. Exp. Immunol. *93*, 471–478.
21. Deon, D., Ahmed, S., Tai, K., Scaletta, N., Herrero, C., Lee, I.-H., Krause, A., and Ivashkiv, L.B. (2001). Cross-talk between IL-1 and IL-6 signaling pathways in rheumatoid arthritis synovial fibroblasts. J. Immunol. *167*, 5395–5403.
22. Schurgers, E., Mertens, F., Vanoirbeek, J.A.J., Put, S., Mitera, T., De Langhe, E., Billiau, A., Hoet, P.H.M., Nemery, B., Verbeken, E., and Matthys, P. (2012). Pulmonary inflammation in mice with collagen-induced arthritis is conditioned by complete F reund's adjuvant and regulated by endogenous IFN-γ. Eur. J. Immunol. *42*, 3223–3234.
23. Vogt, L.M., Kwasniewicz, E., Talens, S., Scavenius, C., Bielecka, E., Ekdahl, K.N., Enghild, J.J., Mörgelin, M., Saxne, T., Potempa, J., and Blom, A.M. (2020). Apolipoprotein E triggers complement activation in joint synovial fluid of rheumatoid arthritis patients by binding C1q. J. Immunol. *204*, 2779–2790.
24. Del Rey, M.J., Valín, Á., Usategui, A., Ergueta, S., Martín, E., Municio, C., Cañete, J.D., Blanco, F.J., Criado, G., and Pablos, J.L. (2019). Senescent synovial fibroblasts accumulate prematurely in rheumatoid arthritis tissues and display an enhanced inflammatory phenotype. Immun. Ageing *16*. 29–9.
25. Lau, L., Porciuncula, A., Yu, A., Iwakura, Y., and David, G. (2019). Uncoupling the senescence-associated secretory phenotype from cell cycle exit via interleukin-1 inactivation unveils its protumorigenic role. Mol. Cell Biol. *39*, e00586-18–e00518.
26. Straus, D.S., and Glass, C.K. (2007). Anti-inflammatory actions of PPAR ligands: new insights on cellular and molecular mechanisms. Trends Immunol. *28*, 551–558. https://doi.org/10.1016/j.it.2007.09.003.

27. Thomas, M., Bayha, C., Klein, K., Müller, S., Weiss, T.S., Schwab, M., and Zanger, U.M. (2015). The truncated splice variant of peroxisome proliferator-activated receptor alpha, PPARα-tr, autonomously regulates proliferative and pro-inflammatory genes. BMC Cancer *15*, 488.

28. Varga, T., Czimmerer, Z., and Nagy, L. (2011). PPARs are a unique set of fatty acid regulated transcription factors controlling both lipid metabolism and inflammation. Biochim. Biophys. Acta *1812*, 1007–1022.

29. Huang, L., Fang, X., Shi, D., Yao, S., Wu, W., Fang, Q., and Yao, H. (2020). MSP-RON pathway: potential regulator of inflammation and innate immunity. Front. Immunol. *11*, 569082.

30. Dinarello, C.A. (2000). Proinflammatory cytokines. Chest *118*, 503–508. https://doi.org/10.1378/chest.118.2.503.

31. Jacenik, D., Zielińska, M., Mokrowiecka, A., Michlewska, S., Małecka-Panas, E., Kordek, R., Fichna, J., and Krajewska, W.M. (2019). G protein-coupled estrogen receptor mediates anti-inflammatory action in Crohn's disease. Sci. Rep. *9*, 6749. https://doi.org/10.1038/s41598-019-43233-3.

32. Gosselin, D., and Rivest, S. (2011). Estrogen receptor transrepresses brain inflammation. Cell *145*, 495–497. https://doi.org/10.1016/j.cell.2011.04.018.

33. Kostoula, C., Shaker, T., Cerovic, M., Craparotta, I., Marchini, S., Butti, E., Pascente, R., Iori, V., Garlanda, C., Aronica, E., et al. (2019). TLR3 preconditioning induces anti-inflammatory and anti-ictogenic effects in mice mediated by the IRF3/IFN-beta axis. Brain Behav. Immun. *81*, 598–607. https://doi.org/10.1016/j.bbi.2019.07.021.

34. Stowell, N.C., Seideman, J., Raymond, H.A., Smalley, K.A., Lamb, R.J., Egenolf, D.D., Bugelski, P.J., Murray, L.A., Marsters, P.A., Bunting, R.A., et al. (2009). Long-term activation of TLR3 by poly(I:C) induces inflammation and impairs lung function in mice. Respir. Res. *10*, 43. https://doi.org/10.1186/1465-9921-10-43.

35. Cole, J.E., Navin, T.J., Cross, A.J., Goddard, M.E., Alexopoulou, L., Mitra, A.T., Davies, A.H., Flavell, R.A., Feldmann, M., and Monaco, C. (2011). Unexpected protective role for Toll-like receptor 3 in the arterial wall. Proc. Natl. Acad. Sci. USA *108*, 2372–2377. https://doi.org/10.1073/pnas.1018515108.

36. Ighe, A., Dahlström, Ö., Skogh, T., and Sjöwall, C. (2015). Application of the 2012 Systemic Lupus International Collaborating Clinics classification criteria to patients in a regional Swedish systemic lupus erythematosus register. Arthritis Res. Ther. *17*, 3. https://doi.org/10.1186/s13075-015-0521-9.

37. Tan, E.M., Cohen, A.S., Fries, J.F., Masi, A.T., McShane, D.J., Rothfield, N.F., Schaller, J.G., Talal, N., and Winchester, R.J. (1982). The 1982 revised criteria for the classification of systemic lupus erythematosus. Arthritis Rheum. *25*, 1271–1277. https://doi.org/10.1002/art.1780251101.

38. Yao, L., Pan, J., Setiadi, H., Patel, K.D., and McEver, R.P. (1996). Interleukin 4 or oncostatin M induces a prolonged increase in P-selectin mRNA and protein in human endothelial cells. J. Exp. Med. *184*, 81–92. https://doi.org/10.1084/jem.184.1.81.

39. Brown, T.J., Rowe, J.M., Liu, J.W., and Shoyab, M. (1991). Regulation of IL-6 expression by oncostatin M. J. Immunol. *147*, 2175–2180.

40. Zhang, F., Wei, K., Slowikowski, K., Fonseka, C.Y., Rao, D.A., Kelly, S., Goodman, S.M., Tabechian, D., Hughes, L.B., Salomon-Escoto, K., et al. (2019). Defining inflammatory cell states in rheumatoid arthritis joint synovial tissues by integrating single-cell transcriptomics and mass cytometry. Nat. Immunol. *20*, 928–942. https://doi.org/10.1038/s41590-019-0378-1.

41. Der, E., Ranabothu, S., Suryawanshi, H., Akat, K.M., Clancy, R., Morozov, P., Kustagi, M., Czuppa, M., Izmirly, P., Belmont, H.M., et al. (2017). Single cell RNA sequencing to dissect the molecular heterogeneity in lupus nephritis. JCI Insight *2*, e93009. https://doi.org/10.1172/jci.insight.93009.

42. Martin, J.C., Chang, C., Boschetti, G., Ungaro, R., Giri, M., Grout, J.A., Gettler, K., Chuang, L.S., Nayar, S., Greenstein, A.J., et al. (2019). Single-cell analysis of Crohn's disease lesions identifies a pathogenic cellular module associated with resistance to anti-TNF therapy. Cell *178*, 1493–1508.e20. https://doi.org/10.1016/j.cell.2019.08.008.

43. Kitsak, M., Sharma, A., Menche, J., Guney, E., Ghiassian, S.D., Loscalzo, J., and Barabási, A.L. (2016). Tissue specificity of human disease module. Sci. Rep. *6*, 35241. https://doi.org/10.1038/srep35241.

44. Frodlund, M., Dahlström, O., Kastbom, A., Skogh, T., and Sjöwall, C. (2013). Associations between antinuclear antibody staining patterns and clinical features of systemic lupus erythematosus: analysis of a regional Swedish register. BMJ Open *3*, e003608. https://doi.org/10.1136/bmjopen-2013-003608.

45. Sandborn, W.J., Feagan, B.G., Marano, C., Zhang, H., Strauss, R., Johanns, J., Adedokun, O.J., Guzzo, C., Colombel, J.F., Reinisch, W., et al. (2014). Subcutaneous golimumab induces clinical response and remission in patients with moderate-to-severe ulcerative colitis. Gastroenterology *146*, 85–95. https://doi.org/10.1053/j.gastro.2013.05.048.

46. Leal, R.F., Planell, N., Kajekar, R., Lozano, J.J., Ordás, I., Dotti, I., Esteller, M., Masamunt, M.C., Parmar, H., Ricart, E., et al. (2015). Identification of inflammatory mediators in patients with Crohn's disease unresponsive to anti-TNFalpha therapy. Gut *64*, 233–242. https://doi.org/10.1136/gutjnl-2013-306518.

47. Guttman-Yassky, E., Suárez-Fariñas, M., Chiricozzi, A., Nograles, K.E., Shemer, A., Fuentes-Duculan, J., Cardinale, I., Lin, P., Bergman, R., Bowcock, A.M., and Krueger, J.G. (2009). Broad defects in epidermal cornification in atopic dermatitis identified through genomic analysis. J. Allergy Clin. Immunol. *124*, 1235–1244.e58. https://doi.org/10.1016/j.jaci.2009.09.031.

48. Suárez-Fariñas, M., Tintle, S.J., Shemer, A., Chiricozzi, A., Nograles, K., Cardinale, I., Duan, S., Bowcock, A.M., Krueger, J.G., and Guttman-Yassky, E. (2011). Nonlesional atopic dermatitis skin is characterized by broad terminal differentiation defects and variable immune abnormalities. J. Allergy Clin. Immunol. *127*, 954–964.e1-4. https://doi.org/10.1016/j.jaci.2010.12.1124.

49. Arijs, I., De Hertogh, G., Lemaire, K., Quintens, R., Van Lommel, L., Van Steen, K., Leemans, P., Cleynen, I., Van Assche, G., Vermeire, S., et al. (2009). Mucosal gene expression of antimicrobial peptides in inflammatory bowel disease before and after first infliximab treatment. PLoS One *4*, e7984. https://doi.org/10.1371/journal.pone.0007984.

50. Keir, M.E., Fuh, F., Ichikawa, R., Acres, M., Hackney, J.A., Hulme, G., Carey, C.D., Palmer, J., Jones, C.J., Long, A.K., et al. (2021). Regulation and role of alphaE integrin and gut homing integrins in migration and retention of intestinal lymphocytes during inflammatory bowel disease. J. Immunol. *207*, 2245–2254. https://doi.org/10.4049/jimmunol.2100220.

51. Vancamelbeke, M., Vanuytsel, T., Farré, R., Verstockt, S., Ferrante, M., Van Assche, G., Rutgeerts, P., Schuit, F., Vermeire, S., Arijs, I., and Cleynen, I. (2017). Genetic and transcriptomic bases of intestinal epithelial barrier dysfunction in inflammatory bowel disease. Inflamm. Bowel Dis. *23*, 1718–1729. https://doi.org/10.1097/Mib.0000000000001246.

52. Turnier, J.L., Pachman, L.M., Lowe, L., Tsoi, L.C., Elhaj, S., Menon, R., Amoruso, M.C., Morgan, G.A., Gudjonsson, J.E., Berthier, C.C., and Kahlenberg, J.M. (2021). Comparison of lesional juvenile myositis and lupus skin reveals overlapping yet unique disease pathophysiology. Arthritis Rheumatol. *73*, 1062–1072. https://doi.org/10.1002/art.41615.

53. Yao, Y., Richman, L., Morehouse, C., de los Reyes, M., Higgs, B.W., Boutrin, A., White, B., Coyle, A., Krueger, J., Kiener, P.A., and Jallal, B. (2008). Type I interferon: potential therapeutic target for psoriasis? PLoS One *3*, e2737. https://doi.org/10.1371/journal.pone.0002737.

54. Guo, P., Luo, Y., Mai, G., Zhang, M., Wang, G., Zhao, M., Gao, L., Li, F., and Zhou, F. (2014). Gene expression profile based classification models of psoriasis. Genomics *103*, 48–55.

55. Ungethuem, U., Haeupl, T., Witt, H., Koczan, D., Krenn, V., Huber, H., von Helversen, T.M., Drungowski, M., Seyfert, C., Zacher, J., et al. (2010). Molecular signatures and new candidates to target the pathogenesis of

rheumatoid arthritis. Physiol. Genom. *42A*, 267–282. https://doi.org/10.1152/physiolgenomics.00004.2010.

56. Woetzel, D., Huber, R., Kupfer, P., Pohlers, D., Pfaff, M., Driesch, D., Häupl, T., Koczan, D., Stiehl, P., Guthke, R., and Kinne, R.W. (2014). Identification of rheumatoid arthritis and osteoarthritis patients by transcriptome-based rule set generation. Arthritis Res. Ther. *16*, R84. https://doi.org/10.1186/ar4526.

57. Ko, W.C.C., Li, L., Young, T.R., McLean-Mandell, R.E., Deng, A.C., Vanguri, V.K., Dresser, K., and Harris, J.E. (2021). Gene expression profiling in the skin reveals strong similarities between subacute and chronic cutaneous lupus that are distinct from lupus nephritis. J. Invest. Dermatol. *141*, 2808–2819. https://doi.org/10.1016/j.jid.2021.04.030.

58. Liu, J., Berthier, C.C., and Kahlenberg, J.M. (2017). Enhanced inflammasome activity in systemic lupus erythematosus is mediated via type I interferon-induced up-regulation of interferon regulatory factor 1. Arthritis Rheumatol. *69*, 1840–1849. https://doi.org/10.1002/art.40166.

59. Billi, A.C., Gharaee-Kermani, M., Fullmer, J., Tsoi, L.C., Hill, B.D., Gruszka, D., Ludwig, J., Xing, X., Estadt, S., Wolf, S.J., et al. (2019). The female-biased factor VGLL3 drives cutaneous and systemic autoimmunity. JCI insight *4*, e127291.

60. Tsoi, L.C., Hile, G.A., Berthier, C.C., Sarkar, M.K., Reed, T.J., Liu, J., Uppala, R., Patrick, M., Raja, K., Xing, X., et al. (2019). Hypersensitive IFN responses in lupus keratinocytes reveal key mechanistic determinants in cutaneous. J. Immunol. *202*, 2121–2130. https://doi.org/10.4049/jimmunol.1800650.

61. Sarkar, M.K., Hile, G.A., Tsoi, L.C., Xing, X., Liu, J., Liang, Y., Berthier, C.C., Swindell, W.R., Patrick, M.T., Shao, S., et al. (2018). Photosensitivity and type I IFN responses in cutaneous lupus are driven by epidermal-derived interferon kappa. Ann. Rheum. Dis. *77*, 1653–1664. https://doi.org/10.1136/annrheumdis-2018-213197.

62. Abernathy-Close, L., Lazar, S., Stannard, J., Tsoi, L.C., Eddy, S., Rizvi, S.M., Yee, C.M., Myers, E.M., Namas, R., Lowe, L., et al. (2021). B cell signatures distinguish cutaneous lupus erythematosus subtypes and the presence of systemic disease activity. Front. Immunol. *12*, 775353.

63. de Paiva, C.S., S.C.P., Trujillo-Vargas, C.M., Schaeffer, L., Yu, Z., and Britton, R.A. (2021). Evaluation of Immune Pathways in the Conjunctiva of Sjogren Syndrome Keratoconjunctivitis Sicca.

64. Tsuboi, H., Nakai, Y., Iizuka, M., Asashima, H., Hagiya, C., Tsuzuki, S., Hirota, T., Miki, H., Hagiwara, S., Kondo, Y., et al. (2014). DNA microarray analysis of labial salivary glands in IgG4-related disease: comparison with Sjogren's syndrome. Arthritis Rheumatol. *66*, 2892–2899. https://doi.org/10.1002/art.38748.

65. Takahashi, H., Tsuboi, H., Asashima, H., Hirota, T., Kondo, Y., Moriyama, M., Matsumoto, I., Nakamura, S., and Sumida, T. (2017). cDNA microarray analysis identifies NR4A2 as a novel molecule involved in the pathogenesis of Sjögren's syndrome. Clin. Exp. Immunol. *190*, 96–109. https://doi.org/10.1111/cei.13000.

66. Christmann, R.B., Wooten, A., Sampaio-Barros, P., Borges, C.L., Carvalho, C.R.R., Kairalla, R.A., Feghali-Bostwick, C., Ziemek, J., Mei, Y., Goummih, S., et al. (2016). miR-155 in the progression of lung fibrosis in systemic sclerosis. Arthritis Res. Ther. *18*, 155. https://doi.org/10.1186/s13075-016-1054-6.

67. Mantero JC, L.R. (2018). Expression Data from Skin Biopsies in Patients with Systemic Sclerosis. https://www.ncbi.nlm.nih.gov/geo/query/acc.cgi?acc=GSE95065.

68. Kodama, K., Y.L., Fuhlbrigge, R., and Fathman, C.G. (2015). Genome-Wide Gene Expression Profiles in the Pancreatic Lymph Nodes of At-Risk Autoantibody Positive Individuals.

69. Noble, C.L., Abbas, A.R., Cornelius, J., Lees, C.W., Ho, G.T., Toy, K., Modrusan, Z., Pal, N., Zhong, F., Chalasani, S., et al. (2008). Regional variation in gene expression in the healthy colon is dysregulated in ulcerative colitis. Gut *57*, 1398–1405. https://doi.org/10.1136/gut.2008.148395.

70. Trapnell, C., Cacchiarelli, D., Grimsby, J., Pokharel, P., Li, S., Morse, M., Lennon, N.J., Livak, K.J., Mikkelsen, T.S., and Rinn, J.L. (2014). The dynamics and regulators of cell fate decisions are revealed by pseudotemporal ordering of single cells. Nat. Biotechnol. *32*, 381–386. https://doi.org/10.1038/nbt.2859.

71. Institute, B. (2019). Picard Toolkit (Broad Institute).

72. Dobin, A., Davis, C.A., Schlesinger, F., Drenkow, J., Zaleski, C., Jha, S., Batut, P., Chaisson, M., and Gingeras, T.R. (2013). STAR: ultrafast universal RNA-seq aligner. Bioinformatics *29*, 15–21. https://doi.org/10.1093/bioinformatics/bts635.

73. Aran, D., Looney, A.P., Liu, L., Wu, E., Fong, V., Hsu, A., Chak, S., Naikawadi, R.P., Wolters, P.J., Abate, A.R., et al. (2019). Reference-based analysis of lung single-cell sequencing reveals a transitional profibrotic macrophage. Nat. Immunol. *20*, 163–172. https://doi.org/10.1038/s41590-018-0276-y.

74. Eraslan, G., Simon, L.M., Mircea, M., Mueller, N.S., and Theis, F.J. (2019). Single-cell RNA-seq denoising using a deep count autoencoder. Nat. Commun. *10*, 390. https://doi.org/10.1038/s41467-018-07931-2.

75. Butler, A., Hoffman, P., Smibert, P., Papalexi, E., and Satija, R. (2018). Integrating single-cell transcriptomic data across different conditions, technologies, and species. Nat. Biotechnol. *36*, 411–420. https://doi.org/10.1038/nbt.4096.

76. Lopez, R., Regier, J., Cole, M.B., Jordan, M.I., and Yosef, N. (2018). Deep generative modeling for single-cell transcriptomics. Nat. Methods *15*, 1053–1058. https://doi.org/10.1038/s41592-018-0229-2.

77. Krämer, A., Green, J., Pollard, J., and Tugendreich, S. (2014). Causal analysis approaches in ingenuity pathway analysis. Bioinformatics *30*, 523–530. https://doi.org/10.1093/bioinformatics/btt703.

78. Clough, E., and Barrett, T. (2016). The gene expression Omnibus database. Methods Mol. Biol. *1418*, 93–110. https://doi.org/10.1007/978-1-4939-3578-9_5.

79. Stekhoven, D.J., and Bühlmann, P. (2012). MissForest-non-parametric missing value imputation for mixed-type data. Bioinformatics *28*, 112–118. https://doi.org/10.1093/bioinformatics/btr597.

80. Stekhoven, D.J. (2022). missForest: Nonparametric Missing Value Imputation Using Random Forest.

81. Venables, W.N., and Ripley, B.D. (2002). Modern Applied Statistics with S, 4th Edition.

82. Hartig, F. (2022). DHARMa: Residual Diagnostics for Hierarchical (Multi-Level/Mixed) Regression Models.

83. Ighe, A., Dahlström, Ö., Skogh, T., and Sjöwall, C. (2015). Application of the 2012 Systemic Lupus International Collaborating Clinics classification criteria to patients in a regional Swedish systemic lupus erythematosus register. Arthritis Res. Ther. *17*, 3. https://doi.org/10.1186/s13075-015-0521-9.

84. Uribe, A.G., Vilá, L.M., McGwin, G., Sanchez, M.L., Reveille, J.D., and Alarcón, G.S. (2004). The systemic lupus activity measure-revised, the Mexican systemic lupus erythematosus disease activity index (SLEDAI), and a modified SLEDAI-2K are adequate instruments to measure disease activity in systemic lupus erythematosus. J. Rheumatol. *31*, 1934–1940.

85. Gladman, D., Ginzler, E., Goldsmith, C., Fortin, P., Liang, M., Urowitz, M., Bacon, P., Bombardieri, S., Hanly, J., Hay, E., et al. (1996). The development and initial validation of the systemic lupus international collaborating clinics American College of Rheumatology Damage Index for Systemic Lupus Erythematosus. Arthritis Rheum. *39*, 363–369. https://doi.org/10.1002/art.1780390303.

86. Xu, D., Lin, Y., Shen, J., Zhang, J., Wang, J., Zhang, Y., Zhang, H., Ning, L., Liu, P., Li, S., et al. (2020). Overproduced bone marrow neutrophils in collagen-induced arthritis are primed for NETosis: an ignored pathological cell involving inflammatory arthritis. Cell Prolif *53*, e12824. https://doi.org/10.1111/cpr.12824.

87. Sato, T., Satooka, H., Ichioka, S., Maruo, Y., and Hirata, T. (2020). Citrullinated fibrinogen is a target of auto-antibodies in interstitial lung disease in mice with collagen-induced arthritis. Int. Immunol. *32*, 533–545. https://doi.org/10.1093/intimm/dxaa021.

88. Guillaumet-Adkins, A., Rodríguez-Esteban, G., Mereu, E., Mendez-Lago, M., Jaitin, D.A., Villanueva, A., Vidal, A., Martinez-Marti, A., Felip, E., Vivancos, A., et al. (2017). Single-cell transcriptome conservation in cryopreserved cells and tissues. Genome Biol. *18*, 45. https://doi.org/10.1186/s13059-017-1171-9.

89. Zhang, H., Nestor, C.E., Zhao, S., Lentini, A., Bohle, B., Benson, M., and Wang, H. (2013). Profiling of human CD4(+) T-cell subsets identifies the T(H)2-specific noncoding RNA GATA3-AS1. J. Allergy Clin. Immunol. *132*, 1005–1008. https://doi.org/10.1016/j.jaci.2013.05.033.

90. Gierahn, T.M., Wadsworth, M.H., Hughes, T.K., Bryson, B.D., Butler, A., Satija, R., Fortune, S., Love, J.C., and Shalek, A.K. (2017). Seq-Well: portable, low-cost RNA sequencing of single cells at high throughput. Nat. Methods *14*, 395–398. https://doi.org/10.1038/nmeth.4179.

91. McCullagh, P., and J.A.N.. (1983). Generalized Linear Models, 2nd ed. (Routledge). https://doi.org/10.1201/9780203753736.

92. Lawless, J.F. (1987). Negative binomial and mixed Poisson regression. Can. J. Stat. *15*, 209–225. https://doi.org/10.2307/3314912.

93. Yau, K.K.W., Wang, K., and Lee, A.H. (2003). Zero-inflated negative binomial mixed regression modeling of over-dispersed count data with extra zeros. Biom. J. *45*, 437–452. https://doi.org/10.1002/bimj.200390024.

94. Bozdogan, H. (1987). Model selection and Akaike's Information Criterion (AIC): the general theory and its analytical extensions. Psychometrika *52*, 345–370.

95. Hartig, F. (2022). DHARMa: Residual Diagnostics for Hierarchical (Multi-Level/Mixed) Regression Models. R package version 0.4.5. http://florianhartig.github.io/DHARMa/.

96. Ripley, W.N.V.B.D. (2022). Modern Applied Statistics with S, 4th. https://doi.org/10.1007/978-0-387-21706-2.

97. Qiu, X., Hill, A., Packer, J., Lin, D., Ma, Y.A., and Trapnell, C. (2017). Single-cell mRNA quantification and differential analysis with Census. Nat. Methods *14*, 309–315. https://doi.org/10.1038/nmeth.4150.

98. Li, H., Courtois, E.T., Sengupta, D., Tan, Y., Chen, K.H., Goh, J.J.L., Kong, S.L., Chua, C., Hon, L.K., Tan, W.S., et al. (2017). Reference component analysis of single-cell transcriptomes elucidates cellular heterogeneity in human colorectal tumors. Nat. Genet. *49*, 708–718. https://doi.org/10.1038/ng.3818.

99. Ayer, M., Brunk, H.D., Ewing, G.M., Reid, W.T., and Silverman, E. (1955). An empirical distribution function for sampling with incomplete information. Ann. Math. Stat. *26*, 641–647.

100. Traag, V.A., Waltman, L., and van Eck, N.J. (2019). From Louvain to Leiden: guaranteeing well-connected communities. Sci. Rep. *9*, 5233. https://doi.org/10.1038/s41598-019-41695-z.

101. QIAGEN. IPA KnowledgeBase. https://qiagen.secure.force.com/KnowledgeBase/KnowledgeIPAPage?id=kA41i000000L5m1CAC.

102. Kodama, K., L.Y., Fuhlbrigge, R., and Fathman, C.G. (2015), GEO Accession viewer. https://www.ncbi.nlm.nih.gov/geo/query/acc.cgi?acc=GSE66413.

103. Mantero, J.C., and R.L.. (2018). GEO Accession Viewer. available at. https://www.ncbi.nlm.nih.gov/geo/query/acc.cgi?acc=GSE95065.

104. Qiao, M., and Q.S.. (2021). GEO Accession Viewer. available at. https://www.ncbi.nlm.nih.gov/geo/query/acc.cgi?acc=GSE181318.

105. Berthier, C.C., Bethunaickan, R., Gonzalez-Rivera, T., Nair, V., Ramanujam, M., Zhang, W., Bottinger, E.P., Segerer, S., Lindenmeyer, M., Cohen, C.D., et al. (2012). Cross-species transcriptional network analysis defines shared inflammatory responses in murine and human lupus nephritis. J. Immunol. *189*, 988–1001. https://doi.org/10.4049/jimmunol.1103031.

STAR★METHODS

KEY RESOURCES TABLE

REAGENT or RESOURCE	SOURCE	IDENTIFIER
**Biological samples**		
Organ samples for scRNA-seq and Histological analysis	This paper	Supplementary file
sera from SLE patients for measurement of the UR proteins	[44]	Supplementary file
**Critical commercial assays**		
Luminex Assay for protein	Bio-techne (R&D)	Custom code: LXSAHM-17<br>Lot number: L143454
EILSA for TGFB1	Bio-techne (R&D)	Catalog: DB100C
**Deposited data**		
scRNA-seq data of CIA mouse	This paper	GEO: GSE206659
bulk-RNA data of treated UC patients	[45]	GEO: GSE92415
bulk-RNA data of treated CD patients	[46]	GEO: GSE52746
bulk-RNA of AD patients	[47]	GEO: GSE16161
bulk-RNA of AD patients	[48]	GEO: GSE32924
bulk-RNA of CD patients	[49]	GEO: GSE16879
bulk-RNA of CD patients	[50]	GEO: GSE179285
bulk-RNA of CD patients	[51]	GEO: GSE75214
bulk-RNA of JM patients	[52]	GEO: GSE148810
bulk-RNA of LN patients	[52]	GEO: GSE32591
bulk-RNA of PSO patients	[53]	GEO: GSE14905
bulk-RNA of PSO patients	[54]	GEO: GSE181318
bulk-RNA of RA patients	[55]	GEO: GSE1919
bulk-RNA of RA patients	[56]	GEO: GSE55235
bulk-RNA of lupus patients	[57]	GEO: GSE112943
bulk-RNA of SLE patients	[52]	GEO: GSE148810
bulk-RNA of lupus patients	[58–62]	GEO: GSE81071
bulk-RNA of SS patients	[63]	GEO: GSE176510
bulk-RNA of SS patients	[64,65]	GEO: GSE40568
bulk-RNA of SSc patients	[66]	GEO: GSE81292
bulk-RNA of SSc patients	[67]	GEO: GSE95065
bulk-RNA of T1D patients	[68]	GEO: GSE66413
bulk-RNA of UC patients	[69]	GEO: GSE11223
bulk-RNA of UC patients	[50]	GEO: GSE179285
bulk-RNA of UC patients	[51]	GEO: GSE75214
**Experimental models: Organisms/strains**		
model organism:<br>mouse model of arthritis<br>Mouse: DBA1/J	Jordbruksverket (Stockholm, Sweden) and GemPharmatech Co., Ltd. (Nanjing, China)	N/A
**Software and algorithms**		
Full analysis pipeline	This paper	https://github.com/SDTC-CPMed/multi-organ_DigiTwin.
MONOCLE	[70]	http://monocle-bio.sourceforge.net/.
James Nemesh, McCarrol's lab Drop-seq Core Computational Protocol v1.0.1	http://mccarrolllab.com	http://mccarrolllab.co
Drop-Seq tools v1.12	http://mccarrolllab.com	http://mccarrolllab.com

(Continued on next page)

Continued		
REAGENT or RESOURCE	SOURCE	IDENTIFIER
bcl2fastq v2.19.1	https://emea.support.illumina.com/sequencing/sequencing_software/bcl2fastq-conversion-software.html	https://emea.support.illumina.com/sequencing/sequencing_software/bcl2fastq-conversion-software.html
Picard software v2.9.0	[71]	https://github.com/broadinstitute/picard.
STAR software v2.5.3	[72]	http://code.google.com/p/rna-star/.
2019SingleR v1.0.6	[73]	https://github.com/dviraran/SingleR.
Deep count autoencoder (DCA) v0.2.3	[74]	https://github.com/theislab/dca.
Seurat v3.1.2	[75]	https://cran.r-project.org/web/packages/Seurat/index.html.
scVI v0.7.1	[76]	https://github.com/romain-lopez/scVI-reproducibility.
NicheNet v1.0.0	[12]	https://github.com/saeyslab/nichenetr
Ingenuity Pathway Analysis vQ1 2021 and vQ4 2020	[77]	https://digitalinsights.qiagen.com/products-overview/discovery-insights-portfolio/analysis-and-visualization/qiagen-ipa/
rstatix v0.7.0	https://CRAN.R-project.org/package=rstatix	https://CRAN.R-project.org/package=rstatix.
GEO2R	[78]	https://www.ncbi.nlm.nih.gov/geo/geo2r/
missForest v1.5	[79,80]	https://CRAN.R-project.org/package=missForest
MASS v7.3-54	[81]	https://cran.r-project.org/web/packages/MASS/index.html.
DHARMa v0.4.5	[82]	https://cran.r-project.org/web/packages/DHARMa/index.html.
stats v4.0.4	https://rdrr.io/r/stats/stats-package.html	https://rdrr.io/r/stats/stats-package.html
Other		
The indexed reference GRCm38 (June 2017, Ensembl)	https://www.ensembl.org/index.html	https://www.ensembl.org/index.html
Ensembl genes GRCh38.p13, Downloaded June 6, 2020	http://www.ensembl.org/biomart/martview	http://www.ensembl.org/biomart/martview
GWAS-associated genes downloaded from DisGeNET (data downloaded on February 9, 2021)	https://www.disgenet.org/	https://www.disgenet.org/

RESOURCE AVAILABILITY

Lead contact

Further information and requests for resources and reagents should be directed to and will be fulfilled by the lead contact, Mikael Benson. (mikael.benson@ki.se).

Materials availability

This study did not generate new unique reagents.

Data and code availability

- Single-cell RNA-seq data have been deposited at GEO and are publicly available as of the date of publication. Accession numbers are listed in the key resources table.
- All original code has been deposited at GitHub and is publicly available as of the date of publication. DOIs are listed in the key resources table.
- Any additional information required to reanalyze the data reported in this paper is available from the lead contact upon request.

EXPERIMENTAL MODEL AND SUBJECT DETAILS

Mouse model of arthritis

Male DBA1/J mice aged between 8-12 weeks were housed in the Linköping Animal Housing Unit of the Faculty of Health Sciences and kept under standard temperature and light conditions. Experiments were conducted according to the Swedish Animal Welfare Act and ethical permission was granted by the Ethical Committee Board, Norra Stockholms Djurförsöksetiska nämnd (permission number: 6798/18). For the independent histology analysis, male DBA1/J mice were purchased from GemPharmatech (China) and were maintained in a specific pathogen-free animal facility. All animal studies were performed in accordance with protocols approved by the Animal Experimental Ethics Committee of Xuzhou Medical University (permission number: 202012A162).

Human data

Samples were obtained from 304 patients (263 women, 41 men) classified with SLE according to the 1982 American College of Rheumatology and/or the Fries's diagnostic principle[83] (Data S1). All subjects had provided oral and written informed consent. The study protocol was approved by the Regional Ethics Review Board in Linköping (M75-08/2008). All subjects were included in the prospective and observational research program *Clinical Lupus Register in North-Eastern Gothia* at the Rheumatology Unit, Linköping University Hospital48. Patients were not involved in the design, conduct, reporting or dissemination plans of our research. Serum was available from each patient at two different time-points from which disease activity had been assessed by the clinical SLE disease activity index (SLEDAI) and damage accrual by the Systemic Lupus International Collaborating Clinics/ACR damage index (SDI).[84,85] The recent treatment of the patients prescribed at the previous visit was included in the clinical information (Data S1).

METHOD DETAILS

Study design

Our aims were to characterize, organize and prioritize disease-associated organome-, cellulome- and genome-wide changes in IMIDs (Figure 1). We combined single cell and bulk multi-organ profiling of mouse and human IMIDs. We found complex and heterogeneous organome-wide changes in a mouse model of CIA, which could be organized into a multi-organ multicellular disease model (MO-MCDM). In this MO-MCDM all organs interacted, without evident hierarchy. Despite the organome-wide molecular changes only joints showed signs of inflammation. This contrast led to the identification of an overriding structure in which shared transcriptional programs were switched on or off by variable combinations of URs. Analyses of IMID patients who did or did not respond to treatment with anti-TNF, as well as more than 600 blood samples from SLE patients, supported a graded on/off switch regulated by variable combinations of URs, which have the potential for personalized diagnostics and therapeutics.

CIA mouse model generation

For scRNA-seq analysis, CIA was established following a previously described method.[11] Six mice were immunized with 100 μg (50 μL) bovine collagen II (BC-II, Chondrex, USA) emulsified with 50 μL Complete Freund's Adjuvant (CFA) (Sigma-Aldrich, USA) in 1:1 ratio, via intradermal injection near the base of the tail. A booster immunization was administered on day 20 with 100 μg of BC-II emulsion (prepared with 1:1 incomplete Freund's adjuvant (IFA)) and injected at the base of the tail. 100 μL of Phosphate-Buffered Saline (PBS) was injected similarly to control mice. The severity of arthritic limbs was scored on a 0–4 scale, 0: normal; 1: swelling and redness in one digit; 2: swelling and redness in more than one digit or swelling and redness in one digit, wrist and ankle; 3: Swelling and redness presenting in paw and digits; 4: maximum inflammation of limb involving all joints and digits as described in the protocol by Brand et al.[11] The arthritic score for each mouse was the sum of the scores of arthritic limbs. The mice were sacrificed when they achieved scores of 8–12 or after they had been immunized for 60 days under isoflurane anesthesia via cervical dislocation. The joint, blood, draining lymph nodes, lung, thymus, skin, limb muscle, spleen, liver, and kidney were collected for further analysis.

For the independent histology analysis, four 8-week male DBA1/J mice were immunized intradermally in the proximal tail with 100 μL of emulsified chicken type II collagen (2 mg/mL, Chondrex, USA)/CFA (1 mg/mL). The clinical arthritis score was evaluated for each limb from 0 to 4 with a maximal score of 16 for each mouse.[86] The healthy control and severe CIA mice (clinical score >8) were sacrificed, and the knee joints, lungs, livers, kidneys, skin, and hindlimb muscles were collected after heart perfusion.[17,87]

Histological analysis

Whole knee joints, lungs, livers, kidneys, skin, and hindlimb muscles from healthy control and CIA mice were fixed in 4% formaldehyde. Joints were further decalcified with Decalcification Solution (ServiceBio, G1107, China) for 7 days. The specimens were then embedded in paraffin and sagittal sections (4 μm) were cut. The sections were stained with hematoxylin and eosin (H&E, Sigma-Aldrich, USA) for the histology analysis. Histological sections were assessed for infiltration of cells into the synovial cavity resulting in inflammation, proliferation of cells in the synovial layer, and bone erosion.

Sample cryopreservation

All dissected organ samples were placed into suitable tubes with freezing solution (10% DMSO and 90% FBS), placed into a CoolCell LX box (Corning, USA), and frozen with gradually decreasing temperature (1°C/min) to −80°C. The samples were then stored at −175°C until further analysis.

Sample thawing

Before digesting all the organs and harvesting the single cells, the cryopreserved samples were thawed following[88] with slight differences. Briefly, cryopreserved samples were quickly thawed in a 37°C water bath with continuous agitation, then transferred into 15 mL centrifuge tubes with 1 mL pre-warmed thaw solution (90% Hibernate-A and 10% FBS) and incubated at room temperature for 1 minute. Next, 2 mL, 5 mL, and 5 mL thaw solutions were added into the centrifuge tube, separated by 1-minute incubation. The samples were then centrifuged at 350 × g for 5 minutes. Lastly, the samples were resuspended with 1 mL Hibernate-A, after which the supernatant was removed and incubated until the next step.

Single cell suspension

The thymus, spleen, and lymph node were thawed as described above, and were passed through a 70 μm strainer to collect cells in a 50 mL centrifuge tube. After being centrifuged at 350 × g for 5 minutes, cells were resuspended with 5 mL red blood cell lysis buffer for 5 minutes. The lysis reaction was quenched by adding medium (90% RPMI-1640 with 10% FBS). Cells were centrifuged at 350 × g for 5 minutes and washed thrice to remove the lysis buffer. Single cell suspensions were prepared with RPMI-1640 at a density of 1 × 10[illegible] cells/mL. Samples from different organs (whole knee joint, muscle, lung, skin, liver, and kidney) were quickly transferred into 75 mm dishes with 1 mL DMEM after thawing, and then minced into ~1mm pieces with scissors. Next, pieces of organ samples were transferred into 15 mL centrifuge tubes containing 5 mL DMEM. Different organ samples were treated with different enzymes for different durations (Data S1). After dissociation, another 5 mL DMEM with 10% FBS was added to the 15 mL centrifuge tubes. Dissociated cells were centrifuged at 350 × g for 5 minutes after passing through a 70 μm strainer, after which the cells were washed thrice with PBS. Single cells were resuspended into RPMI-1640 at a density of 1×10^5 cells/mL for cell loading. Peripheral blood mononuclear cells (PBMCs) were isolated as previously described.[89] Briefly, 0.5 mL peripheral blood was diluted with an equal volume of PBS (calcium free), which was further loaded on the top of 1 mL Lymphoprep followed by centrifugation at 800 × g for 30 minutes at room temperature in a swinging bucket rotor with the brake off. PBMCs were retrieved and washed with PBS. Erythroid cells of different organs and peripheral blood of each mouse were removed using RBC Lysis Buffer (Bio Legend, USA) (Data S1). Single cell suspensions were prepared by resuspension of PBMCs with RPMI-1640 at a density of 1×10^5 cells/mL.

scRNA-seq wet-lab protocol

All scRNA-seq experiments were performed using the Seq-Well technique.[90] Briefly, prepared single cell suspensions were co-loaded with barcoded and functionalized oligo-dT beads (Chemgenes, USA; cat. no. MACOSKO-2011-10) on microwell arrays synthesized as described.[90] For each sample, 20,000 live cells were loaded onto an array to bind with oligo-dT beads. The arrays, covered with plasma-treated polycarbonate membranes, were placed in a 37°C incubator for 30 minutes. Next, beads were collected to perform cell lysis, hybridization, reverse transcription, and whole transcriptome amplification. Libraries were then prepared for each sample using the Nextera XT DNA Library Preparation Kit (Illumina, USA; cat. no. FC-131-1096) according to the manufacturer's instructions. Libraries from three samples were pooled together and sequenced using the NextSeq 500/550 system, and sequencing results were analyzed as described below.

Cytokine analyses in peripheral blood

Approximately 100 μL of blood were collected from ten healthy control DBA1/J mice, as well as CIA mice at week three (before symptom onset, n = 12), week eight (early stage after symptom onset, n = 12) and week 15 (later stage after symptom onset, n = 9) after CIA induction by retro-orbital bleeding. Twenty-five μL of serum were used for assaying inflammatory cytokines using LEGENDplex™ Mouse Inflammation Panel (13-plex) (CAT: 740446, BioLegend, USA), including IL-1α, IL-1β, IL-6, IL-10, IL-12p70, IL-17A, IL-23, IL-27, MCP-1, IFN-β, IFN-γ, TNF, and GM-CSF. The assay was performed according to the manufacturer's protocol and the data were collected on a BD FACSAria III flow cytometer and analyzed by Flowjo. The mean fluorescence intensity of each cytokine of the standards was used for calculating the standard curve for each cytokine using a log-log curve fit. The difference in concentration of each cytokine between the different time points was calculated using the Wilcoxon rank sum test, as described above.

The analysis of the UR protein expressions in SLE sera

We used the clinical variables, patient information, drug treatment and protein levels (Data S1) to estimate the association between the protein levels and the patient phenotype, organ, damage, and disease activity. To preprocess the data, we log-transformed the protein levels, and used random forest imputation[79] to impute the missing values. We removed the observations where the response variable was not available, and we scaled the input variables to zero mean and unit variance. In case of treatment information, we only included drugs that were used to treat at least ten patients. As the distribution of the response variables differed, we fitted different regression models for each respective response variable. We used a logistic regression,[91] negative binomial regression[92] and zero inflated negative binomial regression[93] to predict the patient phenotype, organ damage, and disease activity respectively. We also

CellPress
OPEN ACCESS

used Akaike Information Criteria (AIC)[94] to perform variable selection. These models returned the coefficient estimates as well as a p value, which tested a hypothesis that the estimate is zero. A positive coefficient estimate reflects that the patients with high response variables had high protein levels, and we further referred to it as a positive association. In contrast, a negative coefficient estimate reflects that the patients with high response variables had low protein levels, and we further referred to it as a negative association. To assess whether the tested models follow their assumptions, we standardized the residuals between 0 and 1, then compared them against the assumed distribution and performed outlier and dispersion checks. We also plotted the standardized residuals against the rank transformed predicted variable where we expected a uniform distribution.[95] R functions missForest,[79] glm,[79] glm.nb,[96] were used to perform the analysis, and function simulateResiduals[95] was used to investigate the residuals.

Measurement of the UR proteins in SLE sera

We measured the protein levels of the 18 predicted UR proteins of most disease relevant IMID_SP in the serum of 304 patients at two separate phlebotomies using human magnetic multiplex beads assay (R&D, Bio-Techne, USA) according to the manufacturer's instructions. The five-parameter logistic curve was used to generate the standard curve. The Limit of Detection (LOD) was defined as the standard point with the lowest concentration of an analyte that can reliably distinguish signal from background noise. The Lower Limit of Quantification (LLOQ) is defined as the standard with the lowest concentration. The Upper Limit of Quantification (ULOQ) was defined as the standard point with the highest concentration. If protein values lay outside of the interval between LLOD and ULOQ but higher than LOD, we used extrapolated data for further analysis. We excluded IL-17 and GM-CSF from the analysis as 86% and 75% of measurements respectively were below LOD.

QUANTIFICATION AND STATISTICAL ANALYSIS

Organ prioritization

The relevance of the organs for CIA development was tested in a pilot study, in which at least one sample from each organ was sequenced (Data S1) and processed as described.[10] In short, the data from each sample were extracted, and poor-quality cells were sorted out as described below. Sequencing data from two of the organs, liver and kidney, did not meet quality criteria ($\leq$25 cells with 10,000 reads per cell) and were excluded from further analysis. The data from the remaining samples was knn-smoothed ($k = 12$), whereafter DEGs were identified between sick and healthy individuals for each organ separately using Monocle[70,97] as described in.[10] For comparative analysis between organs, 40 cells were bootstrapped from each group of healthy and sick individuals, for ten sampling rounds, for the differential expression analysis. The number of DEGs (Benjamini-Hochberg adjusted p value (FDR) < 0.05) for each sampling was compared and the five organs with the highest number of DEGs (joint, lung, muscle, skin, and spleen) were selected for downstream analysis (Data S1 and Figure S1A).

scRNA-seq data processing

The single cell data from the different mice samples were processed into digital gene expression matrices following James Nemesh, McCarrol's lab Drop-seq Core Computational Protocol (version 1.0.1, Drop-Seq tools v1.12) (http://mccarrolllab.com) using bcl2fastq (v2.19.1) conversion and Picard software (v2.9.0). The indexed reference for alignment of the reads was generated from GRCm38 (June 2017, Ensembl) using STAR software (v2.5.3).[72] Only primary alignments towards the reference genome were considered during downstream analyses, according to the mapping quality using STAR software. The quality of cells was assessed by having a minimum of 10,000 reads, 400 transcripts, 200 genes and less than 20% of mitochondrial genes per cell. The five organs with the highest number of DEGs based on the organ prioritization described above, namely, joint, lung, muscle, skin and spleen, were then analyzed together. Outliers were removed based on an overestimation of transcripts count (i.e., cells with more than 6,000 transcripts) due to the risk of duplicates in the library. For a gene to be included in the data, it needed to be identified in at least 10% of the cells.

Clustering and cell type identification

We used a reference-based approach to identify cell types. As a reference dataset, we used mouse bulk expression data of sorted cell populations available in the R package SingleR (v1.0.6).[73] To preprocess the data for cell type identification, we only retained 6,395 shared genes for both the bulk expression data and the single cell data. The resulting single cell data was denoised by the deep count autoencoder (DCA, v0.2.3)[74] with the default settings. This method has an in-built data normalization and outputs 1) a denoised dataset corrected for dropouts and varying library sizes, where each data value represents the expected (denoised) gene expression, and 2) a latent representation of the denoised data in a 32-dimensional latent space. Existing reference-based single cell type identification such as[73] or[98] uses correlation measures, either Spearman or Pearson, to match the single cell observations to the reference data. While Pearson correlation measures linear relationships, Spearman correlation is more general in the sense that it accounts for monotonic (i.e., non-linear) relationships. Accordingly, for each reference point, a single cell observation having the highest Spearman's correlation to that reference was found, and then a Monotonic Regression (MR)[99] was computed with the reference vector as the input variable and the natural logarithm of the denoised single cell expression of the selected cell as the output variable in the regression. The exponents of the predicted values from the MR were then treated as reference data expressions in the scale of the single cell data. This means that the rounded exponents of the fitted MR values for all reference data points were used as inputs in the DCA

that was estimated in the data validation step, and the latent representations outputted by the autoencoder were used in the following step. The latent space observations obtained from both the single cell data and from the bulk data were clustered together by the Leiden's algorithm.[100] In the Leiden's algorithm, we set the size of local neighborhood to 30 and the resolution parameter (which determines the number of clusters) was set in such a manner that 70% of cells in each resulting cluster matched the same cell type. Briefly, we started with a resolution parameter of 0.5, resulting in a low number of clusters. For each single cell observation in each computed cluster, a match was computed by finding a reference point that belongs to that cluster and has the highest Spearman correlation to the denoised gene expression of the single cell. The purity of a cluster was determined by computing the proportion of single cell observations within that cluster that had the same reference type. The resolution was increased until each cluster had a satisfactory purity, which resulted in resolution = 1. Finally, all single cell data points within a cluster were labeled with the reference label corresponding to the dominant reference match within that cluster (Data S1). All clusters recognized as the same cell type were merged for further analysis, resulting in 13 groups of cells (Figure 2A). The cell types identified, as well as those unidentified due to a lack of cell types in the reference, were further validated, or identified, using marker genes as described below.

Data normalization and DEG analysis

To calculate DEGs in the dataset, we used the single cell variational inference (scVI, v0.7.1) framework.[76] First, the variational inference model was set-up based on the UMI-count data, reducing batch effect based on the input samples, i.e., individual mice and organs, after which the model was trained using default parameters. The DEGs were then identified between CIA and healthy mice for each cell type in each organ separately, using the 'change'-mode. The significant DEGs were identified as those with 'is_de_fdr_0.05 = True' in the scVI differential expression output. To infer the direction of change, we used the mean log(fold change) ('lfc_mean') values produced, where a positive fold change (FC) indicated upregulation while a negative FC indicated downregulation in cells from CIA compared to healthy mice (Data S2). The data used for differential expression analysis were normalized by the scVI autoencoder, correcting for variation in sequencing depth. The normalized expression matrix and the latent space representation data were used for single cell downstream analyses as further described. To compare how much the lists of DEGs differed between cell types and organs, the Jaccard index was calculated for each pair of gene lists.

Cell type identification using marker genes

Marker genes were used to validate the cell types identified by the Leiden algorithm, and to obtain the identity of clusters that were not represented by any reference data point. For all organs combined, the marker genes were calculated based on DEG analysis as described above. The marker genes were defined as those being significantly differentially expressed between each cell type or cluster of unknown identity (based on the previous identification) and all other cell types in the dataset (Data S1). We then searched for the known marker genes (Table S1) within the sets of cell type-specific marker genes. If marker genes of a certain cell type were enriched in a cluster, i.e., a positive FC, the cluster was identified accordingly.

For each cell type identified in any of the organs, the variation in cell type proportion over organs was calculated using ANOVA, adjusting the p values by Holm correction.

Cell-cell interaction analysis

The interactions between cells were identified by analyzing the data using NicheNet (v1.0.0).[12] NicheNet is an R package developed for identification of inter-cellular interactions based on lists of potential interacting genes and a database of known upstream regulators (URs) to target interactions. For these analyses, we used the lists of DEGs between CIA and healthy mice to find the interactions which change due to arthritis. As NicheNet requires human gene symbols as input, the mouse genes were translated into their human orthologs based on the Ensembl genes (http://www.ensembl.org/biomart/martview, GRCh38.p13, Downloaded June 6, 2020). These human orthologs were used for all downstream analyses based on these cell-cell interactions.

The cut-off to define the expressed genes in the data was set according to the author's recommendation, to give 5,000 to 10,000 expressed genes for the sender and receiver cell population independently. First, the previous transformation of the scVI-normalized expression data inverse logarithm was applied (10-1). Next, genes with a mean expression level $\geq 1 \times 10^{-5}$ in the population of cells were defined as expressed. The interactions were then identified, based on the lists of DEGs, between each pair of cell types using the default analysis set-up. For each interaction identified, all of the potential target genes in the source cell type were identified using the get_weighted_ligand_target_links() function and its default settings. To focus our analyses on the strongest interactions of the networks, we included only those with a Pearson Correlation Score (PCC) > 0, meaning that the target genes of the interactions are enriched among the differentially expressed genes.

When analyzing inter-organ interactions, the UR-target interactions identified by NicheNet were curated only to include those that are biologically feasible between organs. For this aim, we used Ingenuity Pathway Analysis (IPA, Q4 2020, Qiagen, Germany) to specify the cellular location known for each potential UR (Data S6). The list of inter-organ interactions was then curated to include only URs located in extracellular space, as they have the potential to be transported through the blood.

MCDM construction and UR prioritization

For each organ separately, a MCDM was constructed based on predicted molecular interactions between all cell types in the organ. The predictions were inferred using NicheNet[12] as described above. The MCDM thus consists of cell types as nodes and unique

CellPress
OPEN ACCESS

interactions as lines. A unique interaction represents one cell type-UR-cell type combination, thus enabling multiple edges, based on different UR, between each pair of nodes. The MO-MCDM was created in the same way, but only based on inter-organ interactions as described above. Thus, the nodes represent cell types in each organ, and the lines represented interactions between cell types in different organ.

The URs were then prioritized based on their downstream effect. First, they were ranked based on the total number of predicted downstream target genes that they were predicted to regulate, in all cell types and organs combined. Secondly, they were ranked based on the number of downstream cell types and organs which they were predicted to target.

Pathway enrichment analysis

Identification of pathways was performed with IPA. We used the core analysis in the IPA software to identify canonical pathways based on a list of DEGs (e.g., all the potential targets of all the URs in the different organs). When this analysis was completed for each cell type and organ separately, the Bayes factors from the differential expression analysis were included to define the direction of change due to arthritis.

IPA consists of a global network that is based on manual curation of a vast body of medical literature and biomedical databases, which is continuously updated.[101] The core analysis in IPA (parameters: species = mouse) was used to identify pathways that were significantly enriched among the list of genes. Statistical analysis was performed using Fisher's exact test, right tailed, within the IPA software (Q1 2021 and Q4 2020 version).[77] All pathways with $p < 0.05$ were considered significantly enriched. Pathway activation direction was indicated by IPA activation z-score as activated (z-score >0) or inhibited (*Z* score <0).

The association between UR-target expression and arthritis score

The association between UR-target expression and arthritis score was calculated by pairwise comparison of the target mean expression levels between cells from mouse joints with mild arthritis (score 1–3), or severe arthritis (score 4) (Data S1), as well as healthy control mice, using a Wilcoxon rank sum test. The gene names of the normalized data from scVI analysis were first translated to their human orthologs as previously described. Thereafter, the data were standardized, producing a mean expression of zero and standard deviation of one, over all genes within each cell. The mean expression level of all UR target genes was calculated for each cell. The differences in target expression were then computed, in a pair-wise manner between the groups using the wilcox_test() and add_significance() functions in the R package rstatix (v0.7.0), producing p values adjusted for multiple testing by the Holm correction.

Connective pathway analysis

Pathway analysis was first conducted using IPA, as described above, on all DEGs for each dataset/cell type. To systematically assess similarities and differences between pathways in the different groups, (i.e. joint and muscle for CIA or inflamed and non-inflamed organ sites for IMIDs), we first clustered the pathways based on gene list similarities. To do so, the Jaccard-index was calculated, and values of 1-Jaccard-index used as distances for hierarchical clustering. Clustering was performed using the *hclust()* function in R with Ward's method (i.e. parameter *ward.D2*). Only genes differentially expressed in at least one cell type (CIA)/dataset (IMIDs) within each group (active and inactive disease organ) were considered for clustering. The dendrogram from the hierarchical clustering was next transformed into a tree-like structure, in which each node represents one pathway. To prioritize clusters for downstream analyses further, each pathway was labelled as "activated," "inhibited," "no_direction," or "not_significant" in each group separately. Labelling was based on the ratio of datasets showing a specific direction of change (indicated by the IPA activation z-score). For example, in the CIA data, the "Acute Phase Response Signaling" pathway was found significantly enriched (overlap $p < 0.05$) in six cell types out of 12 in muscle: activated (z-score >0) in two cell types and inhibited (z-score <0) in six cell types. Since the pathway was inhibited in a higher number of datasets, "Acute Phase Response Signaling" was labelled as "inactive" in muscle. In cases in which there was an equal number of cell types with a pathway that was activated and inhibited or if the direction could not be predicted (z-score = NA), the pathway was labelled "no_direction", and if none of the cell types showed significant enrichment of the pathway, it was labelled "not_significant". This oversimplification helps to achieve an overview of general behavior of pathways in the distinct groups, and to prioritize clusters of pathways for further analyses.

For further validation, KEGG pathway analysis was also conducted on all DEGs for each cell type in muscle and joint using "enrichKEGG" function from R package "clusterProfiler". Then, connective pathway analysis was performed on the KEGG pathways with $p < 0.05$. First, similarities and differences were assessed between pathways using the Jaccard-index. Clustering was performed as previously described and divided into two programs. To assess whether connective pathway analysis using KEGG pathways has comparable results as using IPA, similarities between programs from connective pathway analysis of KEGG and IPA were compared using enrichment analysis (two-sided Fisher's exact test). Since all pathways in KEGG have different pathway names, pathways were not directly compared between KEGG and IPA; instead, the enrichment analyses were performed on gene content of each program using Fisher's exact test. All genes enriched in KEGG pathways were used as background for the analyses.

Prediction of URs regulating specific programs/subprograms in connective pathway analysis

To identify potential URs of each program/subprogram from the connective pathway analysis, we performed enrichment analyses. For each potential UR-program/subprogram pair we computed the enrichment of the URs downstream targets among the pathway-associated genes (right tail Fisher's exact test). All genes in connective pathway analysis were used as background. Enrichment

p values were next combined with Fisher's method over all cell types in the joint and muscle separately. For each respective organ, the URs with significant combined p values, corrected for multiple testing with the Benjamini-Hochberg procedure (FDR <0.05), were ranked by the lowest combined FDR values (Data S6).

Similarly, URs were identified in the meta-analyses of IMIDs, where enrichment p values were combined over all datasets representative of inflamed organs and non-inflamed organs separately. To identify specific URs for (UC and CD, enrichment p values were combined over all datasets representative of the disease and condition (e.g., all datasets representative of inflamed organs in CD).

Differential expression analysis of expression profiling data from immune-mediated inflammatory diseases

We systematically mined the Gene Expression Omnibus (GEO) database for expression profiling datasets from different IMIDs. Each dataset included samples from at least three patients and three healthy controls (Data S1). The search included 32 different datasets from ten different IMIDs, namely RA, UC, CD, PSO, SLE, systemic sclerosis (SSc), Sjögren's syndrome (SS), atopic dermatitis (AD), juvenile myositis (JM), and type 1 diabetes (T1D).[47–53,55–57,61,63,65,66,69,102–105] Using GEO2R81, we identified DEGs between patients and healthy control samples (detailed in Data S1). The data were annotated by the National Center for Biotechnology Information (NCBI) and adjusted for multiple testing using the Benjamini-Hochberg procedure. The dataset was next filtered to include only significant DEGs (FDR <0.05), with the associated gene symbol for further core analysis using the IPA software. If more than 5,000 DEGs were found between patients and healthy controls, the top 5,000 DEGs (lowest FDR value) were used for the core analysis using the IPA software (Q1 2021 and Q4 2020 version), owing to the computational limitation of upload allowance. The core analysis in IPA (parameters: species = human) was used to identify canonical pathways as described above. Enrichment analyses were performed by applying Fisher's exact test (right tailed)). In case two or more datasets were representative of the same disease and condition (for example UC inflamed organs), enrichment p values were combined with Fisher's method in all downstream analyses. To check for similarities between subprograms from connective pathway analysis of CIA and IMID data, we computed the enrichment of pathways between each subprogram of CIA and each subprogram of IMID. As a background for the analyses, all pathways included in the connective pathway analysis were used.

Genome-wide association study (GWAS) enrichment analyses within programs and subprograms

Genome-wide association study (GWAS) gene enrichment analysis (Fisher exact test, right tailed) of programs and subprograms in connective pathway analysis in both CIA and IMIDs was performed for each cell type/dataset separately. All DEGs within that cell type/dataset were used as a background. The GWAS-associated genes were downloaded from DisGeNET (data downloaded on February 9, 2021). For the CIA data, GWAS genes associated with 'Rheumatoid Arthritis' were included, identifying 777 genes. For the IMIDs data, we included GWAS genes associated with, RA: 'Rheumatoid Arthritis' (777 genes), CD: 'Crohn disease' (515 genes), lupus (except lupus nephritis (LN)): 'Lupus Erythematosus', 'Lupus Erythematosus, Cutaneous', 'Lupus Erythematosus, Discoid', 'Lupus Erythematosus, Subacute Cutaneous', 'Lupus Erythematosus, Systemic', and 'Neuropsychiatric Systemic Lupus Erythematosus' (626 genes), LN: 'Lupus Nephritis' (53 genes), SS: "Primary Sjögren's syndrome" and "Sjogren's Syndrome" (49 genes), T1D: 'Diabetes Mellitus, Insulin-Dependent', 'Diabetes Mellitus, Ketosis-Prone', 'Diabetes, Autoimmune', and 'Neonatal insulin-dependent diabetes mellitus' (485 genes), AD: 'Dermatitis, Atopic' and 'Dermatitis, Atopic, 2' (143 genes), JM: 'Adult type dermatomyositis', 'Dermatomyositis', 'Dermatomyositis, Childhood Type', and 'Myositis' (46 genes), PSO: 'Psoriasis' (416 genes), SSc: 'Systemic Scleroderma' (171 genes), and UC: 'Ulcerative Colitis' (452 genes).

Prioritization of URs in IMIDs

The upstream analysis of the core analysis in IPA (parameters: species = human, Q1 2021 and Q4 2020 version) was used to predict URs for all IMIDs separately based on DEGs. If more than 5,000 DEGs were found between patients and healthy controls, the top 5,000 DEGs (lowest FDR value) were used for the upstream analysis. We focused on URs that belonged to one of the following categories: "G-protein coupled receptor", or "cytokine", or "growth factor", or "ligand-dependent nuclear receptor", or "transmembrane receptor'' and whose downstream targets were significantly enriched among DEGs (FDR <0.05; Fisher exact test right-tailed; IPA). To predict further the URs regulating the specific programs/subprograms in IMIDs, we performed enrichment analysis as described above.

Overlap between programs from all analyzed IMIDs and individual IMIDs

To test if the programs (IMID_P1 and IMID_P2) derived from all analyzed IMIDs overlapped with programs from individual IMIDs in inflamed and non-inflamed organ sites, separately, we performed Fisher's exact tests (right tailed), using all pathways in connective pathway analysis as a background, followed by correction for multiple testing using the Benjamini-Hochberg procedure. These analyses were repeated for IMID_ subprograms (IMID_SPs) and subprograms from individual IMIDs. Disease pathways were defined as all pathways significantly enriched in a particular disease and inflammation state (IPA, $p < 0.05$). Enriched pathways were considered those whose combined $p < 0.05$.

Treatment effect on IMIDs

We mined the Gene Expression Omnibus (GEO) database for the analysis of anti-TNF treatment effects on any of the previously analyzed IMIDs. Each dataset included samples from at least three patients (before and after treatment) and three healthy controls.

The search included two different datasets, one from treated UC (GSE92415)[45] and one from treated CD (GSE52746).[46] We identified DEGs between treated responders and untreated patients, as well as between untreated patients and healthy control samples. Pathway enrichment analyses were performed as described above. We then performed enrichment analysis of the significantly enriched pathways among the pathways of the program/subprograms from connective pathway analysis, as described above.

Subsequently, DEGs were identified for responders and non-responders separately, between untreated patients vs. control and treated patients vs. control, as well as between treated non-responders vs. treated responders, as described above. Enrichment analyses of the significantly enriched pathways of the program/subprograms were performed for the untreated patients vs. control, as described above. The URs for non-responders and responders before treatment were predicted using IPA, as described above. Focusing on URs of molecule types, ‘‘G-protein coupled receptor’’, ‘‘cytokine’’, ‘‘growth factor’’, ‘‘ligand-dependent nuclear receptor’’, and ‘‘transmembrane receptor’’, we next assessed how the downstream targets of each UR predicted for the non-responders overlapped with the downstream targets of TNF for each program/subprogram separately using enrichment analysis as described above. Among those URs whose downstream targets were predicted to be enriched in at least one program/subprogram, we prioritized them that showed the potential to take over the effect of TNF based on the predicted effect of any potential activators or inhibitors. The following criteria were used: 1) the activation z-score from IPA being similarly positive or negative in both responders and non-responders; 2) the FC direction being higher (if positive z-score) or lower (if negative z-score) in non-responders vs. control compared to responders vs. control; and 3) a significant positive (if positive z-score) or negative (if negative z-score) FC in non-responders vs. responders after treatment.

To test the potential of other URs to take over the effect of TNF, we analyzed data from GSE92415. The URs and enriched pathways were first inferred using IPA, based on the top 5,000 (lowest p value) of the 10,145 DEGs identified between anti-TNF responders before treatment (I.e., week 0, N = 32) and healthy control (N = 21), and the 11,351 DEGs between anti-TNF non-responders before treatment (N = 27) and healthy control (N = 21). We only focused on URs of molecule types, ‘‘G-protein coupled receptor’’, ‘‘cytokine’’, ‘‘growth factor’’, ‘‘ligand-dependent nuclear receptor’’, and ‘‘transmembrane receptor. First, we tested if the downstream genes of each alternative UR were enriched among the TNF downstream genes, for each main- and subprogram separately (Figure S6E). For each UR, whose downstream genes were predicted to be enriched in at least one main- or subprogram, we further checked if their z-scores and FCs in responders and non-responders followed the expectations for overtaking the effect of TNF among the non-responders, but not for the responders, after treatment. Such assumptions can be made, since TNF is known to be an activator, showing a positive z-score and FC among both responders and non-responders before treatment compared to controls, and since it is assumed that any UR taking over the effect of TNF will share many of its downstream genes. Specifically, we assumed that if the UR is an activator, its z-score should be positive in both responders and non-responders, while the direction of its FC should be higher in non-responders vs. control compared to responders vs. control (for example, if the FC is negative in responders vs. control, it should be zero or positive in non-responders vs. control) in order to take over the downstream effect of TNF. If the UR is an inhibitor, we instead expected a negative z-score among both responders and non-responders, and that the direction of the FC should be lower in non-responders vs. control compared to responders vs. control. We additionally assumed that an UR which has taken over the effect of TNF should be significantly upregulated (activator), or downregulated (inhibitor), in treated non-responders compared to treated responders, between which a total of 2,922 DEGs were identified.

Cell Reports Medicine, Volume *4*

Supplemental information

Multi-organ single-cell analysis reveals an on/off switch system with potential for personalized treatment of immunological diseases

Sandra Lilja, Xinxiu Li, Martin Smelik, Eun Jung Lee, Joseph Loscalzo, Pratheek Bellur Marthanda, Lang Hu, Mattias Magnusson, Oleg Sysoev, Huan Zhang, Yelin Zhao, Christopher Sjöwall, Danuta Gawel, Hui Wang, and Mikael Benson

Supplementary information

Supplementary Figures:

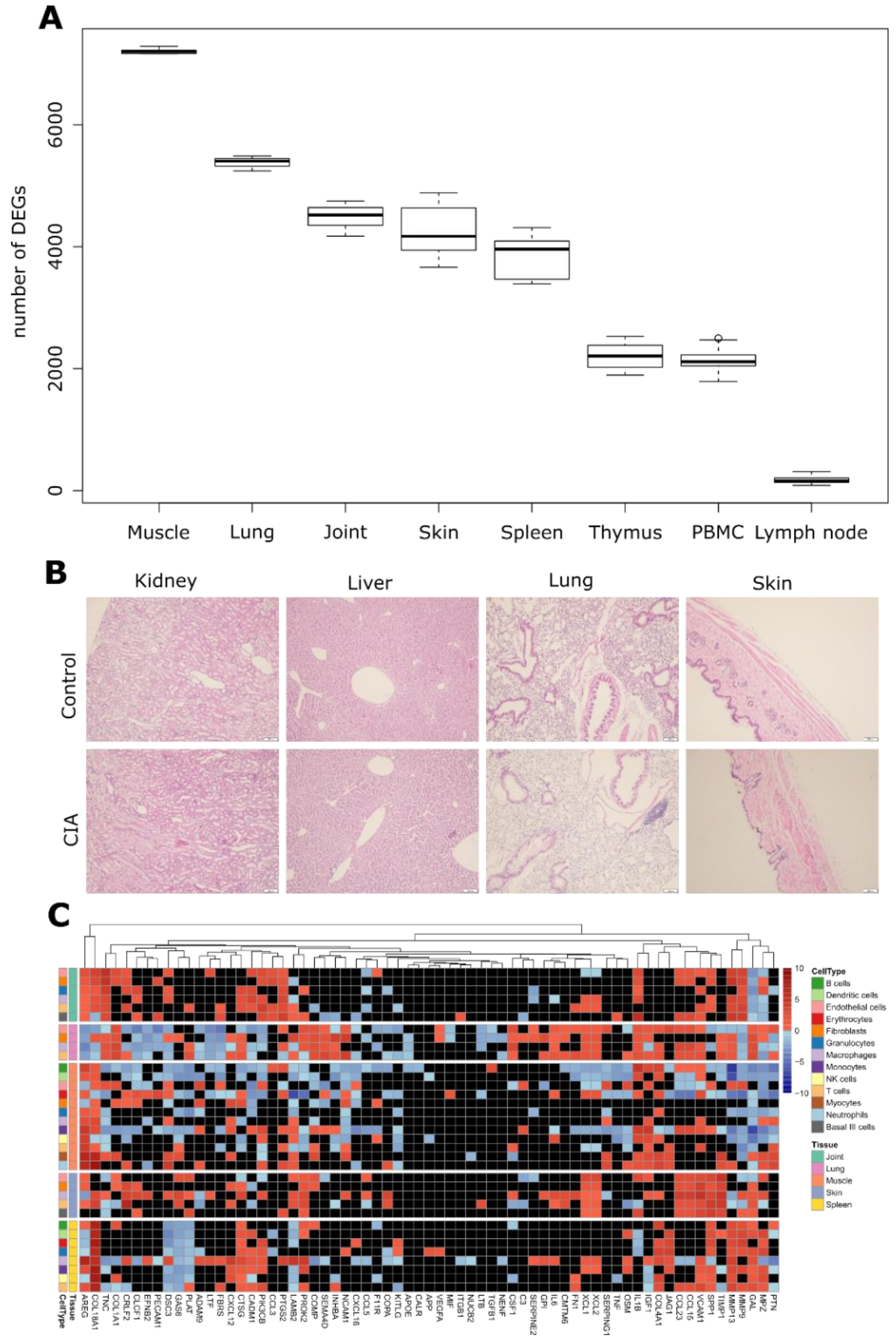

Figure S1. The number of differentially expressed genes, H&E analysis, the FCs of all URs from the different organs. Related to Figure 2.
(A) Boxplot showing the number of DEGs produced between a subset of 40 CIA and 40 healthy cells from each of the different organs. Cells were sampled 10 times, following differential expression analysis using Monocle, as described in[S1]. The boxes indicate the first and third quartile, with whiskers. The line represents the median value, and outliers are marked as circles. **(B)** Representative H&E images of kidney, liver, lung, and skin from healthy control mice and CIA mice, shown at a magnification of 100 × (scar bar 100 μm). **(C)** Heatmap showing the FCs of all URs identified by the NicheNet analyses, in all five organs.

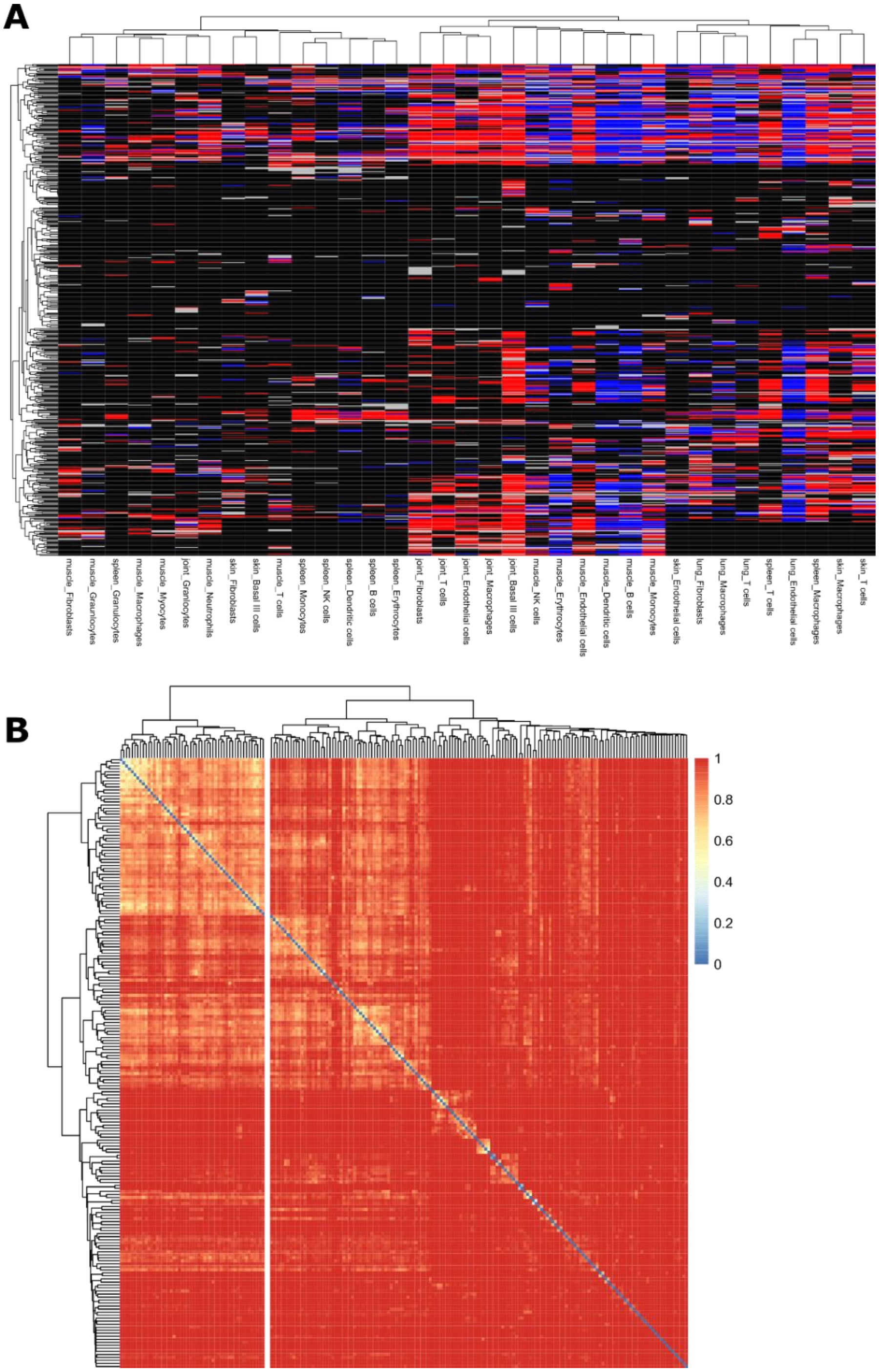

Figure S2. Pathways enrichment analysis and Connective Pathway Analysis. Related to STAR methods. (A) Pathways being significantly enriched in at least one cell type and organ. Heatmap showing the z-scores from pathway enrichment analysis. The color indicates the pathways inferred direction where red = activated, blue = inhibited, grey = unknown, black = not significant. (B) Connective Pathway Analysis on KEGG pathways. Two main programs were identified- program 1 (right) and program 2 (left). Color represents 1-Jaccard Index.

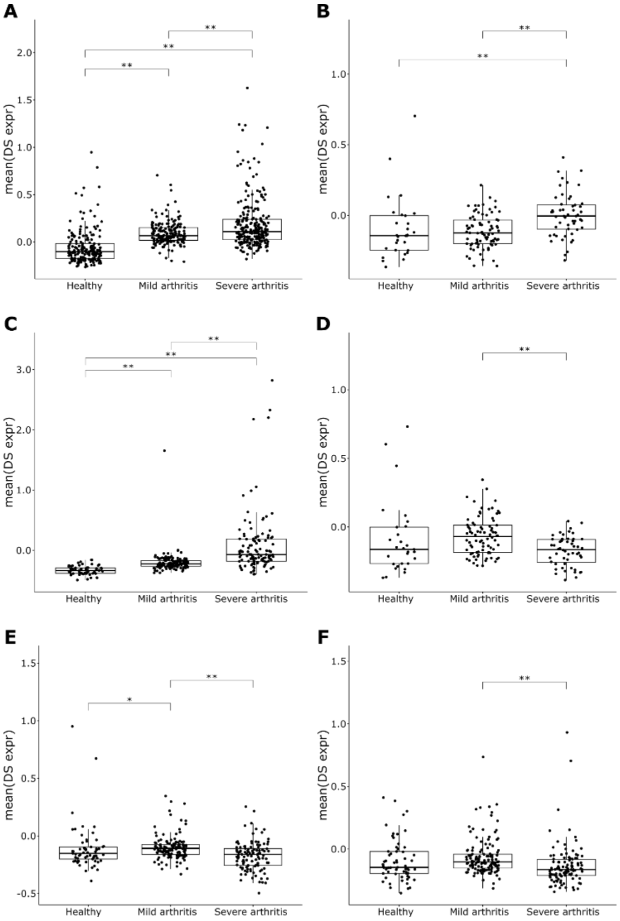

Figure S3. Mean standardized expression level per cell in healthy, mild arthritic, and severe arthritic joint of the downstream genes (DS) of upstream regulators. Related to STAR methods and Figure 2.
(A) TNF in Fibroblasts, (B) IL1B in T cells, (C) MPZ in Macrophages, (D) LTF in T cells, (E) LTF in Macrophages, and (F) DSC3 in Macrophages. Each dot represents an individual cell. The FDR-corrected *p* values were calculated using Wilcoxon rank sum test, * $p < 0.05$ and ** $p < 0.01$.

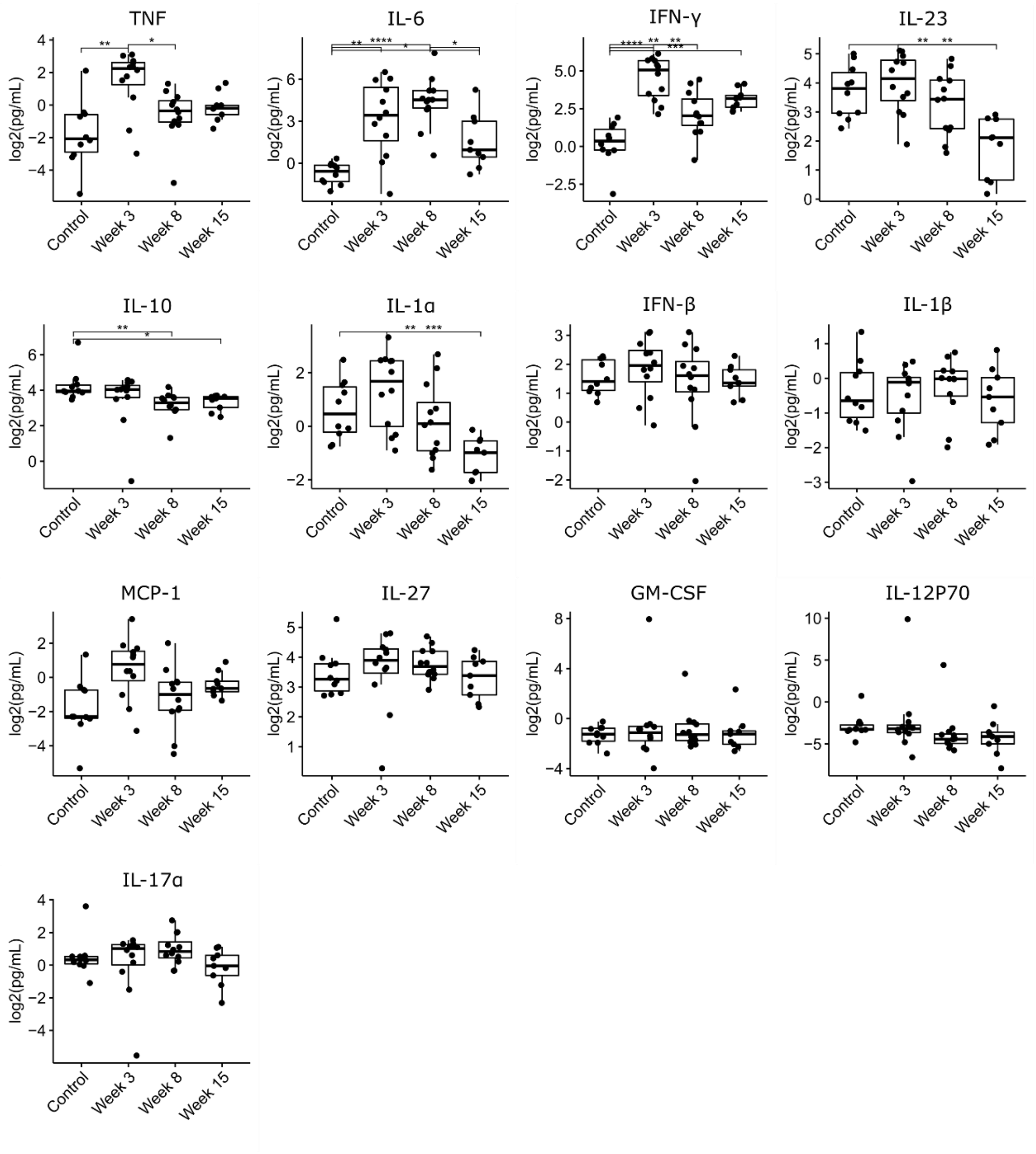

Figure S4. Time series analysis of cytokine protein levels in sera from CIA and control mice. Related to STAR methods.

The proteins were measured at different time points after collagen stimulation and in healthy control mice. *$p < 0.05$, **$p < 0.01$, ***$p < 0.001$, ****$p < 0.0001$.

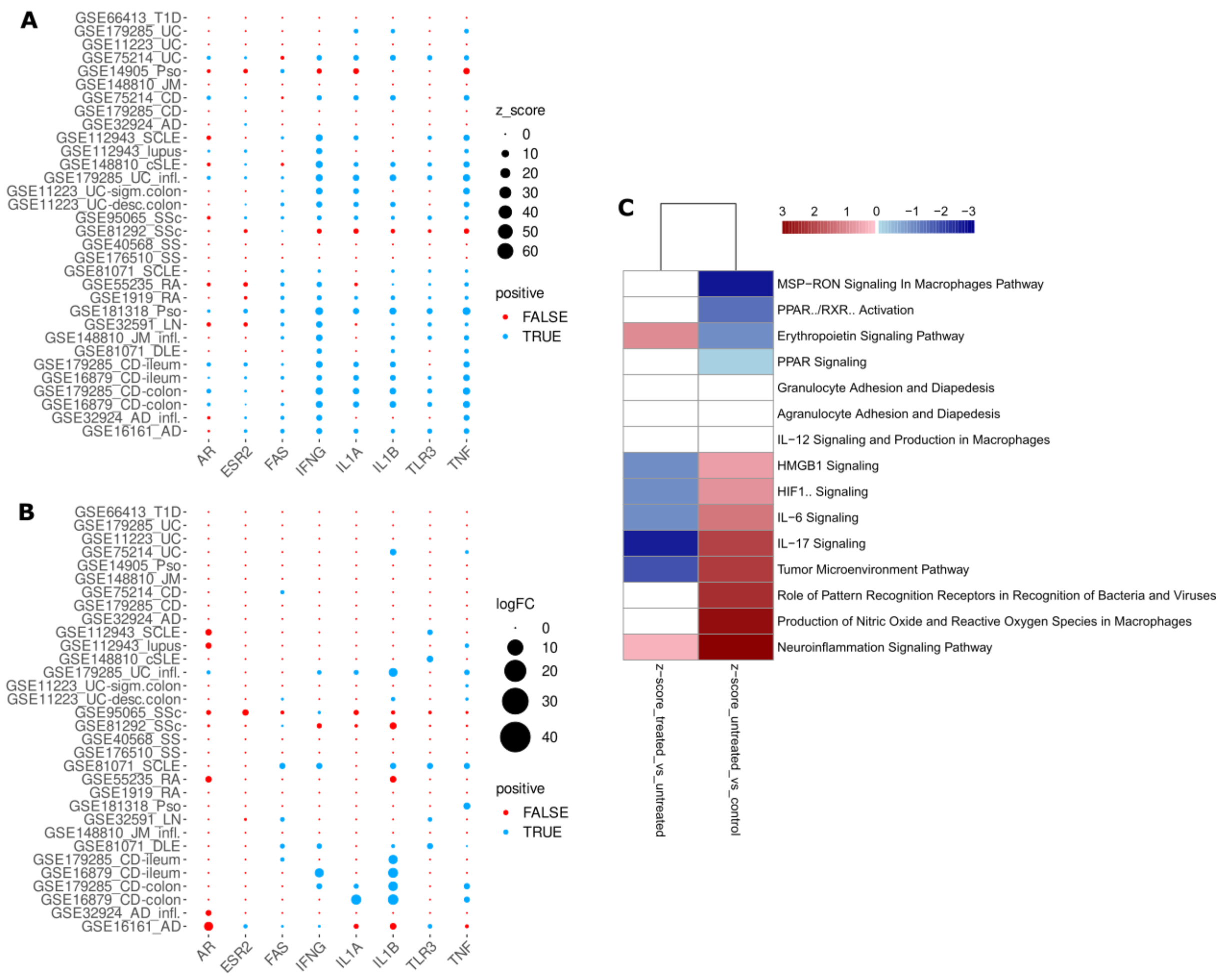

Figure S5. Predicted activities, fold changes of the shared URs and predicted direction of pathways of IMID sub-program 1.6. Related to the STAR methods and Figure 7.

Dot plot showing the (A) predicted activities and (B) fold changes, of the shared URs of IMID_SP1.6. The URs are presented in columns and the datasets are presented in rows. Blue represents increased activity or fold-change, and red represents the opposite. The size of a dot represents (A) z score or (B) log fold change(logFC). (C) Predicted direction of pathways in IMID sub-program 1.6 due to disease (right) and after treatment (left). A positive z-score (red) indicates that the pathway was upregulated due to disease or treatment, and a negative z-score (blue) indicates that the pathway was downregulated. The directions are inferred based on pathway analysis of GSE52746.

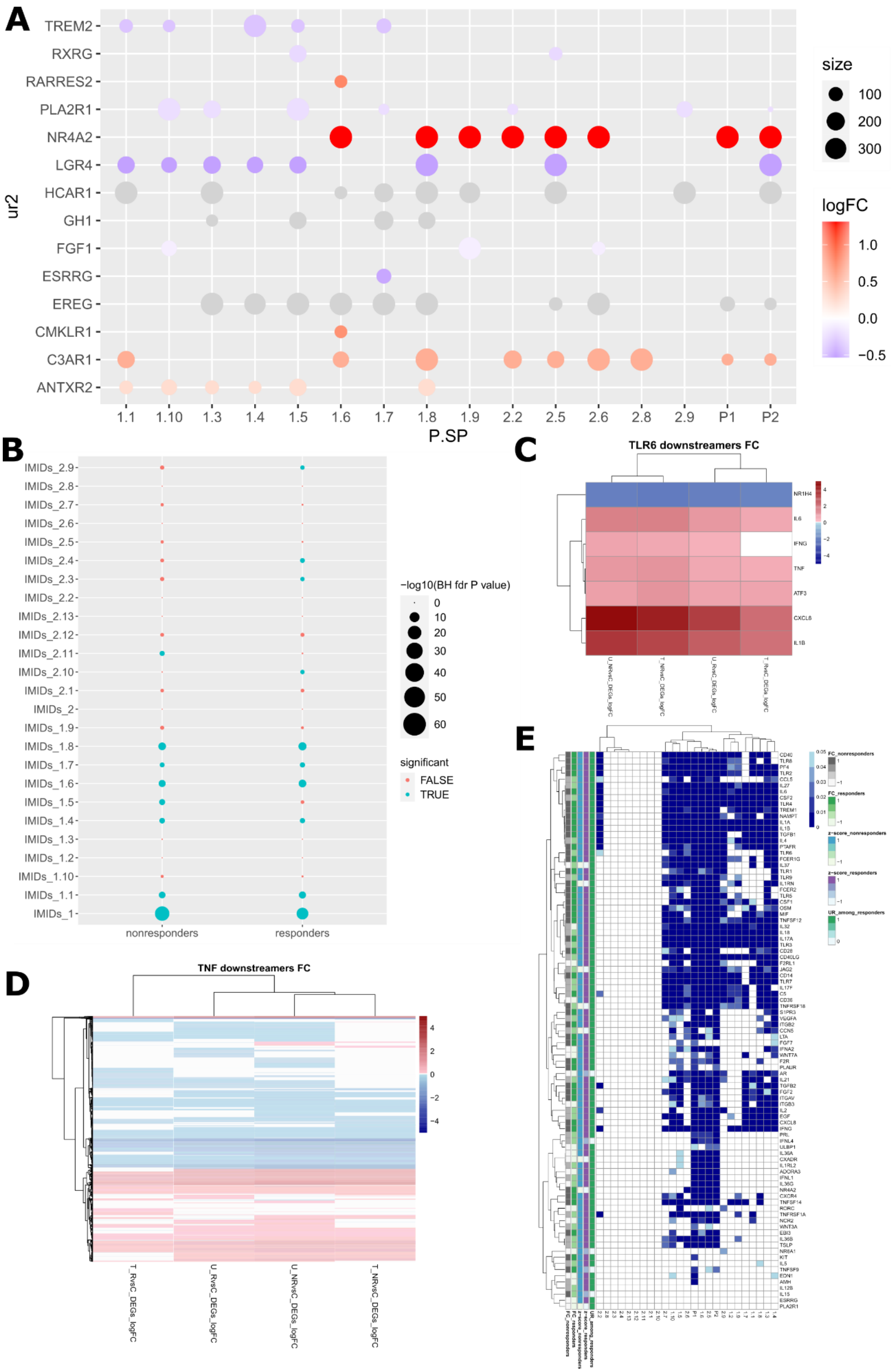
A
TREM2
RXRG
RARRES2
PLA2R1
NR4A2
LGR4
HCAR1
GH1
FGF1
ESRRG
EREG
CMKLR1
C3AR1
ANTXR2
ur2
1.1
1.10
1.3
1.4
1.5
1.6
1.7
1.8
1.9
2.2
2.5
2.6
2.8
2.9
P1
P2
P.SP
size
100
200
300
logFC
1.0
0.5
0.0
−0.5
B
IMIDs_2.9
IMIDs_2.8
IMIDs_2.7
IMIDs_2.6
IMIDs_2.5
IMIDs_2.4
IMIDs_2.3
IMIDs_2.2
IMIDs_2.13
IMIDs_2.12
IMIDs_2.11
IMIDs_2.10
IMIDs_2.1
IMIDs_2
IMIDs_1.9
IMIDs_1.8
IMIDs_1.7
IMIDs_1.6
IMIDs_1.5
IMIDs_1.4
IMIDs_1.3
IMIDs_1.2
IMIDs_1.10
IMIDs_1.1
IMIDs_1
nonresponders
responders
−log10(BH fdr P value)
0
10
20
30
40
50
60
significant
FALSE
TRUE
C
TLR6 downstreamers FC
NR1H4
IL6
IFNG
TNF
ATF3
CXCL8
IL1B
D
TNF downstreamers FC
4
2
0
−2
−4
T_RvsC_DEGs_logFC
U_RvsC_DEGs_logFC
U_NRvsC_DEGs_logFC
T_NRvsC_DEGs_logFC
E

Figure S6. Analysis of responder, non-responder, treated/untreated responder, treated/untreated non-responder and control. Related to STAR methods.
(A) Upstream regulators of the subprograms predicted to be regulated by *TNF* that were not changed after the anti-TNF medication (GSE92415). URs are presented in rows, sub-programs in columns. Dots represent significant overlap between UR DS and TNF DS (Fisher Exact test, left-tailed, corrected for multiple testing, Methods). Size represents Odds Ratio (OR), and color of the nodes shows the FC of UR in the UC responders to anti-TNF vs. healthy controls (red denotes upregulation in UC, purple downregulation, whereas grey denotes that the UR was not significantly differentially expressed or not measured on the microarray GSE92415). (B) Main- and subprogram association of pathways enriched among the DEGs for non-responders vs. control and responders vs. control, right-tailed Fisher's exact test. (C) and (D) Fold changes of the (C) TLR 6 and TNF (D) downstream genes between (from left to right): treated responders vs. control, untreated responders vs. control, untreated non-responders vs. control, and treated non-responders vs. control. The colored gradient indicates significant ($p < 0.05$) positive (red) and negative (blue) FCs. (E) Enrichment analysis between the downstream genes of each UR predicted among the non-responders and the downstream genes of TNF. The blue gradient indicates the FDR-corrected p value. White indicates nonsignificant $p > 0.05$. The enrichment analysis was performed by Fisher's exact test.

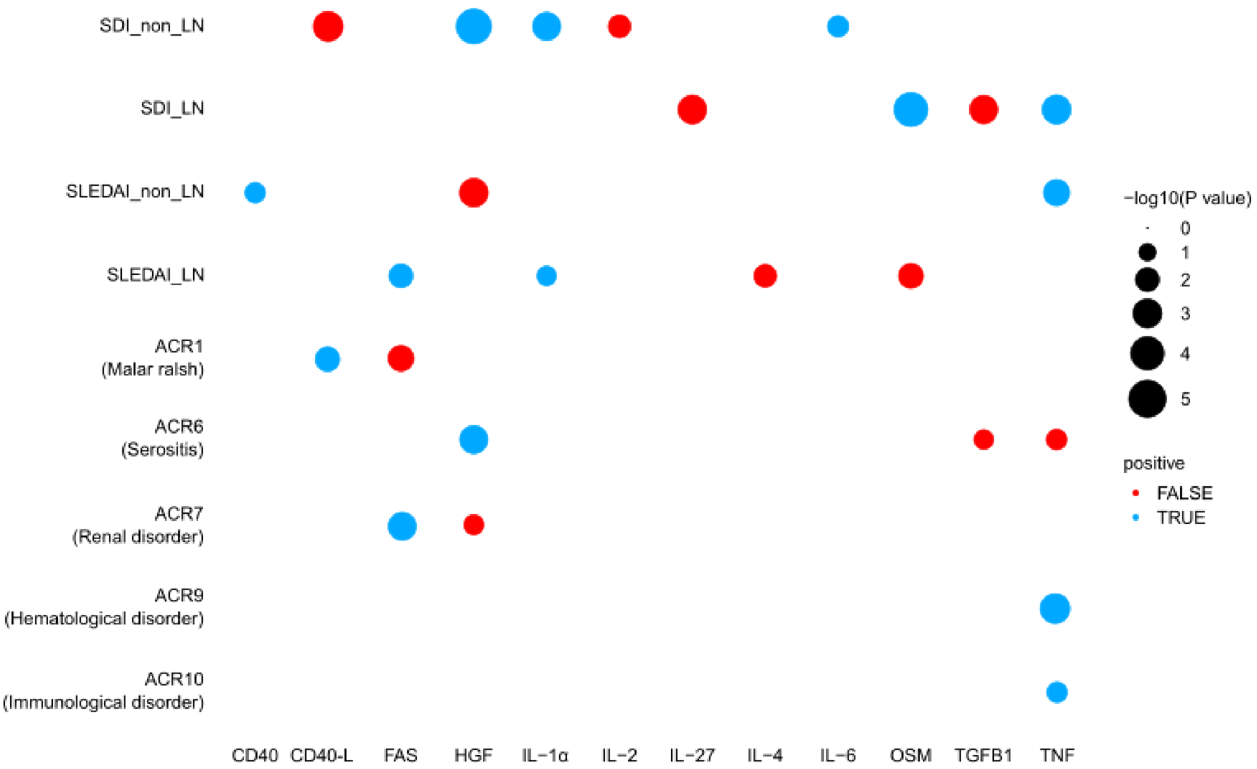

Figure S7. Summary of regression models. Related to the STAR methods.
The URs are presented in columns and the disease types are presented in rows. Blue represents a positive relationship between UR and patient phenotype. Red represents a negative relationship. The size of a dot represents the level of significance (related to Data S1_ACR1_6_7_9_10, S1_SDI and S1_SLEDAI).

Supplementary Tables:

Table S1. The marker genes used for cell type identification. Related to STAR methods and Figure 2.

Cell type	Marker genes	References
B cells	*Ms4a1, Cd22, Cd19*	[S2]
Dendritic cells	*Cebpa, Nfe2, Ifit1, Cd7, SiglecH*	[S3-6]
Endothelial cells	*Cdh5, Pecam1, Fabp4*	*[S7]*
Erythrocytes	*Alas2, Car2, Cpox, Hba-a1, Hbb-bt, Alad, Tfrc, Hba-a2*	[S8]
Fibroblasts	*Col1a2, Col3a1, Fbln2, Fstl1, Gsn, Mmp2, Sparc, Vim*	[S9]
Granulocytes	*Camp, Ngp, S100a8, S100a9*	[S10]
Macrophages	*C1qb, C1qc, Cd14, Cd68, Ctss, Cxcl2, F13a1, Gatm, Lyz2, Rgs1*	[S11]
Monocytes	*Ccl6, Cxcl3, Il1b, F13a1, Ccr2*	[S4,8]
NK cells	*Klrk1, Il18rap, Gzma*	[S12]
T cells	*Bcl11b, Il7r, Itk, Lef1*	[S13]
Myocytes	*Bgn, Rgs16, Palld, Rgs5, Cpe, Sgce, Fhl1, Cd151, Adi1, Adgrf5*	[S2,14]
Basal III cells	*Krt14, Krt5, Mt2, BC100530, Fosl1, Ptgs2*	[S15]
Neutrophils	*Cd14, S100a8, S100a9, Ly6g, Mmp9, Ltf*	[S2,16,17]

References:

S1. Li, X., Lee, E.J., Lilja, S., Loscalzo, J., Schäfer, S., Smelik, M., Strobl, M.R., Sysoev, O., Wang, H., Zhang, H., et al. (2022). A dynamic single cell-based framework for digital twins to prioritize disease genes and drug targets. Genome Med *14*, 48. 10.1186/s13073-022-01048-4.

S2. Giordani, L., He, G.J., Negroni, E., Sakai, H., Law, J.Y.C., Siu, M.M., Wan, R., Corneau, A., Tajbakhsh, S., Cheung, T.H., and Le Grand, F. (2019). High-Dimensional Single-Cell Cartography Reveals Novel Skeletal Muscle-Resident Cell Populations. Mol Cell *74*, 609-621 e606. 10.1016/j.molcel.2019.02.026.

S3. Paul, F., Arkin, Y., Giladi, A., Jaitin, D.A., Kenigsberg, E., Keren-Shaul, H., Winter, D., Lara-Astiaso, D., Gury, M., Weiner, A., et al. (2015). Transcriptional Heterogeneity and Lineage Commitment in Myeloid Progenitors. Cell *163*, 1663-1677. 10.1016/j.cell.2015.11.013.

S4. Jaitin, D.A., Weiner, A., Yofe, I., Lara-Astiaso, D., Keren-Shaul, H., David, E., Salame, T.M., Tanay, A., van Oudenaarden, A., and Amit, I. (2016). Dissecting Immune Circuits by Linking CRISPR-Pooled Screens with Single-Cell RNA-Seq. Cell *167*, 1883-1896 e1815. 10.1016/j.cell.2016.11.039.

S5. Qie, C., Jiang, J., Liu, W., Hu, X., Chen, W., Xie, X., and Liu, J. (2020). Single-cell RNA-Seq reveals the transcriptional landscape and heterogeneity of skin macrophages in Vsir(-/-) murine psoriasis. Theranostics *10*, 10483-10497. 10.7150/thno.45614.

S6. Giladi, A., Paul, F., Herzog, Y., Lubling, Y., Weiner, A., Yofe, I., Jaitin, D., Cabezas-Wallscheid, N., Dress, R., Ginhoux, F., et al. (2018). Single-cell characterization of haematopoietic progenitors and their trajectories in homeostasis and perturbed haematopoiesis. Nat Cell Biol *20*, 836-846. 10.1038/s41556-018-0121-4.

S7. Kalucka, J., de Rooij, L., Goveia, J., Rohlenova, K., Dumas, S.J., Meta, E., Conchinha, N.V., Taverna, F., Teuwen, L.A., Veys, K., et al. (2020). Single-Cell Transcriptome Atlas of Murine Endothelial Cells. Cell *180*, 764-779 e720. 10.1016/j.cell.2020.01.015.

S8. Tusi, B.K., Wolock, S.L., Weinreb, C., Hwang, Y., Hidalgo, D., Zilionis, R., Waisman, A., Huh, J.R., Klein, A.M., and Socolovsky, M. (2018). Population snapshots predict early haematopoietic and erythroid hierarchies. Nature *555*, 54-60. 10.1038/nature25741.

S9. Gladka, M.M., Molenaar, B., de Ruiter, H., van der Elst, S., Tsui, H., Versteeg, D., Lacraz, G.P.A., Huibers, M.M.H., van Oudenaarden, A., and van Rooij, E. (2018). Single-Cell Sequencing of the Healthy and Diseased Heart Reveals Cytoskeleton-Associated Protein 4 as a New Modulator of Fibroblasts Activation. Circulation *138*, 166-180. 10.1161/circulationaha.117.030742.

S10. Cochain, C., Vafadarnejad, E., Arampatzi, P., Pelisek, J., Winkels, H., Ley, K., Wolf, D., Saliba, A.E., and Zernecke, A. (2018). Single-Cell RNA-Seq Reveals the Transcriptional Landscape and Heterogeneity of Aortic Macrophages in Murine Atherosclerosis. Circ Res *122*, 1661-1674. 10.1161/CIRCRESAHA.117.312509.

S11. Farmer, D.T., Nathan, S., Finley, J.K., Shengyang Yu, K., Emmerson, E., Byrnes, L.E., Sneddon, J.B., McManus, M.T., Tward, A.D., and Knox, S.M. (2017). Defining epithelial cell dynamics and lineage relationships in the developing lacrimal gland. Development *144*, 2517-2528. 10.1242/dev.150789.

S12. Bezman, N.A., Kim, C.C., Sun, J.C., Min-Oo, G., Hendricks, D.W., Kamimura, Y., Best, J.A., Goldrath, A.W., Lanier, L.L., and Immunological Genome Project, C. (2012). Molecular definition of the identity and activation of natural killer cells. Nat Immunol *13*, 1000-1009. 10.1038/ni.2395.

S13. Park, J., Shrestha, R., Qiu, C., Kondo, A., Huang, S., Werth, M., Li, M., Barasch, J., and Susztak, K. (2018). Single-cell transcriptomics of the mouse kidney reveals potential cellular targets of kidney disease. Science *360*, 758-763. 10.1126/science.aar2131.

S14. Rubenstein, A.B., Smith, G.R., Raue, U., Begue, G., Minchev, K., Ruf-Zamojski, F., Nair, V.D., Wang, X., Zhou, L., Zaslavsky, E., et al. (2020). Single-cell transcriptional profiles in human skeletal muscle. Sci Rep *10*, 229. 10.1038/s41598-019-57110-6.

S15. Haensel, D., Jin, S., Sun, P., Cinco, R., Dragan, M., Nguyen, Q., Cang, Z., Gong, Y., Vu, R., MacLean, A.L., et al. (2020). Defining Epidermal Basal Cell States during Skin Homeostasis and Wound Healing Using Single-Cell Transcriptomics. Cell Rep *30*, 3932-3947 e3936. 10.1016/j.celrep.2020.02.091.

S16. Volberding, P.J., Xin, G., Kasmani, M.Y., Khatun, A., Brown, A.K., Nguyen, C., Stancill, J.S., Martinez, E., Corbett, J.A., and Cui, W. (2021). Suppressive neutrophils require PIM1 for metabolic fitness and survival during chronic viral infection. Cell Rep *35*, 109160. 10.1016/j.celrep.2021.109160.

S17. Grieshaber-Bouyer, R., Radtke, F.A., Cunin, P., Stifano, G., Levescot, A., Vijaykumar, B., Nelson-Maney, N., Blaustein, R.B., Monach, P.A., Nigrovic, P.A., and ImmGen, C. (2021). The neutrotime transcriptional signature defines a single continuum of neutrophils across biological compartments. Nat Commun *12*, 2856. 10.1038/s41467-021-22973-9.
